# North–South Disparities in Multimorbidity Revealed by Association Rule and Sequential Pattern Mining: Evidence From the China National Health Survey (CNHS)

**DOI:** 10.2188/jea.JE20260009

**Published:** 2026-11-05

**Authors:** Zhili Chen, Yaoda Hu, Huijing He, Qiong Ou, Yawen Liu, Tan Xu, Ji Tu, Zhiming Lu, Zichao Wang, Da Xu, Qing Wang, Zhujie Ran, Mengwei Zhang, Guangliang Shan

**Affiliations:** 1 Department of Epidemiology and Statistics, Institute of Basic Medical Sciences & School of Basic Medicine, Chinese Academy of Medical Sciences & Peking Union Medical College, Beijing, China; 2 State Key Laboratory of Common Mechanism Research for Major Diseases, Beijing, China; 3 Sleep Center, Department of Respiratory and Critical Care Medicine, Guangdong Provincial People’s Hospital (Guangdong Academy of Medical Sciences), Southern Medical University, Guangzhou, China; 4 Department of Epidemiology and Biostatistics, School of Public Health of Jilin University, Changchun, China; 5 Department of Epidemiology, School of Public Health, Jiangsu Key Laboratory of Preventive and Translational Medicine for Geriatric Diseases, MOE Key Laboratory of Geriatric Diseases and Immunology, Suzhou Medical College of Soochow University, Suzhou, China; 6 Precision and Intelligence Medical Imaging Lab, Beijing Friendship Hospital, Capital Medical University, Beijing, China; 7 School of Population Medicine and Public Health, Chinese Academy of Medical Sciences and Peking Union Medical College, Beijing, China

**Keywords:** China, multimorbidity, regional disparities, association rule mining, sequential pattern mining

## Abstract

**Background:**

North-south differences in context and lifestyle may shape multimorbidity patterns in China. We compared multimorbidity between two northern (Liaoning, Jilin) and two southern (Guangdong, Jiangsu) CNHS provinces in eastern China using association rule mining (ARM) and sequential pattern mining (SPM).

**Methods:**

We analyzed 19,067 adults (≥20 years) and 20 chronic conditions. ARM identified co-occurrence rules and reported support (proportion with the combination), confidence (P(consequent|antecedent)), and lift (observed/expected co-occurrence; >1 indicates positive association). SPM summarized common diagnosis-order sequences using sequence support (percentage of participants exhibiting an ordered sequence). Sensitivity analyses were restricted to self-reported physician diagnoses.

**Results:**

Multimorbidity prevalence was 37.85% (age-standardized 32.84%), higher in northern than southern provinces (41.39% vs 34.08%; standardized 35.41% vs 31.04%). Hypertension, hyperuricemia, and diabetes were most common. Cardiovascular/cerebrovascular conditions were more frequent in the north, whereas anemia and kidney stones were more common in the south. ARM suggested metabolic–renal clustering in the south (eg, CKD→gout, support = 1.18%, confidence = 26.14%, lift = 2.60; gout+anemia→CKD, support = 0.55%, confidence = 38.89%, lift = 3.71) and musculoskeletal–cardiovascular links in the north (eg, osteoarthritis→spinal disease, support = 0.52%, confidence = 24.69%, lift = 2.22; CTRD→CHD, support = 0.63%, confidence = 28.24%, lift = 1.26). Common SPM sequences included hypertension→hyperuricemia (24.22%) and hypertension→diabetes→hyperuricemia (5.32%); hypertension→hyperuricemia→CKD was more frequent in the south (2.28%), while hypertension→CHD→hyperuricemia was more frequent in the north (5.70%). Sensitivity analyses showed broadly consistent regional contrasts.

**Conclusion:**

Multimorbidity burden and structure differed between the northern and southern provinces, supporting province-tailored prevention and integrated care.

## INTRODUCTION

With rapid socioeconomic change and longer life expectancy, multimorbidity has become a major global public health challenge. It is usually defined as the coexistence of two or more chronic conditions in the same individual.^1^ Evidence shows that multimorbidity places a heavy strain on healthcare systems,^2^ reduces quality of life,^3^ and increases mortality.^4^ Importantly, risk is shaped not only by how many conditions a person has, but also by how those conditions cluster and interact.^5^^,^^6^ Understanding these patterns is therefore essential for effective prevention and care.

Recent meta-analyses suggest that about 37.2% of adults worldwide live with multimorbidity (95% confidence interval [CI], 34.9–39.4%).^7^ In China, the estimated prevalence is lower, at 25.4% (95% CI, 15.1–35.7%),^8^ yet the absolute burden is substantial given the country’s large and rapidly aging population. China’s wide regional diversity further complicates the picture. For instance, within eastern China, northern provinces (such as Liaoning and Jilin) and southern provinces (such as Guangdong and Jiangsu) present stark socioeconomic and environmental contrasts. The northern provinces are characterized by colder climates, diets typically higher in sodium, and economies historically reliant on heavy industry. In contrast, the southern provinces feature warmer climates, distinct dietary traditions, and highly developed, commerce-driven economic structures.^9^^–^^11^ These differences may lead to distinct multimorbidity profiles. While earlier studies have described overall patterns and urban–rural gaps,^12^^,^^13^ evidence comparing multimorbidity patterns across distinct provincial settings remains limited.^14^

To explore how chronic diseases cluster, association rule mining (ARM) has been widely used to identify common disease combinations based on measures such as support, confidence, and lift.^15^^–^^17^ Yet ARM is limited by its lack of temporal information. Sequential pattern mining (SPM), by contrast, focuses on the order in which diseases are diagnosed. By utilizing retrospective chronological data, such as self-reported diagnosis dates, SPM can shed light on common diagnostic ordering patterns.^18^ So far, most SPM studies rely on clinical or hospital-based data,^19^^–^^21^ leaving population-based evidence limited.

Against this background, we used data from the China National Health Survey (CNHS) to examine north-south disparities in multimorbidity between four specific provinces in eastern China (Liaoning and Jilin in the north, compared to Guangdong and Jiangsu in the south). By combining ARM and SPM, we aimed to capture both disease co-occurrence and diagnosis-order sequences across regions. These insights may help inform region-specific prevention strategies and support more tailored approaches to multimorbidity management.

## METHODS

### Study design and participants

Data were derived from the CNHS, a large-scale, community-based, cross-sectional study launched in 2012. The CNHS was originally established as a multidisciplinary national health examination program aimed at developing population-specific reference intervals for key physiological and biochemical indicators and creating a standardized health database to support research on health determinants and noncommunicable diseases.^22^ The survey employed a multistage, stratified cluster sampling method to recruit adults aged 20 years and older. To be eligible, participants were required to be stable residents with a local registered household identity (hukou) and to have resided in the local area for at least 5 years prior to the survey. For the current analysis, we utilized data collected between April 2023 and October 2024 from four specific provinces in eastern China. For analytical purposes, we grouped Guangdong and Jiangsu as the “southern provinces,” and Liaoning and Jilin as the “northern provinces.” This categorization reflects a province-based grouping within the CNHS sampling framework and does not imply a nationwide north-south division of China.

A total of 19,243 Han Chinese adults completed the survey (eFigure 1). We excluded participants younger than 20 years (*n* = 12) and those without a prior diagnosis of hypertension, anemia, diabetes, hyperuricemia, or chronic kidney disease but lacking key diagnostic data (blood pressure, hemoglobin, fasting glucose, serum uric acid, and serum creatinine) (*n* = 164). The final analytic sample included 19,067 participants.

### Data collection

Data collection was standardized across sites and conducted by trained staff using face-to-face interviews, physical exams, and laboratory tests. Questionnaires captured sociodemographics, lifestyle factors, and personal/family medical history. Height was measured barefoot with a stadiometer (Seca 285; Seca, Hamburg, Germany). Weight and body composition were assessed by bioelectrical impedance (TANITA MC-780MA; Tanita, Tokyo, Japan). Blood pressure was measured three times (right arm, seated) using an automatic device (Omron HEM-907; OMRON, Kyoto, Japan); the mean was used. After ≥8 hours fasting, serum uric acid, fasting plasma glucose (FPG), creatinine, and hemoglobin were assayed with a AU5821 Clinical Chemistry Analyzer (Beckman Coulter, Brea, CA, USA) and a XN-10[B3] Hematology Analyzer (SYSMEX, Kobe, Japan).

### Definition and measurement of multimorbidity

Following the World Health Organization’s definition,^23^ multimorbidity was defined as the co-occurrence of two or more chronic conditions in the same individual. The selection of chronic conditions was informed by the following sources: the Delphi consensus on multimorbidity research,^24^ a Lancet review that proposed a core list of chronic conditions for multimorbidity measurement,^25^ and the top 25 causes of disability-adjusted life years in China.^26^ Based on these references and the availability of data in this study, a total of 20 chronic conditions were included: hypertension, diabetes, hyperuricemia, gout, chronic kidney disease (CKD), anemia, stroke, coronary heart disease (CHD), arrhythmia, thyroid disorders (hyperthyroidism/hypothyroidism), chronic obstructive pulmonary disease (COPD), asthma, cancer, connective tissue and rheumatic diseases (CTRD), osteoarthritis, spinal disease, chronic liver disease (CLD), gallbladder diseases (gallstones/cholecystitis), chronic gastritis, and kidney stones.

Disease status and first diagnosis date were self-reported, based on diagnoses made at county-level hospitals or higher. For each condition, only the earliest diagnosis date was retained to define disease onset, and conditions diagnosed at the same time were treated as concurrent events without assumed directional sequence. For five conditions (hypertension, diabetes, hyperuricemia, CKD, and anemia) diagnoses were also confirmed through physical measurements and laboratory tests during the survey. Hypertension was defined as mean systolic blood pressure ≥140 mm Hg and/or diastolic blood pressure ≥90 mm Hg.^27^ Diabetes was defined as FPG ≥7.0 mmol/L.^28^ Hyperuricemia was defined as serum uric acid >360 µmol/L in women and >420 µmol/L in men.^29^ CKD was defined as an estimated glomerular filtration rate (eGFR) <60 mL·min^−1^·(1.73 m^2^)^−1^, calculated from serum creatinine.^30^^,^^31^ Anemia was defined as hemoglobin <130 g/L in men and <120 g/L in women.^32^ If an individual lacked a prior diagnosis but met the above criteria during survey, the investigation date was recorded as the diagnosis date. All conditions were coded as binary variables (yes/no).

### Statistical analysis

Data are expressed as means (standard deviations [SDs]), medians (interquartile ranges [IQRs]), or frequencies (%). Group differences were assessed using Student’s *t*-tests, Mann-Whitney U tests, or chi-square tests, as appropriate. Multimorbidity prevalence and 95% CIs were calculated via the Wilson method and age-standardized to the 2020 China Population Census Yearbook. To account for potential confounding, multivariable logistic regression was performed to evaluate the independent association between the regional setting and the presence of multimorbidity, adjusting for age, sex, body mass index (BMI), and other relevant demographic and lifestyle factors.

ARM was employed to identify frequent and significant disease co-occurrences in northern and southern provinces. ARM is an unsupervised data mining technique used to discover hidden associations between co-occurring diseases. Each rule consists of an antecedent and a consequent, representing a conditional relationship in the form: “if the antecedent exists, then the consequent is likely to occur”.^15^ We examined both binary (two-disease) and ternary (three-disease) association rules to capture both common and complex multimorbidity combinations. Rules were evaluated using three key metrics: (1) support, the proportion of records containing a given disease combination; (2) confidence, the conditional probability of the consequent given the antecedent; and (3) lift, the ratio of observed co-occurrence to expected co-occurrence under independence, where a value greater than 1 indicates a positive association. A higher lift value suggests a stronger co-occurrence and more clinically meaningful association.^16^ An a priori algorithm was first applied using a minimal support threshold of 0.1% (0.001) to extract all potentially relevant rules, without restricting confidence. Subsequently, more stringent thresholds were used to refine the selection of important patterns: support >0.5%, confidence >20%, and lift >1. Qualified binary and ternary rules were ranked by lift, and the top 10 rules with the highest lift were reported for each region.

SPM was employed to uncover frequent diagnostic ordering patterns in northern and southern provinces. To establish temporal ordering, diagnosis-order sequences were constructed for each participant using the self-reported date of first diagnosis or clinical encounter. These time-ordered sequences served as the basis for the temporal analysis of multimorbidity.^19^ We implemented the Sequential Pattern Discovery using Equivalence classes algorithm, a computationally efficient method capable of identifying frequent subsequences from large-scale sequence databases.^33^ To enhance the clinical interpretability and statistical reliability of the results, we set the minimum support threshold at 1%, retaining only those sequences that occurred in at least 1% of the sample. Both binary (two-event) and ternary (three-event) sequential patterns were extracted to explore typical diagnostic sequences of multimorbidity. All valid sequences were ranked by support, and the top 10 sequences with the highest support were reported.

We conducted stratified analyses by age (20–44, 45–64, ≥65 years) and sex within northern and southern provinces using ARM and SPM. Within each regional stratum, binary and ternary association rules and sequential patterns were extracted to identify subgroup-specific disease combinations and diagnostic ordering sequences. Finally, to address the potential inflation of disease prevalence from single-time-point survey measurements, a sensitivity analysis was conducted. We re-ran all primary analyses restricted strictly to cases with self-reported physician diagnoses.

All analyses were conducted using SAS 9.4 (SAS Institute Inc., Cary, NC, USA) and R version 4.4.3 (R Foundation for Statistical Computing, Vienna, Austria).

## RESULTS

### Participant characteristics

A total of 19,067 participants were included in the analysis, comprising 9,908 from southern and 9,159 from northern provinces (Table 1). The overall mean age was 54.0 (SD, 12.7) years. Compared to the south, northern participants had a higher proportion of older adults (≥65 years), higher educational attainment, and fewer rural residents. Regarding lifestyle, both physical activity and obesity prevalence were greater in the northern provinces. Importantly, multivariable logistic regression showed that residing in the northern provinces remained significantly associated with higher odds of multimorbidity compared with the south, even after full adjustment for these demographic and lifestyle factors (adjusted OR 1.17; 95% CI, 1.10–1.25; eTable 1).

**Table 1.** Characteristics of the overall population and by region

Characteristics	Total	Southern provinces	Northern provinces	*P*-value
***N***	19,067	9,908	9,159	
**Age, years**				
Mean (SD)	54.0 (12.7)	52.0 (12.6)	56.3 (12.4)	<0.0001
Median (IQR)	55 (46, 63)	53 (43, 61)	58 (49, 65)	<0.0001
**Age group, *n* (%)**				
20–44 years	4,380 (22.97)	2,770 (27.96)	1,610 (17.58)	<0.0001
45–64 years	10,545 (55.31)	5,480 (55.31)	5,065 (55.30)	
≥65 years	4,142 (21.72)	1,658 (16.73)	2,484 (27.12)	
**Sex, *n* (%)**				
Male	7,070 (37.08)	3,884 (39.20)	3,186 (34.79)	<0.0001
Female	11,997 (62.92)	6,024 (60.80)	5,973 (65.21)	
**Education attainment, *n* (%)**				
Primary or below	3,651 (19.15)	2,089 (21.08)	1,562 (17.05)	<0.0001
Middle School	6,206 (32.55)	3,208 (32.38)	2,998 (32.73)	
High School	4,235 (22.21)	2,041 (20.60)	2,194 (23.95)	
College or above	4,975 (26.09)	2,570 (25.94)	2,405 (26.27)	
**Marital status, *n* (%)**				
Unmarried/Divorced/Widowed	2,303 (12.08)	951 (9.60)	1,352 (14.76)	<0.0001
Married	16,764 (87.92)	8,957 (90.40)	7,807 (85.24)	
**Residence, *n* (%)**				
Urban	12,311 (64.57)	5,997 (60.53)	6,314 (68.94)	<0.0001
Rural	6,756 (35.43)	3,911 (39.47)	2,845 (31.06)	
**Smoking status, *n* (%)**				
Never	14,410 (75.58)	7,652 (77.23)	6,758 (73.79)	<0.0001
Former	1,410 (7.39)	626 (6.32)	784 (8.56)	
Current	3,247 (17.03)	1,630 (16.45)	1,617 (17.65)	
**Alcohol consumption, *n* (%)**				
Never	13,821 (72.49)	7,160 (72.26)	6,661 (72.73)	0.5011
Former	777 (4.08)	395 (3.99)	382 (4.17)	
Current	4,469 (23.43)	2,353 (23.75)	2,116 (23.10)	
**Physical activity level,**^a^ ***n* (%)**				
Low	4,322 (22.67)	2,430 (24.53)	1,892 (20.66)	<0.0001
Moderate	12,919 (67.76)	6,487 (65.47)	6,432 (70.23)	
High	1,826 (9.57)	991 (10.00)	835 (9.11)	
**BMI group,**^b^ ***n* (%)**				
Underweight	521 (2.73)	312 (3.15)	209 (2.28)	<0.0001
Normal	8,325 (43.66)	4,557 (45.99)	3,768 (41.14)	
Overweight	7,321 (38.40)	3,696 (37.30)	3,625 (39.58)	
Obesity	2,900 (15.21)	1,343 (13.56)	1,557 (17.00)	

BMI, body mass index; IQR, interquartile ranges; SD, standard deviations.

^a^Physical activity, encompassing both occupational and leisure-time components, was classified as low (light activity in both), moderate (moderate or high activity in either), or high (moderate or high activity in both domains).

^b^BMI was calculated as weight (kg) divided by height squared (m^2^). BMI categories were defined as follows: underweight (<18.5 kg/m^2^), normal (18.5–23.9 kg/m^2^), overweight (24.0–27.9 kg/m^2^), and obesity (≥28.0 kg/m^2^).

### Prevalence of chronic conditions and multimorbidity combinations

Overall, multimorbidity affected 37.85% of participants (Figure 1 and eTable 2) and was higher in the northern than southern provinces (41.39% vs 34.08%, *P* < 0.001), including after age standardization (35.41% vs 31.04%). Men had higher prevalence than women (46.46% vs 32.77%), and the regional gap persisted in both sexes. Hypertension, hyperuricemia, and diabetes were most common. Cardiovascular conditions (CHD, stroke, arrhythmia) were more frequent in the north, whereas anemia (women) and kidney stones (men) were more common in the south. Multimorbidity rose with age and converged in older adults, but CHD and stroke remained higher in the north (eTable 3, eTable 4, eTable 5, and eTable 6). Single conditions were slightly more common in the south, while ≥2 conditions were more common in the north (eTable 7). The leading combination was hypertension–hyperuricemia, often with diabetes; clusters were more cardiovascular-oriented in the north and more CKD/gout/anemia-related in the south (eTable 8, eTable 9, eTable 10, eTable 11, eTable 12, and eTable 13).

**Figure 1.**
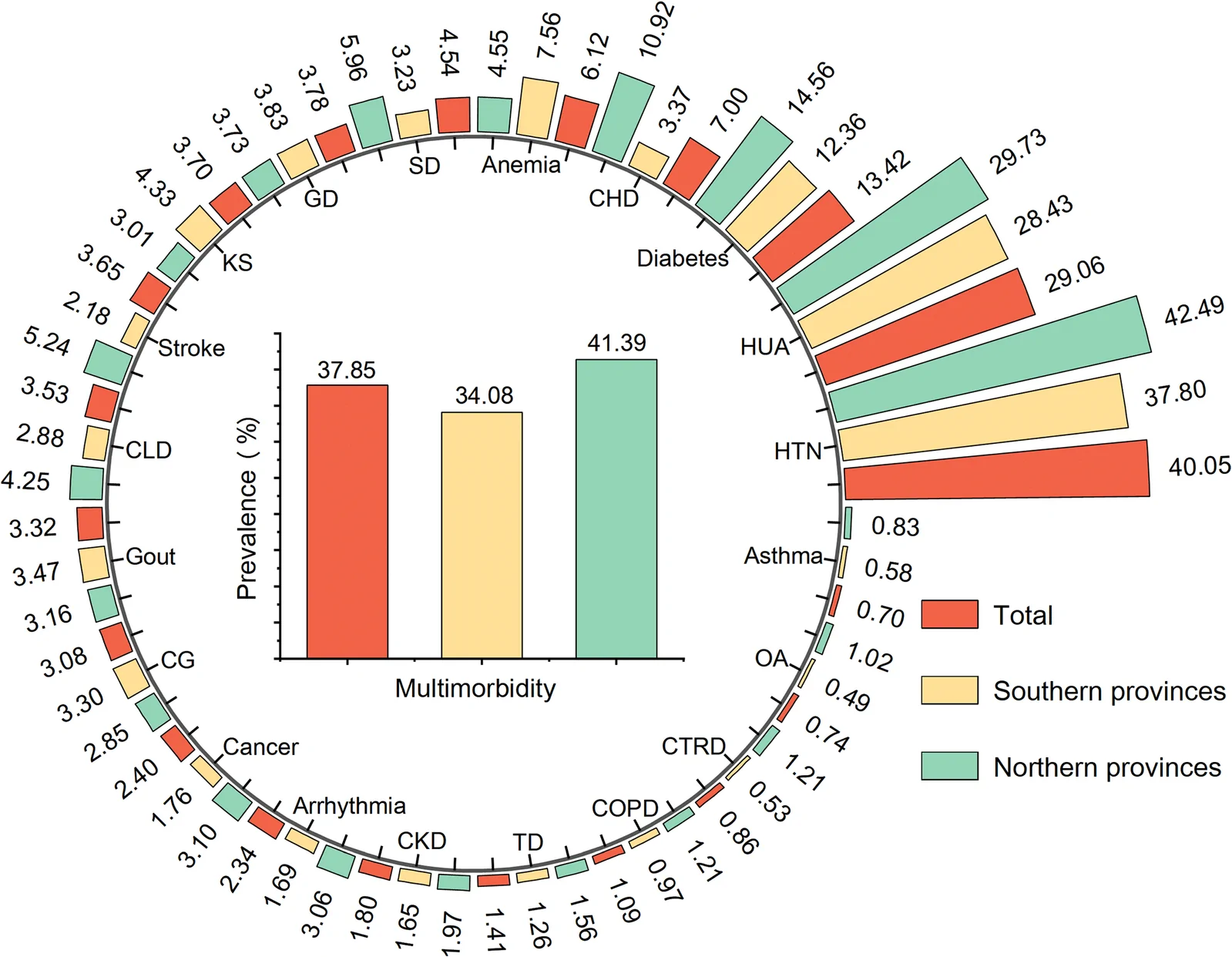
Prevalence of multimorbidity and chronic conditions by region. Conditions are arranged in counterclockwise order according to their overall prevalence from highest to lowest. The central bar chart shows the prevalence of multimorbidity. CHD, coronary heart disease; CG, chronic gastritis; CKD, chronic kidney disease; CLD, chronic liver disease; COPD, chronic obstructive pulmonary disease; CTRD, connective tissue and rheumatic diseases; GD, gallbladder diseases; HTN, hypertension; HUA, hyperuricemia; KS, kidney stones; OA, osteoarthritis; SD, spinal disease; TD, thyroid disorders.

### Association rule mining results

In the overall sample (Figure 2 and Figure 3), the strongest binary associations centered on CKD. The rules “CKD → Gout” (lift = 2.42) and “CKD → Anemia” (lift = 2.41) showed that gout and anemia often accompany kidney disease. At the same time, “Gout → Hyperuricemia” reached very high confidence (97.29%) with a clear lift (1.81), highlighting their tight link. In ternary rules, CKD frequently appeared as a consequent, especially in combinations involving hyperuricemia, diabetes, and anemia, pointing to close connections between metabolic disorders, anemia, and kidney dysfunction.

**Figure 2.**
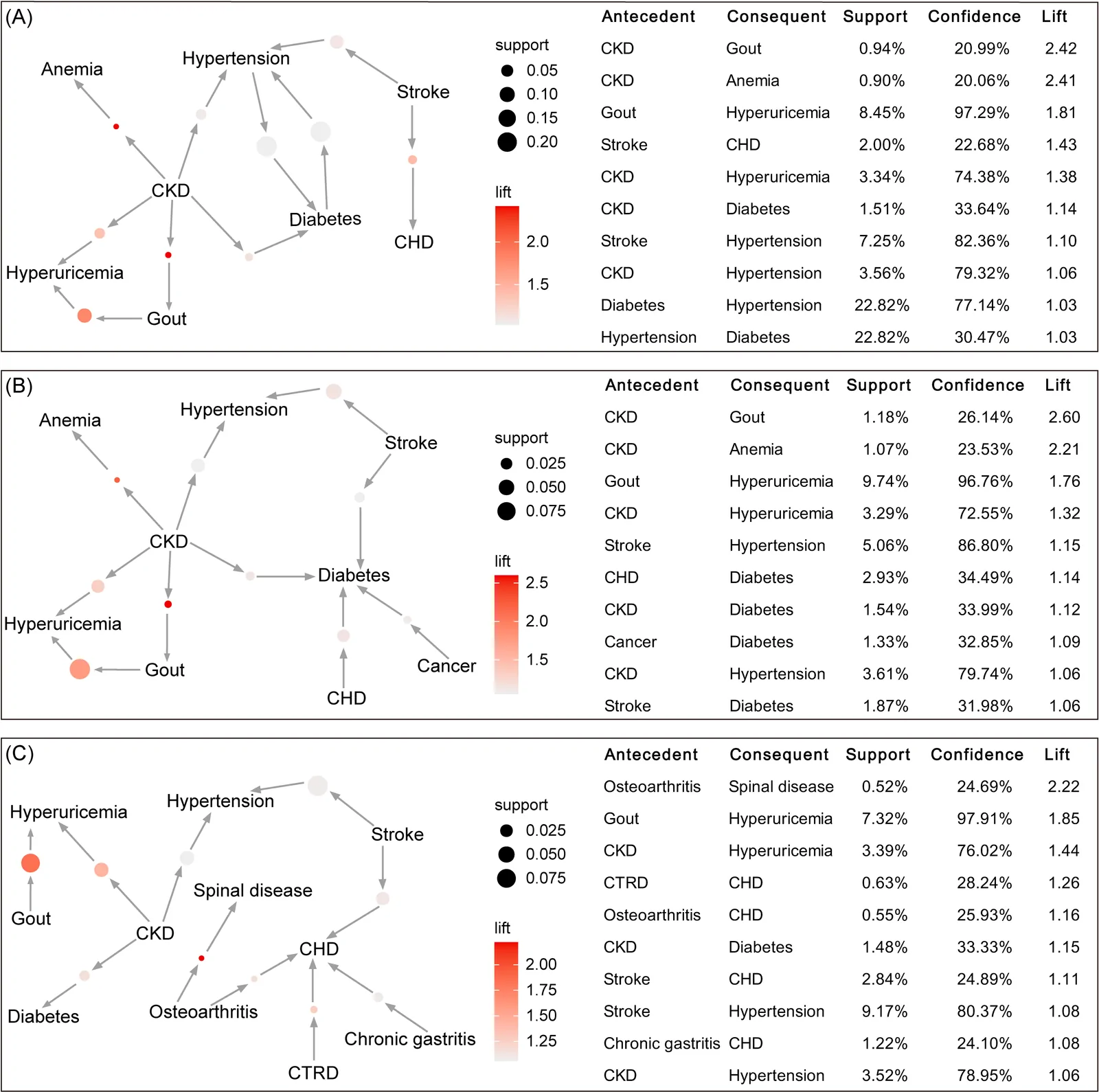
Top 10 binary association rules of multimorbidity in the overall sample and by region. Panels (**A**), (**B**), and (**C**) present the results for the overall sample, southern provinces, and northern provinces, respectively. The left panels display network graphs, where node size reflects support (ie, co-occurrence prevalence), node color intensity indicates lift, and directed edges represent the antecedent–consequent relationships. Diseases with more incoming arrows are more likely to co-occur with other conditions. The right panels list the detailed rules, including antecedent, consequent, support, confidence, and lift values. CHD, coronary heart disease; CKD, chronic kidney disease; CTRD, connective tissue and rheumatic diseases.

**Figure 3.**
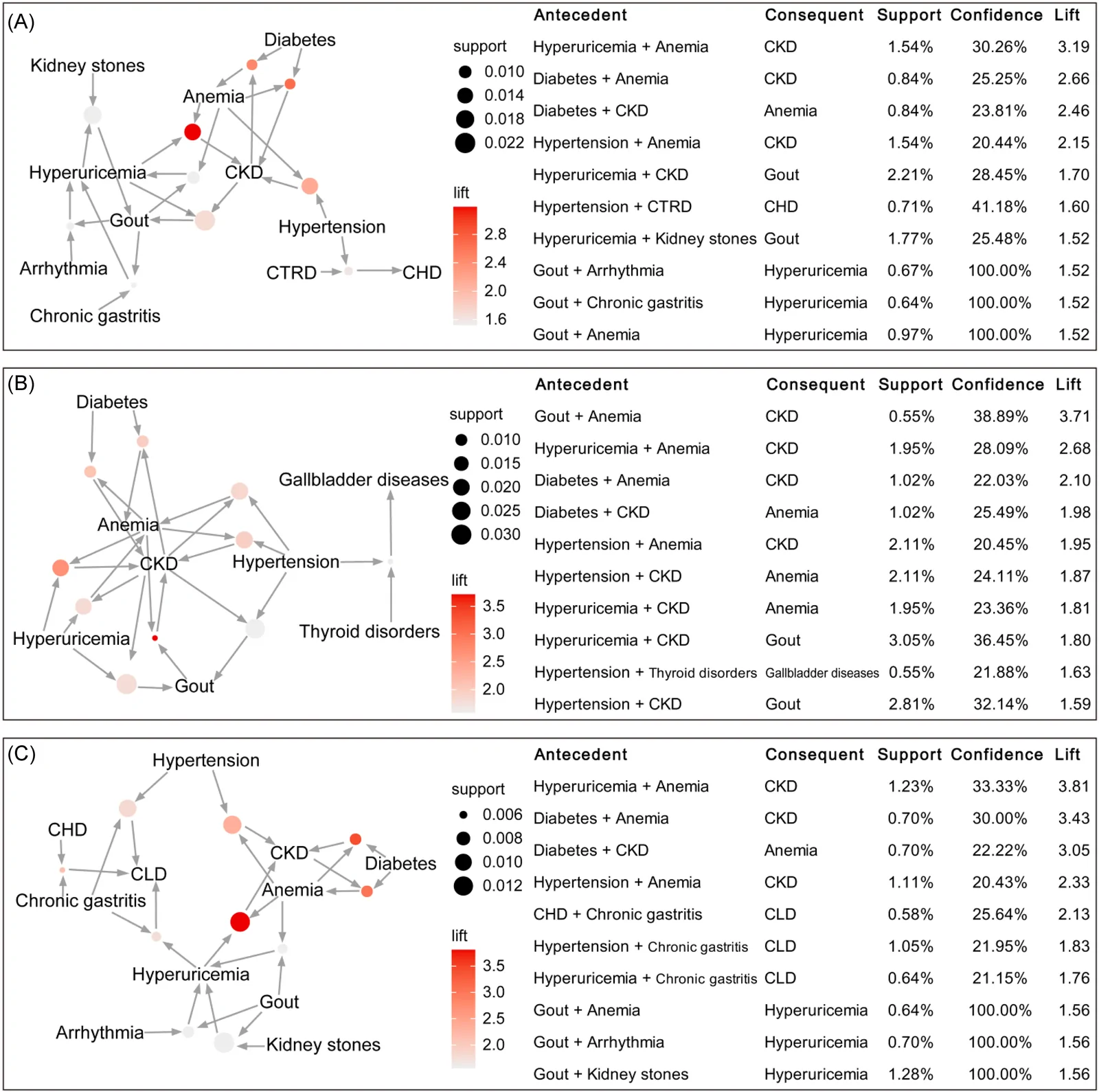
Top 10 ternary association rules of multimorbidity in the overall sample and by region. Panels (**A**), (**B**), and (**C**) present the results for the overall sample, southern provinces, and northern provinces, respectively. The left panels display network graphs, where node size reflects support (ie, co-occurrence prevalence), node color intensity indicates lift, and directed edges represent the antecedent–consequent relationships. Diseases with more incoming arrows are more likely to co-occur with other conditions. The right panels list the detailed rules, including antecedent, consequent, support, confidence, and lift values. CKD, chronic kidney disease; CHD, coronary heart disease; CTRD, connective tissue and rheumatic diseases; CLD, chronic liver disease.

Clear regional differences emerged. In the southern provinces, CKD-associated patterns were more prominent, including “CKD → Gout” (lift = 2.60) and “CKD → Anemia” (lift = 2.21). The ternary rule “Gout + Anemia → CKD” also demonstrated particularly strong lift in this region. Conversely, the northern provinces exhibited stronger associations involving musculoskeletal, immune, and cardiovascular conditions, such as “Osteoarthritis → Spinal disease” and “CTRD → CHD,” alongside mixed-system ternary patterns combining cardiometabolic and digestive diseases.

Age- and sex-stratified analyses (eTable 14, eTable 15, eTable 16, eTable 17, eTable 18, eTable 19, eTable 20, eTable 21, eTable 22, eTable 23, and eTable 24) reinforced these regional disparities. In the southern provinces, patterns progressed from thyroid, gallbladder, and kidney stone associations in younger adults to prominent CKD-centered clusters in middle and older age. Conversely, the northern provinces shifted from gastrointestinal and diabetes-related links in youth to distinct musculoskeletal, cardiovascular, and digestive cross-system patterns in later life. While CKD, gout, and anemia clustered among older adults across all studied provinces, sex-specific analyses consistently mirrored these broader regional trends.

### Sequential pattern mining results

In the overall sample (Figure 4), the most common diagnosis-order sequences predominantly featured metabolic conditions. Hypertension often came first, followed by hyperuricemia (24.22%) or diabetes (10.66%), and diabetes itself was frequently followed by hyperuricemia (6.76%). Among ternary sequences, the ordering of “Hypertension → Diabetes → Hyperuricemia” was the most common (5.32%).

**Figure 4.**
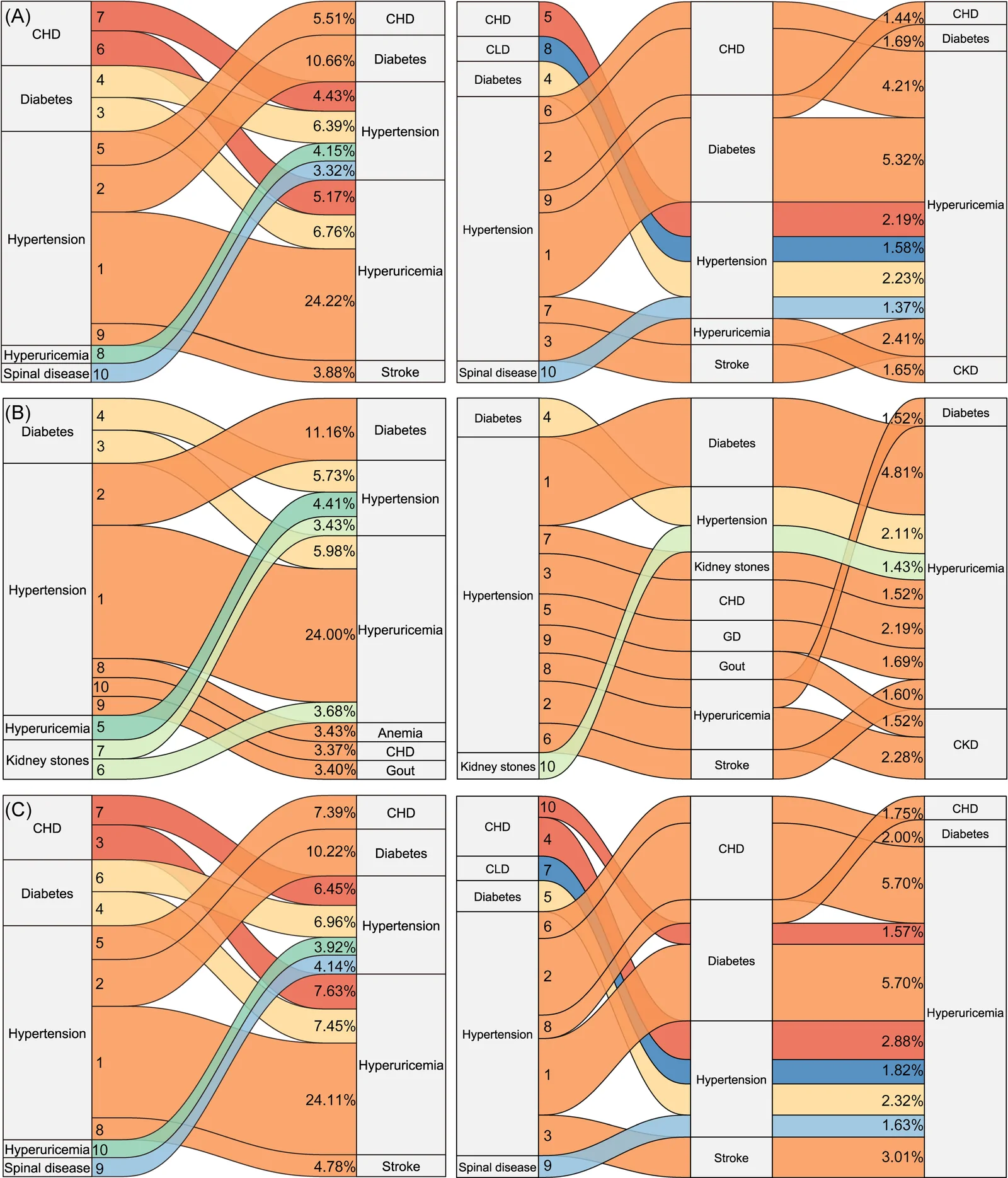
Top 10 binary and ternary sequential patterns of multimorbidity in the overall sample and by region. The Sankey diagrams display the ten most frequent sequential patterns of disease. Panels (**A**), (**B**), and (**C**) present the results for the overall sample, southern provinces, and northern provinces, respectively. Each flow represents a binary or ternary sequence, with its rank and corresponding support (%) labeled alongside. The width of the bands is proportional to the support, reflecting the proportion of individuals with the given sequence in the population. Left panels show binary sequences, while right panels present ternary sequences. CHD, coronary heart disease; CKD, chronic kidney disease; CLD, chronic liver disease.

Clear regional differences appeared within these metabolic sequences. In the southern provinces, diagnostic patterns frequently linked metabolic and renal conditions, such as “Kidney stones → Hyperuricemia/Hypertension” and “Hypertension → Anemia.” These extended to renal-focused diagnostic sequences, including “Hypertension → Hyperuricemia/Gout → CKD.” In the northern provinces, diagnostic sequences more often shifted toward the cardiovascular system, including “Hypertension → Stroke,” “CHD → Hyperuricemia,” and “Hypertension → Diabetes → CHD.”

Age- and sex-stratified analyses (eTable 25, eTable 26, eTable 27, eTable 28, and eTable 29) reinforced these regional contrasts. While the “Hypertension → Hyperuricemia” sequence dominated across all age groups, younger and middle-aged adults in the southern provinces exhibited more metabolic–renal diagnostic sequences, whereas the northern provinces displayed more cardiovascular-oriented sequences. Sex-specific analyses consistently mirrored these distinct regional trends.

### Sensitivity analysis

Restricting disease definitions to self-reported physician diagnoses reduced overall multimorbidity prevalence to 23.56% (eTable 2), mainly because many hyperuricemia, CKD, and anemia cases were detected only in survey measurements (eTable 30). Regional disparities persisted, with higher prevalence in the northern than southern provinces (27.38% vs 20.02%). The association between northern residence and multimorbidity also remained and was slightly stronger (adjusted OR 1.25; 95% CI, 1.17–1.35; eTable 1).

Excluding survey-detected cases reduced some asymptomatic CKD-centered rules, but the core regional clustering remained (ARM; eTable 31). The southern provinces continued to show metabolic and anemia-related clusters (eg, “Anemia → Arrhythmia,” lift = 3.58; “Hyperuricemia + CKD → Gout,” lift = 2.75), whereas the northern provinces showed stronger musculoskeletal and cardiovascular linkages (eg, “Osteoarthritis → Spinal disease,” lift = 1.92; “CTRD → CHD,” lift = 1.03; “Diabetes + Gout → Hyperuricemia,” lift = 2.87).

SPM also indicated region-specific diagnosis-order patterns (eTable 25, eTable 26, eTable 27, eTable 28, eTable 29, and eTable 32). The south retained metabolic–renal sequences (eg, “Hypertension → Gout,” 5.96%; “Hypertension → Diabetes → Hyperuricemia,” 2.16%), while the north showed more cardiovascular-oriented sequences (eg, “Hypertension → CHD,” 10.22%; “Hypertension → Diabetes → CHD,” 3.81%).

## DISCUSSION

Using data from four CNHS sampling provinces in eastern China, we observed a higher prevalence of multimorbidity in the northern provinces than in the southern provinces, consistent with prior reports of regional gradients in chronic disease burden.^34^ After adjusting for demographics, lifestyle factors, and BMI, residing in northern provinces remained significantly associated with higher multimorbidity odds. Importantly, the contrast was not limited to prevalence: we also observed differences in the structure of multimorbidity. Across the full sample, hypertension, diabetes, and hyperuricemia were among the most common conditions and frequently co-occurred. In the northern provinces, combinations were more cardiovascular-centered (eg, hypertension with CHD or stroke), whereas in the southern provinces, metabolic–renal conditions appeared more prominent, suggesting region-specific clustering of chronic conditions.

Several contextual factors may contribute to these province-level differences, although our data do not allow direct causal attribution. Prior studies have reported higher cardiovascular burden in northern versus southern settings,^35^^–^^37^ while renal dysfunction may show the opposite gradient in some Chinese populations.^38^^,^^39^ Differences in diet (eg, sodium intake and vegetable consumption), adiposity, and climate may plausibly shape cardiometabolic risk profiles. For example, colder environments may affect vascular tone and blood pressure control,^9^^,^^40^ while dietary patterns and humidity-related behaviors in southern settings may influence uric acid load and renal stress.^41^^,^^42^ These interpretations should be treated as hypothesis-generating and require validation with individual-level exposure data and longitudinal designs.

ARM further highlighted contrasting co-occurrence structures. In the southern provinces, CKD frequently appeared within high-lift rules alongside hypertension, diabetes, hyperuricemia, gout, and anemia, consistent with a metabolic–renal clustering pattern.^43^ In the northern provinces, rules more often involved cardiovascular, musculoskeletal, and CTRD, suggesting a broader systemic burden. Examples such as “osteoarthritis → spinal disease” and “CTRD → CHD” may reflect shared inflammatory and behavioral pathways that co-aggregate with cardiovascular risk.^44^^,^^45^ It is also important to interpret ARM directions appropriately: a rule such as “Gout → Hyperuricemia” reflects high conditional co-occurrence (eg, most people with gout have hyperuricemia), not temporal ordering or causality. This distinction is crucial to avoid misreading ARM outputs as progression pathways.

SPM complemented ARM by summarizing common diagnosis-order sequences reconstructed from reported first-diagnosis dates. In the southern provinces, sequences such as “Hypertension → Gout → CKD” were observed, whereas in the northern provinces, patterns such as “Hypertension → CHD/Stroke → Hyperuricemia” appeared more frequently. These region-specific diagnosis-order sequences may reflect differences in clinical detection, healthcare utilization, and underlying risk factor distributions.^46^ For example, a sequence such as “Hypertension → Anemia” should not be interpreted as direct biological causation. Anemia in hypertensive individuals may be mediated by undiagnosed CKD, medication effects, chronic inflammation, or increased medical surveillance following an initial diagnosis. Therefore, SPM results should be interpreted as descriptive temporal ordering patterns that are hypothesis-generating rather than causal. Comparable evidence has been reported elsewhere, a United States study based on Health and Retirement Study data identified frequent sequences such as “Osteoarthritis → Hypertension” and “Arthritis → Hypertension → Heart disease,” supporting a common diagnostic ordering from hypertension to cardiovascular disease.^47^ In China, one hospital-based electronic medical record study reported frequent endocrine–cardiovascular sequences (eg, “Hypertension → Heart failure”).^19^

Age-stratified analyses revealed a dynamic convergence in multimorbidity: regional disparities were pronounced among young adults but became negligible in the elderly. This convergence likely reflects cumulative risk exposures and aging-related physiological changes that transcend regional differences.^48^^,^^49^ In younger cohorts, northern provinces exhibited high-lift gastrointestinal clusters (eg, “Chronic gastritis–Gallbladder disease”), potentially linked to high-salt/high-fat diets and lifestyle behaviors.^50^^,^^51^ Conversely, southern provinces showed more endocrine and urinary combinations (eg, “Thyroid disorders–Anemia,” “Kidney stones–Gallbladder disease”), possibly influenced by iodine levels, mineral-rich water, or humid climates.^52^^–^^56^ Sex-stratified analyses further demonstrated that biological susceptibility and gender-specific behaviors modulate these regional patterns.^40^^,^^57^^,^^58^ Northern men showed prominent musculoskeletal-digestive and cardiovascular clusters, likely exacerbated by higher alcohol and red meat consumption.^59^^,^^60^ In contrast, southern men exhibited a renal-metabolic cluster dominated by gout and CKD. Among women, hormonal and nutritional factors likely contribute to anemia–CKD clustering,^61^ while obesity-driven inflammation and cold climate effects may underlie northern women’s musculoskeletal patterns.^62^^,^^63^ Sensitivity analyses limited to self-reported diagnoses showed broadly similar regional differences and clustering patterns. The notable prevalence reductions—especially for hyperuricemia, CKD, and anemia—may indicate a meaningful burden of previously unrecognized disease. Although single survey measurements have limitations, this contrast suggests that clinical diagnoses alone could understate community multimorbidity and overlook early, often asymptomatic high-risk clusters, supporting the potential value of proactive, integrated screening strategies.

From a clinical and public health perspective, high-prevalence clusters (hypertension–diabetes–hyperuricemia) drive much of the population burden and should inform primary prevention and chronic care. However, pattern mining is prevalence-driven and may underweight less common but high-severity overlaps. For instance, a cancer–diabetes rule was observed in the southern provinces (Cancer → Diabetes: support 1.33%, confidence 32.85%, lift = 1.09), suggesting a need for targeted surveillance despite low frequency. Region-specific priorities may differ: the southern provinces may emphasize earlier CKD detection and integrated management in people with gout/hyperuricemia, diabetes, anemia, or hypertension, whereas the northern provinces may prioritize cardiovascular risk reduction (hypertension control, CHD/stroke prevention) alongside musculoskeletal health management.

Several limitations should be acknowledged. First, diagnosis-order sequences were derived from self-reported first diagnosis dates in a cross-sectional survey and should be interpreted descriptively; recall error and diagnostic delay may affect ordering. Second, single-time-point measurements may overestimate some conditions, although sensitivity analyses using self-reported diagnoses showed similar regional contrasts. Third, limited migration-history data may introduce exposure misclassification despite the ≥5-year residency requirement. Fourth, psychiatric disorders were not included. Fifth, ARM and SPM cannot fully adjust for confounding, and the analysis was restricted to four eastern provinces, so broader generalization should be cautious. Future research should validate these patterns in longitudinal cohorts, incorporate migration and healthcare utilization data, quantify individual-level environmental and dietary exposures, integrate biospecimen-based omics, and extend analyses to finer geographic scales.

### Conclusion

By applying ARM and SPM, this study identified clear north-south disparities in multimorbidity. Multimorbidity prevalence was higher in the northern provinces, and clustering patterns differed: cardiovascular-focused combinations and diagnosis-order sequences were more prominent in the north, whereas metabolic-renal patterns featured more strongly in the south. These findings underscore the need for province-tailored prevention and chronic disease management and provide population-based evidence to inform targeted screening and integrated care strategies.

## SUPPLEMENTARY MATERIAL

eTable 1. Adjusted odds ratios for multimorbidity comparing northern and southern provinces

eTable 2. Prevalence of multimorbidity and chronic conditions by region

eTable 3. Prevalence of multimorbidity and chronic conditions among young adults (20–44 years)

eTable 4. Prevalence of multimorbidity and chronic conditions among middle-aged adults (45–64 years)

eTable 5. Prevalence of multimorbidity and chronic conditions among older adults (≥65 years)

eTable 6. Prevalence of multimorbidity and chronic conditions by sex

eTable 7. Number of chronic conditions by region

eTable 8. Top 10 binary and ternary combinations of chronic conditions by region

eTable 9. Top 10 binary and ternary combinations of chronic conditions among young adults (20–44 years)

eTable 10. Top 10 binary and ternary combinations of chronic conditions among middle-aged adults (45–64 years)

eTable 11. Top 10 binary and ternary combinations of chronic conditions among older adults (≥65 years)

eTable 12. Top 10 binary and ternary combinations of chronic conditions among men

eTable 13. Top 10 binary and ternary combinations of chronic conditions among women

eTable 14. Top 10 binary association rules of multimorbidity among young adults (20–44 years)

eTable 15. Top 10 binary association rules of multimorbidity among middle-aged adults (45–64 years)

eTable 16. Top 10 binary association rules of multimorbidity among older adults (≥65 years)

eTable 17. Top 10 ternary association rules of multimorbidity

eTable 18. Top 10 ternary association rules of multimorbidity among young adults (20–44 years)

eTable 19. Top 10 ternary association rules of multimorbidity among middle-aged adults (45–64 years)

eTable 20. Top 10 ternary association rules of multimorbidity among older adults (≥65 years)

eTable 21. Top 10 binary association rules of multimorbidity among men

eTable 22. Top 10 binary association rules of multimorbidity among women

eTable 23. Top 10 ternary association rules of multimorbidity among men

eTable 24. Top 10 ternary association rules of multimorbidity among women

eTable 25. Top 10 binary and ternary sequential patterns of multimorbidity among young adults (20–44 years)

eTable 26. Top 10 binary and ternary sequential patterns of multimorbidity among middle-aged adults (45–64 years)

eTable 27. Top 10 binary and ternary sequential patterns of multimorbidity among older adults (≥65 years)

eTable 28. Top 10 binary and ternary sequential patterns of multimorbidity among men

eTable 29. Top 10 binary and ternary sequential patterns of multimorbidity among women

eTable 30. Prevalence of five chronic conditions: Overall (self-reported + survey-detected) and survey-detected only

eTable 31. Top 10 binary association rules of multimorbidity

eTable 32. Top 10 binary and ternary sequential patterns of multimorbidity

eFigure 1. Participant flow diagram
