# Supplementary material for: North–South Disparities in Multimorbidity Revealed by Association Rule and Sequential Pattern Mining: Evidence From the China National Health Survey (CNHS)
Source: J Epidemiol. 2026 Nov 5;36(11):354–64. doi: 10.2188/jea.JE20260009 (PMC13662270; doi:10.2188/jea.JE20260009)
Supplement: Supplementary file 1 [file je-36-354-s001.pdf]

eTable 1. Adjusted odds ratios for multimorbidity comparing northern and southern provinces

**Variable**	**Multimorbidity**		**Multimorbidity [a]**	
	**OR (95% CI)**	***P*-value**	**OR (95% CI)**	***P*-value**
**Region**				
Southern provinces	1.00 (Reference)		1.00 (Reference)	
Northern provinces	1.17 (1.10–1.25)	<0.0001	1.25 (1.17–1.35)	<0.0001

CI, confidence interval; OR, odds ratio.

Multivariable logistic regression models were used to ORs and 95% CIs. Models were adjusted for age group, sex, education attainment, marital status, residence (urban/rural), smoking status, alcohol consumption, physical activity level, and BMI group.

Multimorbidity was defined as the presence of two or more chronic conditions.

[a] Multimorbidity defined using self-reported physician-diagnosed conditions only.

eTable 2. Prevalence of multimorbidity and chronic conditions by region

**Chronic conditions**	**Total (n=19,067)**	**Southern provinces (n=9,908)**	**Northern provinces (n=9,159)**	***P*-value**
Multimorbidity	37.85 (37.16, 38.54)	34.08 (33.15, 35.03)	41.39 (40.91, 42.94)	<0.0001
Hypertension	40.05 (39.36, 40.75)	37.80 (36.84, 38.76)	42.49 (41.48, 43.51)	<0.0001
Hyperuricemia	29.06 (28.41, 29.71)	28.43 (27.54, 29.33)	29.73 (28.80, 30.68)	0.0484
Diabetes	13.42 (12.94,13.91)	12.36 (11.72, 13.03)	14.56 (13.85, 15.30)	<0.0001
CHD	7.00 (6.64, 7.37)	3.37 (3.02, 3.75)	10.92 (10.29, 11.57)	<0.0001
Anemia	6.12 (5.78, 6.46)	7.56 (7.05, 8.10)	4.55 (4.14, 5.00)	<0.0001
Spinal disease	4.54 (4.25, 4.85)	3.23 (2.89, 3.60)	5.96 (5.49, 6.47)	<0.0001
Gallbladder diseases	3.78 (3.52, 4.06)	3.83 (3.46, 4.22)	3.73 (3.36, 4.14)	0.7416
Kidney stones	3.70 (3.43, 3.98)	4.33 (3.94, 4.75)	3.01 (2.67, 3.38)	<0.0001
Stroke	3.65 (3.39, 3.93)	2.18 (1.90, 2.49)	5.24 (4.79, 5.72)	<0.0001
CLD	3.53 (3.28, 3.81)	2.88 (2.56, 3.22)	4.25 (3.84, 4.68)	<0.0001
Gout	3.32 (3.07, 3.58)	3.47 (3.12, 3.85)	3.16 (2.81, 3.53)	0.2228
Chronic gastritis	3.08 (2.84, 3.34)	3.30 (2.96, 3.67)	2.85 (2.52, 3.21)	0.0721
Arrhythmia	2.40 (2.19, 2.63)	1.76 (1.51, 2.03)	3.10 (2.76, 3.48)	<0.0001
Cancer	2.34 (2.13, 2.57)	1.69 (1.44, 1.96)	3.06 (2.71, 3.43)	<0.0001
CKD	1.80 (1.62, 2.00)	1.65 (1.40, 1.92)	1.97 (1.69, 2.27)	0.0966
Thyroid disorders	1.41 (1.24, 1.58)	1.26 (1.05, 1.50)	1.56 (1.32, 1.84)	0.0790
COPD	1.09 (0.94, 1.24)	0.97 (0.79, 1.18)	1.21 (1.00, 1.46)	0.1057
CTRD	0.86 (0.73, 1.00)	0.53 (0.40, 0.70)	1.21 (1.00, 1.46)	<0.0001
Osteoarthritis	0.74 (0.63, 0.88)	0.49 (0.37, 0.65)	1.02 (0.82, 1.24)	<0.0001
Asthma	0.70 (0.58, 0.83)	0.58 (0.44, 0.74)	0.83 (0.65, 1.04)	0.0349
Multimorbidity [a]	23.56 (22.96, 24.17)	20.02 (19.24, 20.83)	27.38 (26.47, 28.31)	<0.0001
Hypertension [a]	27.37 (26.74, 28.01)	24.89 (24.04, 25.75)	30.06 (29.12, 31.01)	<0.0001
Diabetes [a]	10.24 (9.81, 10.68)	9.17 (8.61, 9.76)	11.39 (10.74, 12.06)	<0.0001
Hyperuricemia [a]	7.90 (7.52, 8.30)	8.31 (7.77, 8.87)	7.47 (6.94, 8.03)	0.0321
CKD [a]	0.36 (0.28, 0.45)	0.20 (0.12, 0.31)	0.52 (0.39, 0.69)	0.0002
Anemia [a]	1.02 (0.88, 1.17)	1.51 (1.28, 1.77)	0.48 (0.35, 0.64)	<0.0001

CHD, coronary heart disease; CI, confidence interval; CKD, chronic kidney disease; CLD, chronic liver disease; COPD, chronic obstructive pulmonary disease; CTRD, connective tissue and rheumatic diseases.

Data are presented as prevalence (95% CI). Chronic conditions are ordered by overall prevalence from highest to lowest. *P*-values compare prevalence between regions.

[a] Estimates are based on self-reported physician diagnoses only. For multimorbidity, multimorbidity was constructed by counting chronic conditions based solely on self-reported physician diagnoses.

eTable 3. Prevalence of multimorbidity and chronic conditions among young adults (20–44 years)

**Chronic conditions**	**Total (n=4,380)**	**Southern provinces (n=2,770)**	**Northern provinces (n=1,610)**	***P*-value**
Multimorbidity	18.40 (17.26, 19.58)	16.06 (14.72, 17.49)	22.42 (20.41, 24.54)	<0.0001
Hyperuricemia	30.50 (29.14, 31.89)	28.41 (26.74, 30.13)	34.10 (31.78, 36.47)	<0.0001
Hypertension	14.93 (13.89, 16.02)	13.14 (11.9, 14.46)	18.01 (16.16, 19.98)	<0.0001
Anemia	9.57 (8.71, 10.48)	10.29 (9.18, 11.48)	8.32 (7.02, 9.78)	0.0330
Diabetes	3.88 (3.33, 4.50)	3.25 (2.62, 3.98)	4.97 (3.96, 6.15)	0.0045
Kidney stones	3.26 (2.76, 3.83)	3.61 (2.95, 4.37)	2.67 (1.94, 3.58)	0.0917
Gout	2.81 (2.34, 3.34)	2.49 (1.94, 3.14)	3.35 (2.53, 4.35)	0.0955
CLD	2.42 (1.99, 2.92)	2.02 (1.53, 2.62)	3.11 (2.31, 4.07)	0.0244
Chronic gastritis	2.21 (1.80, 2.70)	2.45 (1.91, 3.10)	1.80 (1.21, 2.58)	0.1564
Spinal disease	2.10 (1.70, 2.57)	1.81 (1.34, 2.37)	2.61 (1.89, 3.51)	0.0737
Gallbladder diseases	1.53 (1.19, 1.94)	1.62 (1.19, 2.17)	1.37 (0.86, 2.06)	0.5022
Thyroid disorders	1.28 (0.97, 1.66)	1.37 (0.97, 1.88)	1.12 (0.66, 1.76)	0.4710
Arrhythmia	1.07 (0.79, 1.42)	0.87 (0.56, 1.29)	1.43 (0.91, 2.14)	0.0817
CHD	0.82 (0.58, 1.14)	0.40 (0.20, 0.71)	1.55 (1.01, 2.28)	<0.0001
Cancer	0.62 (0.41, 0.90)	0.43 (0.22, 0.76)	0.93 (0.52, 1.53)	0.0421
Asthma	0.59 (0.39, 0.87)	0.61 (0.36, 0.98)	0.56 (0.26, 1.06)	0.8202
CTRD	0.50 (0.32, 0.76)	0.43 (0.22, 0.76)	0.62 (0.30, 1.14)	0.3964
CKD	0.34 (0.19, 0.56)	0.25 (0.10, 0.52)	0.50 (0.21, 0.98)	0.1823
COPD	0.27 (0.14, 0.48)	0.25 (0.10, 0.52)	0.31 (0.10, 0.72)	0.7240
Stroke	0.16 (0.06, 0.33)	0.11 (0.02, 0.32)	0.25 (0.07, 0.63)	0.2629
Osteoarthritis	0.09 (0.02, 0.23)	0.14 (0.04, 0.37)	0.00 (0.00, 0.00)	0.1271
Multimorbidity [a]	7.26 (6.51, 8.07)	6.25 (5.37, 7.21)	9.01 (7.65, 10.51)	0.0007
Hypertension [a]	6.69 (5.97, 7.47)	5.49 (4.67, 6.40)	8.76 (7.42, 10.25)	<0.0001
Diabetes [a]	2.15 (1.74, 2.62)	1.88 (1.41, 2.45)	2.61 (1.89, 3.51)	0.1073
Hyperuricemia [a]	6.48 (5.77, 7.25)	5.78 (4.94, 6.71)	7.70 (6.45, 9.11)	0.0126
CKD [a]	0.18 (0.08, 0.36)	0.11 (0.02, 0.32)	0.31 (0.10, 0.72)	0.1307
Anemia [a]	1.71 (1.35, 2.14)	2.17 (1.66, 2.78)	0.93 (0.52, 1.53)	0.0024

CHD, coronary heart disease; CI, confidence interval; CKD, chronic kidney disease; CLD, chronic liver disease; COPD, chronic obstructive pulmonary disease; CTRD, connective tissue and rheumatic diseases.

Data are presented as prevalence (95% CI). Chronic conditions are ordered by overall prevalence from highest to lowest. *P*-values compare prevalence between regions.

[a] Estimates are based on self-reported physician diagnoses only. For multimorbidity, multimorbidity was constructed by counting chronic conditions based solely on self-reported physician diagnoses.

eTable 4. Prevalence of multimorbidity and chronic conditions among middle-aged adults (45–64 years)

**Chronic conditions**	**Total (n=10,545)**	**Southern provinces (n=5,480)**	**Northern provinces (n=5,065)**	***P*-value**
Multimorbidity	37.34 (36.41, 38.27)	35.60 (34.33, 36.89)	39.21 (37.86, 40.57)	<0.0001
Hypertension	41.32 (40.38, 42.27)	41.97 (40.66, 43.29)	40.61 (39.26, 41.98)	0.1569
Hyperuricemia	27.95 (27.09, 28.81)	27.66 (26.48, 28.87)	28.25 (27.02, 29.51)	0.5011
Diabetes	13.74 (13.09, 14.41)	13.34 (12.45, 14.27)	14.18 (13.23, 15.17)	0.2127
CHD	5.94 (5.49, 6.40)	2.74 (2.32, 3.20)	9.40 (8.61, 10.23)	<0.0001
Anemia	4.95 (4.54, 5.38)	6.44 (5.81, 7.12)	3.34 (2.86, 3.87)	<0.0001
Spinal disease	4.90 (4.50, 5.33)	3.63 (3.15, 4.16)	6.28 (5.63, 6.98)	<0.0001
Kidney stones	3.89 (3.53, 4.27)	4.71 (4.16, 5.30)	3.00 (2.55, 3.51)	<0.0001
Gallbladder diseases	3.83 (3.47, 4.22)	4.05 (3.54, 4.61)	3.59 (3.10, 4.14)	0.2211
CLD	3.76 (3.40, 4.14)	3.18 (2.73, 3.67)	4.38 (3.84, 4.98)	0.0011
Chronic gastritis	3.33 (2.99, 3.69)	3.45 (2.98, 3.97)	3.20 (2.73, 3.72)	0.4737
Gout	3.12 (2.80, 3.47)	3.32 (2.86, 3.83)	2.90 (2.46, 3.40)	0.2164
Stroke	2.84 (2.54, 3.18)	1.68 (1.36, 2.06)	4.11 (3.58, 4.69)	<0.0001
Cancer	2.30 (2.03, 2.61)	1.46 (1.16, 1.81)	3.22 (2.75, 3.74)	<0.0001
Arrhythmia	2.02 (1.76, 2.31)	1.46 (1.16, 1.81)	2.63 (2.20, 3.10)	<0.0001
Thyroid disorders	1.54 (1.31, 1.79)	1.28 (1.00, 1.61)	1.82 (1.47, 2.22)	0.0245
CKD	1.18 (0.98, 1.40)	0.99 (0.74, 1.28)	1.38 (1.08, 1.74)	0.0591
COPD	0.91 (0.74, 1.11)	0.82 (0.60, 1.10)	1.01 (0.75, 1.32)	0.3157
CTRD	0.76 (0.60, 0.94)	0.49 (0.32, 0.72)	1.05 (0.78, 1.37)	0.0011
Osteoarthritis	0.75 (0.59, 0.93)	0.58 (0.40, 0.82)	0.93 (0.68, 1.23)	0.0407
Asthma	0.55 (0.42, 0.71)	0.40 (0.25, 0.61)	0.71 (0.50, 0.98)	0.0319
Multimorbidity [a]	22.65 (21.85, 23.46)	20.24 (19.18,21.33)	25.25 (24.06,26.47)	<0.0001
Hypertension [a]	27.84 (26.99, 28.71)	27.24 (26.07,28.44)	28.49 (27.25,29.75)	0.1541
Diabetes [a]	10.39 (9.82, 10.99)	9.76 (8.99,10.58)	11.08 (10.22,11.97)	0.0273
Hyperuricemia [a]	7.40 (6.90, 7.91)	7.96 (7.25,8.7)	6.79 (6.11,7.52)	0.0224
CKD [a]	0.37 (0.26, 0.51)	0.15 (0.06,0.29)	0.61 (0.42,0.87)	<0.0001
Anemia [a]	0.94 (0.76, 1.14)	1.41 (1.11,1.75)	0.43 (0.27,0.66)	<0.0001

CHD, coronary heart disease; CI, confidence interval; CKD, chronic kidney disease; CLD, chronic liver disease; COPD, chronic obstructive pulmonary disease; CTRD, connective tissue and rheumatic diseases.

Data are presented as prevalence (95% CI). Chronic conditions are ordered by overall prevalence from highest to lowest. *P*-values compare prevalence between regions.

[a] Estimates are based on self-reported physician diagnoses only. For multimorbidity, multimorbidity was constructed by counting chronic conditions based solely on self-reported physician diagnoses.

eTable 5. Prevalence of multimorbidity and chronic conditions among older adults (≥65 years)

**Chronic conditions**	**Total (n=4,142)**	**Southern provinces (n=1,658)**	**Northern provinces (n=2,484)**	***P*-value**
Multimorbidity	59.73 (58.22, 61.23)	59.17 (56.76, 61.55)	60.10 (58.15, 62.04)	0.5469
Hypertension	63.40 (61.91, 64.87)	65.20 (62.85, 67.49)	62.20 (60.26, 64.11)	0.0495
Hyperuricemia	30.35 (28.95, 31.77)	31.00 (28.78, 33.29)	29.91 (28.12, 31.76)	0.4548
Diabetes	22.69 (21.43, 24.00)	24.37 (22.32, 26.51)	21.58 (19.97, 23.25)	0.0358
CHD	16.22 (15.11, 17.38)	10.43 (9.00, 12.01)	20.09 (18.53, 21.72)	<0.0001
Stroke	9.39 (8.52, 10.32)	7.30 (6.09, 8.66)	10.79 (9.60, 12.08)	0.0002
Spinal disease	6.20 (5.49, 6.98)	4.28 (3.36, 5.37)	7.49 (6.48, 8.59)	<0.0001
Gallbladder diseases	6.04 (5.33, 6.80)	6.76 (5.59, 8.07)	5.56 (4.69, 6.53)	0.1122
Anemia	5.43 (4.76, 6.17)	6.69 (5.54, 8.01)	4.59 (3.80, 5.49)	0.0034
CKD	4.93 (4.29, 5.63)	6.15 (5.04, 7.42)	4.11 (3.36, 4.96)	0.0029
Arrhythmia	4.78 (4.15, 5.47)	4.22 (3.31, 5.30)	5.15 (4.32, 6.10)	0.1688
Gout	4.37 (3.77, 5.04)	5.61 (4.55, 6.83)	3.54 (2.85, 4.35)	0.0014
Cancer	4.27 (3.68, 4.93)	4.52 (3.57, 5.64)	4.11 (3.36, 4.96)	0.5154
CLD	4.15 (3.57, 4.81)	3.32 (2.51, 4.30)	4.71 (3.91, 5.62)	0.0277
Kidney stones	3.67 (3.12, 4.29)	4.28 (3.36, 5.37)	3.26 (2.60, 4.04)	0.0867
Chronic gastritis	3.38 (2.85, 3.98)	4.22 (3.31, 5.30)	2.82 (2.20, 3.55)	0.0143
COPD	2.39 (1.95, 2.90)	2.65 (1.93, 3.55)	2.21 (1.67, 2.87)	0.3641
CTRD	1.50 (1.15, 1.91)	0.84 (0.46, 1.41)	1.93 (1.43, 2.55)	0.0047
Osteoarthritis	1.42 (1.09, 1.83)	0.78 (0.42, 1.34)	1.85 (1.36, 2.46)	0.0045
Thyroid disorders	1.21 (0.90, 1.59)	1.03 (0.60, 1.64)	1.33 (0.92, 1.86)	0.3814
Asthma	1.18 (0.88, 1.56)	1.09 (0.64, 1.71)	1.25 (0.85, 1.77)	0.6359
Multimorbidity [a]	43.12 (41.60, 44.64)	42.34 (39.95, 44.76)	43.64 (41.68, 45.62)	0.4081
Hypertension [a]	48.04 (46.51, 49.58)	49.52 (47.08, 51.95)	47.06 (45.08, 49.05)	0.1211
Diabetes [a]	18.40 (17.23, 19.61)	19.42 (17.54, 21.41)	17.71 (16.23, 19.27)	0.1646
Hyperuricemia [a]	10.70 (9.77, 11.68)	13.69 (12.07, 15.44)	8.70 (7.62, 9.87)	<0.0001
CKD [a]	0.51 (0.31, 0.77)	0.54 (0.25, 1.03)	0.48 (0.25, 0.84)	0.7909
Anemia [a]	0.48 (0.30, 0.74)	0.78 (0.42, 1.34)	0.28 (0.11, 0.58)	0.0223

CHD, coronary heart disease; CI, confidence interval; CKD, chronic kidney disease; CLD, chronic liver disease; COPD, chronic obstructive pulmonary disease; CTRD, connective tissue and rheumatic diseases.

Data are presented as prevalence (95% CI). Chronic conditions are ordered by overall prevalence from highest to lowest. *P*-values compare prevalence between regions.

[a] Estimates are based on self-reported physician diagnoses only. For multimorbidity, multimorbidity was constructed by counting chronic conditions based solely on self-reported physician diagnoses.

eTable 6. Prevalence of multimorbidity and chronic conditions by sex

	Men			Women		
**Chronic conditions**	**Total (n=7,070)**	**Southern provinces (n=3,884)**	**Northern provinces (n=3,186)**	**Total (n=11,997)**	**Southern provinces (n=6,024)**	**Northern provinces (n=5,973)**
Multimorbidity	46.46 (45.30, 47.63) [a]	43.20 (41.64, 44.78) [b]	50.44 (48.69, 52.19)	32.77 (31.94, 33.62)	28.20 (27.07, 29.36) [c]	37.38 (36.16, 38.63)
Hypertension	47.33 (46.16, 48.50) [a]	45.80 (44.23, 47.39) [b]	49.18 (47.43, 50.94)	35.77 (34.91, 36.63)	32.64 (31.45, 33.84) [c]	38.93 (37.69, 40.18)
Hyperuricemia	39.11 (37.97, 40.26) [a]	39.01 (37.47, 40.56)	39.23 (37.53, 40.95)	23.13 (22.38, 23.90)	21.61 (20.58, 22.67) [c]	24.66 (23.57, 25.77)
Diabetes	16.55 (15.69, 17.44) [a]	15.83 (14.70, 17.02)	17.42 (16.12, 18.78)	11.58 (11.01, 12.16)	10.13 (9.38, 10.92) [c]	13.04 (12.20, 13.92)
CHD	7.09 (6.50, 7.71)	3.99 (3.40, 4.65) [b]	10.86 (9.80, 11.99)	6.94 (6.49, 7.41)	2.97 (2.56, 3.43) [c]	10.95 (10.17, 11.77)
Anemia	2.52 (2.17, 2.91) [a]	2.68 (2.19, 3.24)	2.32 (1.83, 2.91)	8.24 (7.75, 8.74)	10.71 (9.94, 11.52) [c]	5.74 (5.17, 6.36)
Spinal disease	4.29 (3.83, 4.78)	2.70 (2.22, 3.26) [b]	6.21 (5.40, 7.11)	4.69 (4.32, 5.09)	3.57 (3.11, 4.07) [c]	5.83 (5.25, 6.45)
Gallbladder diseases	3.32 (2.92, 3.77) [a]	3.60 (3.04, 4.24)	2.98 (2.42, 3.63)	4.05 (3.71, 4.42)	3.97 (3.49, 4.49)	4.14 (3.64, 4.67)
Kidney stones	5.81 (5.28, 6.38) [a]	6.44 (5.69, 7.25) [b]	5.05 (4.32, 5.87)	2.45 (2.18, 2.74)	2.97 (2.56, 3.43) [c]	1.93 (1.59, 2.31)
Stroke	4.43 (3.96, 4.93) [a]	2.63 (2.15, 3.18) [b]	6.62 (5.78, 7.54)	3.19 (2.89, 3.52)	1.89 (1.56, 2.27) [c]	4.50 (3.99, 5.06)
CLD	4.38 (3.92, 4.89) [a]	3.76 (3.18, 4.41) [b]	5.15 (4.41, 5.97)	3.03 (2.73, 3.36)	2.31 (1.94, 2.72) [c]	3.77 (3.30, 4.28)
Gout	7.20 (6.61, 7.83) [a]	7.29 (6.49, 8.15)	7.09 (6.23, 8.04)	1.03 (0.86, 1.23)	1.01 (0.78, 1.30)	1.05 (0.81, 1.35)
Chronic gastritis	3.00 (2.61, 3.42)	3.55 (2.99, 4.18) [b]	2.32 (1.83, 2.91)	3.13 (2.83, 3.46)	3.14 (2.71, 3.61)	3.13 (2.70, 3.60)
Arrhythmia	2.31 (1.97, 2.68)	1.85 (1.45, 2.33) [b]	2.86 (2.31, 3.50)	2.46 (2.19, 2.75)	1.69 (1.38, 2.05) [c]	3.23 (2.80, 3.71)
Cancer	1.84 (1.54, 2.18) [a]	1.54 (1.18, 1.98) [b]	2.20 (1.72, 2.77)	2.64 (2.36, 2.95)	1.78 (1.46, 2.14) [c]	3.52 (3.06, 4.01)
CKD	2.59 (2.23, 2.99) [a]	2.52 (2.05, 3.07)	2.67 (2.14, 3.29)	1.33 (1.14, 1.56)	1.08 (0.83, 1.37) [c]	1.59 (1.29, 1.94)
Thyroid disorders	0.55 (0.39, 0.75) [a]	0.57 (0.36, 0.86)	0.53 (0.31, 0.85)	1.91 (1.67, 2.17)	1.71 (1.40, 2.07)	2.11 (1.76, 2.51)
COPD	1.47 (1.20, 1.78) [a]	1.36 (1.02, 1.78)	1.60 (1.19, 2.10)	0.86 (0.70, 1.04)	0.71 (0.52, 0.96)	1.00 (0.77, 1.29)
CTRD	0.51 (0.36, 0.70) [a]	0.44 (0.26, 0.70)	0.60 (0.36, 0.93)	1.07 (0.89, 1.27)	0.60 (0.42, 0.83) [c]	1.54 (1.24, 1.89)
Osteoarthritis	0.44 (0.30, 0.62) [a]	0.31 (0.16, 0.54)	0.6 (0.36, 0.93)	0.93 (0.76, 1.11)	0.61 (0.43, 0.85) [c]	1.24 (0.97, 1.55)
Asthma	0.74 (0.55, 0.96)	0.64 (0.42, 0.95)	0.85 (0.56, 1.23)	0.68 (0.54, 0.84)	0.53 (0.36, 0.75)	0.82 (0.61, 1.08)
Multimorbidity [d]	28.98 (27.93, 30.05) [a]	26.26 (24.88, 27.68) [b]	32.30 (30.67, 33.95)	20.36 (19.65, 21.10)	16.00 (15.09, 16.95) [c]	24.76 (23.67, 25.88)
Hypertension [d]	31.80 (30.71, 32.90) [a]	30.36 (28.91, 31.83) [b]	33.55 (31.91, 35.22)	24.76 (23.99, 25.55)	21.36 (20.34, 22.42) [c]	28.19 (27.05, 29.35)
Diabetes [d]	12.14 (11.38, 12.92) [a]	11.28 (10.30, 12.31) [b]	13.18 (12.03, 14.41)	9.12 (8.61, 9.65)	7.82 (7.15, 8.53) [c]	10.43 (9.67, 11.23)
Hyperuricemia [d]	13.21 (12.43, 14.02) [a]	13.57 (12.51, 14.69) [b]	12.77 (11.63, 13.98)	4.78 (4.40, 5.17)	4.91 (4.38, 5.49)	4.64 (4.12, 5.20)
CKD [d]	0.37 (0.24, 0.54)	0.21 (0.09, 0.41) [b]	0.56 (0.34, 0.89)	0.35 (0.25, 0.47)	0.20 (0.10, 0.35) [c]	0.50 (0.34, 0.72)
Anemia [d]	0.35 (0.23, 0.52) [a]	0.49 (0.29, 0.76) [b]	0.19 (0.07, 0.41)	1.41 (1.21, 1.64)	2.17 (1.82, 2.58) [c]	0.64 (0.45, 0.87)

CHD, coronary heart disease; CI, confidence interval; CKD, chronic kidney disease; CLD, chronic liver disease; COPD, chronic obstructive pulmonary disease; CTRD, connective tissue and rheumatic diseases.

Data are presented as prevalence (95% CI). Chronic conditions are ordered by overall prevalence from highest to lowest. *P*-values compare prevalence between regions.

[a] overall comparison between male and women ($P<0.05$); [b] North–South comparison among men ($P<0.05$); [c] North–South comparison among female ($P<0.05$).

[d] Estimates are based on self-reported physician diagnoses only. For multimorbidity, multimorbidity was constructed by counting chronic conditions based solely on self-reported physician diagnoses.

eTable 7. Number of chronic conditions by region

Number of chronic conditions	Total (n=19,067)	Southern provinces (n=9,908)	Northern provinces (n=9,159)
0 chronic conditions	5,486 (28.77)	3,077 (31.06)	2,409 (26.30)
1 chronic condition	6,364 (33.38)	3,454 (34.86)	2,910 (31.77)
2 chronic conditions	4,255 (22.16)	2,098 (21.17)	2,127 (23.22)
3 chronic conditions	1,956 (10.26)	874 (8.82)	1,082 (11.81)
≥4 chronic conditions	1,036 (5.43)	405 (4.09)	631 (6.89)

eTable 8. Top 10 binary and ternary combinations of chronic conditions by region

Overall (self-reported + survey-detected)				Self-reported			
**Binary combinations**	**n_1 (%)**	**Ternary combinations**	**n_2 (%)**	**Binary combinations**	**n_1 (%)**	**Ternary combinations**	**n_2 (%)**
**Total (n1=7,217, n2=2,992)**				**Total (n_1=4,492, n_2=1,736)**			
Hypertension + Hyperuricemia	2,803 (38.84)	Hypertension + Hyperuricemia + Diabetes	589 (19.69)	Hypertension + Diabetes	1,077 (23.98)	Hypertension + Hyperuricemia + Gout	305 (17.57)
Hypertension + Diabetes	1,647 (22.82)	Hypertension + Hyperuricemia + Gout	390 (13.03)	Hypertension + CHD	724 (16.12)	Hypertension + Diabetes + CHD	200 (11.52)
Hypertension + CHD	842 (11.67)	Hypertension + Hyperuricemia + CHD	325 (10.86)	Hypertension + Hyperuricemia	716 (15.94)	Hypertension + Diabetes + Hyperuricemia	193 (11.12)
Diabetes + Hyperuricemia	836 (11.58)	Hypertension + Diabetes + CHD	250 (8.36)	Hyperuricemia + Gout	580 (12.91)	Hypertension + Diabetes + Stroke	142 (8.18)
Hyperuricemia + Gout	610 (8.45)	Hypertension + Hyperuricemia + CKD	196 (6.55)	Hypertension + Stroke	471 (10.49)	Hypertension + Hyperuricemia + CHD	125 (7.20)
Hypertension + Stroke	523 (7.25)	Hypertension + Diabetes + Stroke	171 (5.72)	Hypertension + Gout	327 (7.28)	Hypertension + Stroke + CHD	109 (6.28)
Hyperuricemia + CHD	458 (6.35)	Hypertension + Hyperuricemia + Stroke	168 (5.61)	Hypertension + Spinal disease	309 (6.88)	Diabetes + Hyperuricemia + Gout	95 (5.47)
Hypertension + Gout	401 (5.56)	Hypertension + Hyperuricemia + Kidney stones	158 (5.28)	Diabetes + CHD	287 (6.39)	Hyperuricemia + Gout + CHD	74 (4.26)
Hypertension + Spinal disease	401 (5.56)	Hypertension + Hyperuricemia + CHD	139 (4.65)	Diabetes + Hyperuricemia	286 (6.37)	Hypertension + Diabetes + Gallbladder diseases	72 (4.15)
Hypertension + Gallbladder diseases	365 (5.06)	Hypertension + Hyperuricemia + Spinal disease	136 (4.55)	Hypertension + Gallbladder diseases	279 (6.21)	Hypertension + Diabetes + Gout	71 (4.09)
**Southern provinces (n1=3,377, n2=1,279)**				**Southern provinces (n_1=1,984, n_2=712)**			
Hypertension +	326 (40.45)	Hypertension + Hyperuricemia + Diabetes	268	Hypertension + Diabetes	494 (24.90)	Hypertension + Hyperuricemia + Gout	163 (22.89)

Hyperuricemia			(20.95)				
Hyperuricemia + Gout	119 (14.76)	Hypertension + Hyperuricemia + Gout	203 (15.87)	Hypertension + Hyperuricemia	395 (19.91)	Hypertension + Diabetes + Hyperuricemia	91 (12.78)
Diabetes + Hyperuricemia	79 (9.80)	Hypertension + Hyperuricemia + CKD	90 (7.04)	Hyperuricemia + Gout	310 (15.63)	Hypertension + Diabetes + CHD	61 (8.57)
Hypertension + Diabetes	73 (9.06)	Hypertension + Hyperuricemia + CHD	89 (6.96)	Hypertension + CHD	194 (9.78)	Hypertension + Diabetes + Stroke	49 (6.88)
Hyperuricemia + Kidney stones	64 (7.94)	Hypertension + Hyperuricemia + Kidney stones	80 (6.25)	Hypertension + Gout	176 (8.87)	Hypertension + Hyperuricemia + CHD	48 (6.74)
Hypertension + Gout	54 (6.70)	Hypertension + Diabetes + CHD	76 (5.94)	Hypertension + Stroke	151 (7.61)	Diabetes + Hyperuricemia + Gout	47 (6.60)
Hyperuricemia + Anemia	52 (6.45)	Hyperuricemia + Diabetes + Gout	67 (5.24)	Hypertension + Gallbladder diseases	143 (7.21)	Hypertension + Diabetes + Gallbladder diseases	35 (4.92)
Hyperuricemia + CLD	41 (5.09)	Hypertension + Hyperuricemia + Anemia	65 (5.08)	Hypertension + Kidney stones	139 (7.01)	Hypertension + Diabetes + Gout	34 (4.78)
Hypertension + Kidney stones	37 (4.59)	Hypertension + Hyperuricemia + Gallbladder diseases	65 (5.08)	Diabetes + Hyperuricemia	136 (6.85)	Hypertension + Diabetes + Kidney stones	32 (4.49)
Hypertension + Anemia	36 (4.47)	Hypertension + Diabetes + Stroke	57 (4.46)	Hypertension + Spinal disease	99 (4.99)	Hypertension + Hyperuricemia + Kidney stones	32 (4.49)
**Northern provinces (n1=3,840, n2=1,713)**				**Northern provinces (n1=2,508, n2=1,024)**			
Hypertension + Hyperuricemia	1,573 (39.95)	Hypertension + Hyperuricemia + Diabetes	321 (18.74)	Hypertension + Diabetes	583 (23.25)	Hypertension + Hyperuricemia + Gout	163 (15.92)
Hypertension + Diabetes	882 (22.40)	Hypertension + Hyperuricemia + CHD	236 (13.78)	Hypertension + CHD	530 (21.13)	Hypertension + Diabetes + Hyperuricemia	91 (8.89)
Diabetes + Hyperuricemia	451 (11.46)	Hypertension + Hyperuricemia + Gout	187 (10.92)	Hypertension + Hyperuricemia	321 (12.80)	Hypertension + Diabetes + CHD	61 (5.96)
Hypertension + CHD	366 (9.30)	Hypertension + Diabetes + CHD	174 (10.16)	Hypertension + Stroke	320 (12.76)	Hypertension + Diabetes + Stroke	49 (4.79)

Hyperuricemia + Gout	316 (8.03)	Hypertension + Hyperuricemia + Stroke	118 (6.89)	Hyperuricemia + Gout	270 (10.77)	Hypertension + Hyperuricemia + CHD	48 (4.69)
Hypertension + Spinal disease	229 (5.82)	Hypertension + Diabetes + Stroke	114 (6.65)	Hypertension + Spinal disease	210 (8.37)	Diabetes + Hyperuricemia + Gout	47 (4.59)
Hypertension + Stroke	220 (5.59)	Hypertension + Hyperuricemia + CKD	106 (6.19)	Diabetes + CHD	199 (7.93)	Hypertension + Diabetes + Gallbladder diseases	35 (3.42)
Hypertension + Kidney stones	219 (5.56)	Hyperuricemia + Diabetes + CHD	98 (5.72)	Hypertension + CLD	157 (6.26)	Hypertension + Diabetes + Gout	34 (3.32)
Hyperuricemia + CHD	210 (5.33)	Hypertension + Hyperuricemia + Spinal disease	91 (5.31)	Hypertension + Gout	151 (6.02)	Hypertension + Diabetes + Kidney stones	32 (3.13)
Hypertension + Gout	209 (5.31)	Hypertension + Stroke + CHD	86 (5.02)	Diabetes + Hyperuricemia	150 (5.98)	Hypertension + Hyperuricemia + Kidney stones	32 (3.13)

CHD, coronary heart disease; CKD, chronic kidney disease; CLD, chronic liver disease; COPD, chronic obstructive pulmonary disease; CTRD, connective tissue and rheumatic diseases.

eTable 9. Top 10 binary and ternary combinations of chronic conditions among young adults (20–44 years)

Overall (self-reported + survey-detected)				Self-reported			
**Binary combinations**	**n_1 (%)**	**Ternary combinations**	**n_2 (%)**	**Binary combinations**	**n_1 (%)**	**Ternary combinations**	**n_2 (%)**
**Total (n_1=806, n_2=208)**				**Total (n_1 =318, n_2 =76)**			
Hypertension + Hyperuricemia	326 (40.45)	Hypertension + Hyperuricemia + Gout	53 (25.48)	Hyperuricemia + Gout	110 (34.59)	Hypertension + Hyperuricemia + Gout	28 (36.84)
Hyperuricemia + Gout	119 (14.76)	Hypertension + Hyperuricemia + Diabetes	40 (19.23)	Hypertension + Hyperuricemia	53 (16.67)	Hyperuricemia + Gout + Kidney stones	14 (18.42)
Diabetes + Hyperuricemia	79 (9.80)	Hypertension + Hyperuricemia + Kidney stones	27 (12.98)	Hypertension + Gout	31 (9.75)	Hypertension + Diabetes + Hyperuricemia	8 (10.53)
Hypertension + Diabetes	73 (9.06)	Diabetes + Hyperuricemia + Gout	16 (7.69)	Hyperuricemia + Kidney stones	25 (7.86)	Hypertension + Hyperuricemia + Kidney stones	8 (10.53)
Hyperuricemia + Kidney stones	64 (7.94)	Hyperuricemia + Gout + Kidney stones	14 (6.73)	Hypertension + Diabetes	22 (6.92)	Diabetes + Hyperuricemia + Gout	6 (7.89)
Hypertension + Gout	54 (6.70)	Hypertension + Hyperuricemia + CLD	12 (5.77)	Hypertension + Kidney stones	22 (6.92)	Hypertension + Diabetes + Gout	6 (7.89)
Hyperuricemia + Anemia	52 (6.45)	Hypertension + Diabetes + Gout	11 (5.29)	Diabetes + Hyperuricemia	16 (5.03)	Hypertension + Gout + Kidney stones	6 (7.89)
Hyperuricemia + CLD	41 (5.09)	Hypertension + Gout + Kidney stones	9 (4.33)	Hyperuricemia + CLD	15 (4.72)	Hyperuricemia + Gout + CLD	5 (6.58)
Hypertension + Kidney stones	37 (4.59)	Hypertension + Hyperuricemia + Spinal disease	9 (4.33)	Gout + Kidney stones	14 (4.40)	Hyperuricemia + Gout + Spinal disease	5 (6.58)
Hypertension + Anemia	36 (4.47)	Hypertension + Hyperuricemia + Anemia	8 (3.85)	Hypertension + CLD	14 (4.40)	Hyperuricemia + Gallbladder diseases + Kidney stones	4 (5.26)
**Southern provinces (n1=445, n2=107)**				**Southern provinces (n_1 =173, n_2 =35)**			
Hypertension + Hyperuricemia	163 (36.63)	Hypertension + Hyperuricemia + Gout	27 (25.23)	Hyperuricemia + Gout	61 (35.26)	Hypertension + Hyperuricemia + Gout	12 (34.29)
Hyperuricemia + Gout	67 (15.06)	Hypertension + Diabetes + Hyperuricemia	16 (14.95)	Hypertension + Hyperuricemia	26 (15.03)	Hyperuricemia + Gout + Kidney stones	5 (14.29)

Hyperuricemia + Kidney stones	42 (9.44)	Hypertension + Hyperuricemia + Kidney stones	15 (14.02)	Hypertension + Gout	14 (8.09)	Hyperuricemia + Gallbladder diseases + Kidney stones	4 (11.43)
Diabetes + Hyperuricemia	35 (7.87)	Diabetes + Hyperuricemia + Gout	8 (7.48)	Hypertension + Kidney stones	13 (7.51)	Hypertension + Diabetes + Gout	3 (8.57)
Hypertension + Diabetes	33 (7.42)	Hypertension + Hyperuricemia + Chronic gastritis	7 (6.54)	Hyperuricemia + Kidney stones	13 (7.51)	Hypertension + Diabetes + Hyperuricemia	3 (8.57)
Hyperuricemia + Anemia	33 (7.42)	Hypertension + Diabetes + Gout	6 (5.61)	Hyperuricemia + CLD	8 (4.62)	Hypertension + Hyperuricemia + Kidney stones	3 (8.57)
Hypertension + Gout	27 (6.07)	Hypertension + Hyperuricemia + Spinal disease	5 (4.67)	Hypertension + Diabetes	7 (4.05)	Hypertension + Hyperuricemia + Spinal disease	3 (8.57)
Hyperuricemia + Chronic gastritis	27 (6.07)	Hyperuricemia + Gout + Kidney stones	5 (4.67)	Hypertension + Spinal disease	7 (4.05)	Diabetes + Hyperuricemia + Gout	2 (5.71)
Hypertension + Kidney stones	23 (5.17)	Hyperuricemia + Gallbladder diseases + Kidney stones	5 (4.67)	Hyperuricemia + Gallbladder diseases	7 (4.05)	Hypertension + Gallbladder diseases + Kidney stones	2 (5.71)
Hypertension + Anemia	22 (4.94)	Diabetes + Hyperuricemia + Kidney stones	3 (2.80)	Hyperuricemia + Spinal disease	7 (4.05)	Hypertension + Hyperuricemia + CHD	2 (5.71)
**Northern provinces (n_1=361, n_2=101)**				**Northern provinces (n_1 =145, n_2 =41)**			
Hypertension + Hyperuricemia	163 (45.15)	Hypertension + Hyperuricemia + Gout	26 (25.74)	Hyperuricemia + Gout	49 (33.79)	Hypertension + Hyperuricemia + Gout	16 (39.02)
Hyperuricemia + Gout	52 (14.40)	Hypertension + Diabetes + Hyperuricemia	24 (23.76)	Hypertension + Hyperuricemia	27 (18.62)	Hyperuricemia + Gout + Kidney stones	9 (21.95)
Diabetes + Hyperuricemia	44 (12.19)	Hypertension + Hyperuricemia + Kidney stones	12 (11.88)	Hypertension + Gout	17 (11.72)	Hypertension + Diabetes + Hyperuricemia	5 (12.20)
Hypertension + Diabetes	40 (11.08)	Hypertension + Hyperuricemia + CLD	9 (8.91)	Hypertension + Diabetes	15 (10.34)	Hypertension + Gout + Kidney stones	5 (12.20)

Hypertension + Gout	27 (7.48)	Hyperuricemia + Gout + Kidney stones	9 (8.91)	Hyperuricemia + Kidney stones	12 (8.28)	Hypertension + Hyperuricemia + Kidney stones	5 (12.20)
Hyperuricemia + CLD	25 (6.93)	Diabetes + Hyperuricemia + Gout	8 (7.92)	Diabetes + Hyperuricemia	10 (6.90)	Diabetes + Hyperuricemia + Gout	4 (9.76)
Hyperuricemia + Kidney stones	22 (6.09)	Hypertension + Gout + Kidney stones	7 (6.93)	Gout + Kidney stones	9 (6.21)	Hyperuricemia + Gout + CLD	4 (9.76)
Hyperuricemia + Anemia	19 (5.26)	Hypertension + Diabetes + Gout	5 (4.95)	Hypertension + CLD	9 (6.21)	Hypertension + Diabetes + Gout	3 (7.32)
Hyperuricemia + Spinal disease	16 (4.43)	Hypertension + Hyperuricemia + CKD	5 (4.95)	Hypertension + Kidney stones	9 (6.21)	Hypertension + Gout + CLD	3 (7.32)
Hypertension + CLD	15 (4.16)	Hypertension + Hyperuricemia + Anemia	5 (4.95)	Hypertension + Arrhythmia	8 (5.52)	Hyperuricemia + Gout + Spinal disease	3 (7.32)

CHD, coronary heart disease; CKD, chronic kidney disease; CLD, chronic liver disease; COPD, chronic obstructive pulmonary disease; CTRD, connective tissue and rheumatic diseases.

eTable 10. Top 10 binary and ternary combinations of chronic conditions among middle-aged adults (45–64 years)

Overall (self-reported + survey-detected)				Self-reported			
**Binary combinations**	**n_1 (%)**	**Ternary combinations**	**n_2 (%)**	**Binary combinations**	**n_1 (%)**	**Ternary combinations**	**n_2 (%)**
**Total (n_1=3,937, n_2=1,521)**				**Total (n_1=2,388, n_2=859)**			
Hypertension + Hyperuricemia	1,573 (39.95)	Hypertension + Diabetes + Hyperuricemia	307 (20.18)	Hypertension + Diabetes	558 (23.37)	Hypertension + Hyperuricemia + Gout	163 (18.98)
Hypertension + Diabetes	882 (22.40)	Hypertension + Hyperuricemia + Gout	201 (13.21)	Hypertension + Hyperuricemia	380 (15.91)	Hypertension + Diabetes + Hyperuricemia	97 (11.29)
Diabetes + Hyperuricemia	451 (11.46)	Hypertension + Hyperuricemia + CHD	137 (9.01)	Hypertension + CHD	318 (13.32)	Hypertension + Diabetes + CHD	86 (10.01)
Hypertension + CHD	366 (9.30)	Hypertension + Diabetes + CHD	104 (6.84)	Hyperuricemia + Gout	301 (12.60)	Hypertension + Diabetes + Stroke	63 (7.33)
Hyperuricemia + Gout	316 (8.03)	Hypertension + Hyperuricemia + Kidney stones	93 (6.11)	Hypertension + Stroke	201 (8.42)	Diabetes + Hyperuricemia + Gout	53 (6.17)
Hypertension + Spinal disease	229 (5.82)	Hypertension + Hyperuricemia + CLD	74 (4.87)	Hypertension + Gout	176 (7.37)	Hypertension + Hyperuricemia + CHD	49 (5.70)
Hypertension + Stroke	220 (5.59)	Hypertension + Hyperuricemia + Stroke	74 (4.87)	Hypertension + Spinal disease	173 (7.24)	Hypertension + Stroke + CHD	40 (4.66)
Hypertension + Kidney stones	219 (5.56)	Hypertension + Hyperuricemia + CKD	73 (4.80)	Hypertension + Kidney stones	154 (6.45)	Hypertension + Diabetes + Gout	36 (4.19)
Hyperuricemia + CHD	210 (5.33)	Hypertension + Hyperuricemia + Spinal disease	73 (4.80)	Diabetes + Hyperuricemia	147 (6.16)	Hypertension + Diabetes + Gallbladder diseases	33 (3.84)
Hypertension + Gout	209 (5.31)	Hypertension + Diabetes + Stroke	72 (4.73)	Hypertension + Gallbladder diseases	138 (5.78)	Hypertension + Hyperuricemia + Kidney stones	33 (3.84)
**Southern provinces (n_1=1,951, n_2=677)**				**Southern provinces (n_1=1,109, n_2=363)**			
Hypertension +Hyperuricemia	804 (41.21)	Hypertension + Diabetes + Hyperuricemia	149 (22.01)	Hypertension + Diabetes	273 (24.62)	Hypertension + Hyperuricemia + Gout	88 (24.24)
Hypertension + Diabetes	453 (23.22)	Hypertension + Hyperuricemia + Gout	106 (15.66)	Hypertension + Hyperuricemia	212 (19.12)	Hypertension + Diabetes + Hyperuricemia	49 (13.50)

Diabetes + Hyperuricemia	220 (11.28)	Hypertension + Hyperuricemia + Kidney stones	48 (7.09)	Hyperuricemia + Gout	164 (14.79)	Diabetes + Hyperuricemia + Gout	28 (7.71)
Hyperuricemia + Gout	173 (8.87)	Diabetes + Hyperuricemia + Gout	38 (5.61)	Hypertension + Gout	95 (8.57)	Hypertension + Hyperuricemia + Kidney stones	21 (5.79)
Hypertension + Kidney stones	128 (6.56)	Hypertension + Hyperuricemia + Anemia	37 (5.47)	Hypertension + Kidney stones	86 (7.75)	Hypertension + Diabetes + Gallbladder diseases	20 (5.51)
Hypertension + Gout	112 (5.74)	Hypertension + Hyperuricemia + CKD	32 (4.73)	Diabetes + Hyperuricemia	77 (6.94)	Hypertension + Diabetes + Stroke	20 (5.51)
Hypertension + Gallbladder diseases	110 (5.64)	Hypertension + Hyperuricemia + CHD	29 (4.28)	Hypertension + Gallbladder diseases	74 (6.67)	Hypertension + Diabetes + CHD	19 (5.23)
Hypertension + Anemia	99 (5.07)	Hypertension + Diabetes + Gout	28 (4.14)	Hypertension + CHD	72 (6.49)	Hypertension + Diabetes + Kidney stones	19 (5.23)
Hypertension + CHD	88 (4.51)	Hypertension + Diabetes + Gallbladder diseases	28 (4.14)	Hypertension + CLD	59 (5.32)	Hyperuricemia + Gout + Kidney stones	19 (5.23)
Hypertension + Spinal disease	86 (4.41)	Hypertension + Hyperuricemia + CLD	28 (4.14)	Hypertension + Spinal disease	59 (5.32)	Hypertension + Diabetes + Gout	18 (4.96)
**Northern provinces (n_1=1,986, n_2=844)**				**Northern provinces (n_1=1,279, n_2=496)**			
Hypertension + Hyperuricemia	769 (38.72)	Hypertension + Diabetes + Hyperuricemia	158 (18.72)	Hypertension + Diabetes	285 (22.28)	Hypertension + Hyperuricemia + Gout	75 (15.12)
Hypertension + Diabetes	429 (21.60)	Hypertension + Hyperuricemia + CHD	108 (12.80)	Hypertension + CHD	246 (19.23)	Hypertension + Diabetes + CHD	67 (13.51)
Hypertension + CHD	278 (14.00)	Hypertension + Hyperuricemia + Gout	95 (11.26)	Hypertension + Hyperuricemia	168 (13.14)	Hypertension + Diabetes + Hyperuricemia	48 (9.68)
Diabetes + Hyperuricemia	231 (11.63)	Hypertension + Diabetes + CHD	78 (9.24)	Hypertension + Stroke	142 (11.10)	Hypertension + Diabetes + Stroke	43 (8.67)
Hyperuricemia + CHD	169 (8.51)	Hypertension + Hyperuricemia + Stroke	55 (6.52)	Hyperuricemia + Gout	137 (10.71)	Hypertension + Hyperuricemia + CHD	34 (6.85)

Hypertension + Stroke	150 (7.55)	Diabetes + Hyperuricemia + CHD	50 (5.92)	Hypertension + Spinal disease	114 (8.91)	Hypertension + Stroke + CHD	32 (6.45)
Hypertension + Spinal disease	143 (7.20)	Hypertension + Diabetes + Stroke	50 (5.92)	Diabetes + CHD	95 (7.43)	Diabetes + Hyperuricemia + Gout	25 (5.04)
Hyperuricemia + Gout	143 (7.20)	Hypertension + Hyperuricemia + Spinal disease	50 (5.92)	Hypertension + Gout	81 (6.33)	Hypertension + CHD + Spinal disease	25 (5.04)
Diabetes + CHD	105 (5.29)	Hypertension + Hyperuricemia + CLD	46 (5.45)	Hypertension + CLD	76 (5.94)	Hyperuricemia + Gout + CHD	25 (5.04)
Hypertension + Gout	97 (4.88)	Hypertension + Hyperuricemia + Kidney stones	45 (5.33)	Diabetes + Hyperuricemia	70 (5.47)	Diabetes + Hyperuricemia + CHD	24 (4.84)

CHD, coronary heart disease; CKD, chronic kidney disease; CLD, chronic liver disease; COPD, chronic obstructive pulmonary disease; CTRD, connective tissue and rheumatic diseases.

eTable 11. Top 10 binary and ternary combinations of chronic conditions among older adults (≥65 years)

Overall (self-reported + survey-detected)				Self-reported			
**Binary combinations**	**n_1 (%)**	**Ternary combinations**	**n_2 (%)**	**Binary combinations**	**n_1 (%)**	**Ternary combinations**	**n_2 (%)**
**Total (n_1=2,474, n_2=1,263)**				**Total (n_1=1,786, n_2=801)**			
Hypertension + Hyperuricemia	904 (36.54)	Hypertension + Diabetes + Hyperuricemia	242 (19.16)	Hypertension + Diabetes	497 (27.83)	Hypertension + Hyperuricemia + Gout	114 (14.23)
Hypertension + Diabetes	692 (27.97)	Hypertension + Hyperuricemia + CHD	183 (14.49)	Hypertension + CHD	400 (22.40)	Hypertension + Diabetes + CHD	112 (13.98)
Hypertension + CHD	469 (18.96)	Hypertension + Diabetes + CHD	143 (11.32)	Hypertension + Hyperuricemia	283 (15.85)	Hypertension + Diabetes + Hyperuricemia	88 (10.99)
Diabetes + Hyperuricemia	306 (12.37)	Hypertension + Hyperuricemia + Gout	136 (10.77)	Hypertension + Stroke	268 (15.01)	Hypertension + Diabetes + Stroke	79 (9.86)
Hypertension + Stroke	301 (12.17)	Hypertension + Hyperuricemia + CKD	116 (9.18)	Hyperuricemia + Gout	169 (9.46)	Hypertension + Hyperuricemia + CHD	73 (9.11)
Hyperuricemia + Stroke	235 (9.50)	Hypertension + Diabetes + Stroke	99 (7.84)	Diabetes + CHD	153 (8.57)	Hypertension + Stroke + CHD	69 (8.61)
Diabetes + CHD	177 (7.15)	Hypertension + Hyperuricemia + Stroke	94 (7.44)	Hypertension + Gallbladder diseases	132 (7.39)	Hyperuricemia + Gout + CHD	41 (5.12)
Hyperuricemia + Gout	175 (7.07)	Hypertension + Hyperuricemia + CHD	72 (5.70)	Hypertension + Spinal disease	126 (7.05)	Hypertension + Diabetes + Gallbladder diseases	39 (4.87)
Hypertension + Gallbladder diseases	160 (6.47)	Hypertension + Stroke + CHD	72 (5.70)	Diabetes + Hyperuricemia	123 (6.89)	Hypertension + Hyperuricemia + Stroke	38 (4.74)
Hypertension + CKD	158 (6.39)	Hypertension + Hyperuricemia + Gallbladder diseases	63 (4.99)	Hypertension + Gout	120 (6.72)	Diabetes + Hyperuricemia + Gout	36 (4.49)
**Southern provinces (n_1=981, n_2=495)**				**Southern provinces (n_1=702, n_2=314)**			
Hypertension + Hyperuricemia	372 (37.92)	Hypertension + Hyperuricemia + Diabetes	103 (20.81)	Hypertension + Diabetes	214 (30.48)	Hypertension + Hyperuricemia + Gout	63 (20.06)
Hypertension + Diabetes	302 (30.78)	Hypertension + Hyperuricemia + Gout	70 (14.14)	Hypertension + Hyperuricemia	157 (22.36)	Hypertension + Diabetes + CHD	41 (13.06)

Hypertension + CHD	132 (13.46)	Hypertension + Hyperuricemia + CHD	57 (11.52)	Hypertension + CHD	120 (17.09)	Hypertension + Diabetes + Hyperuricemia	39 (12.42)
Diabetes + Hyperuricemia	130 (13.25)	Hypertension + Hyperuricemia + CKD	56 (11.31)	Hypertension + Stroke	92 (13.11)	Hypertension + Hyperuricemia + CHD	31 (9.87)
Hypertension + Stroke	101 (10.30)	Hypertension + Diabetes + CHD	49 (9.90)	Hyperuricemia + Gout	85 (12.11)	Hypertension + Diabetes + Stroke	29 (9.24)
Hyperuricemia + Gout	89 (9.07)	Hypertension + Diabetes + Stroke	35 (7.07)	Hypertension + Gout	67 (9.54)	Hypertension + Hyperuricemia + Gallbladder diseases	21 (6.69)
Hypertension + CKD	79 (8.05)	Hypertension + Hyperuricemia + Gallbladder diseases	35 (7.07)	Hypertension + Gallbladder diseases	63 (8.97)	Hypertension + Stroke + CHD	19 (6.05)
Hypertension + Gallbladder diseases	77 (7.85)	Hypertension + Hyperuricemia + Stroke	31 (6.26)	Diabetes + Hyperuricemia	53 (7.55)	Hyperuricemia + Gout + CHD	19 (6.05)
Hypertension + Gout	72 (7.34)	Diabetes + Hyperuricemia + CHD	26 (5.25)	Diabetes + CHD	51 (7.26)	Diabetes + Hyperuricemia + Gout	17 (5.41)
Hypertension + Anemia	71 (7.24)	Hypertension + Diabetes + CKD	26 (5.25)	Hypertension + Kidney stones	40 (5.70)	Hypertension + Diabetes + Gallbladder diseases	15 (4.78)
**Northern provinces (n_1=1,493, n_2=768)**				**Northern provinces (n_1=1,084, n_2=487)**			
Hypertension + Hyperuricemia	532 (35.63)	Hypertension + Diabetes + Hyperuricemia	139 (18.10)	Hypertension + Diabetes	283 (26.11)	Hypertension + Diabetes + CHD	71 (14.58)
Hypertension + Diabetes	390 (26.12)	Hypertension + Hyperuricemia + CHD	126 (16.41)	Hypertension + CHD	280 (25.83)	Hypertension + Hyperuricemia + Gout	51 (10.47)
Hypertension + CHD	337 (22.57)	Hypertension + Diabetes + CHD	94 (12.24)	Hypertension + Stroke	176 (16.24)	Hypertension + Diabetes + Stroke	50 (10.27)
Hypertension + Stroke	200 (13.40)	Hypertension + Hyperuricemia + Gout	66 (8.59)	Hypertension + Hyperuricemia	126 (11.62)	Hypertension + Stroke + CHD	50 (10.27)
Diabetes + Hyperuricemia	176 (11.79)	Hypertension + Diabetes + Stroke	64 (8.33)	Diabetes + CHD	102 (9.41)	Hypertension + Diabetes + Hyperuricemia	49 (10.06)
Hyperuricemia + CHD	166 (11.12)	Hypertension + Hyperuricemia + Stroke	63 (8.20)	Hypertension + Spinal disease	93 (8.58)	Hypertension + Hyperuricemia + CHD	42 (8.62)

Diabetes + CHD	118 (7.90)	Hypertension + Hyperuricemia + CKD	60 (7.81)	Hyperuricemia + Gout	84 (7.75)	Hypertension + CHD + Spinal disease	30 (6.16)
Hypertension + Spinal disease	115 (7.70)	Hypertension + Stroke + CHD	53 (6.90)	Hypertension + Arrhythmia	76 (7.01)	Hypertension + CHD + Gallbladder diseases	25 (5.13)
Hypertension + CLD	86 (5.76)	Diabetes + Hyperuricemia + CHD	46 (5.99)	Hypertension + CLD	72 (6.64)	Hypertension + Diabetes + Gallbladder diseases	24 (4.93)
Hypertension + Arrhythmia	85 (5.69)	Hypertension + Hyperuricemia + Arrhythmia	41 (5.34)	Diabetes + Hyperuricemia	70 (6.46)	Hypertension + Hyperuricemia + Stroke	24 (4.93)

CHD, coronary heart disease; CKD, chronic kidney disease; CLD, chronic liver disease; COPD, chronic obstructive pulmonary disease; CTRD, connective tissue and rheumatic diseases.

eTable 12. Top 10 binary and ternary combinations of chronic conditions among men

Overall (self-reported + survey-detected)				Self-reported			
**Binary combinations**	**n_1 (%)**	**Ternary combinations**	**n_2 (%)**	**Binary combinations**	**n_1 (%)**	**Ternary combinations**	**n_2 (%)**
**Total (n_1=3,285, n_2=1,420)**				**Total (n_1=2,049, n_2=832)**			
Hypertension + Hyperuricemia	1,460 (44.44)	Hypertension + Hyperuricemia + Gout	312 (21.97)	Hyperuricemia + Gout	467 (22.79)	Hypertension + Hyperuricemia + Gout	239 (28.73)
Hypertension + Diabetes	740 (22.53)	Hypertension +Diabetes + Hyperuricemia	261 (18.38)	Hypertension + Diabetes	446 (21.77)	Hypertension + Diabetes + Hyperuricemia	94 (11.30)
Hyperuricemia + Gout	494 (15.04)	Hypertension + Hyperuricemia + CHD	149 (10.49)	Hypertension + Hyperuricemia	417 (20.35)	Hypertension + Diabetes + CHD	81 (9.74)
Diabetes + Hyperuricemia	383 (11.66)	Hypertension + Hyperuricemia + CKD	116 (8.17)	Hypertension + CHD	274 (13.37)	Diabetes + Hyperuricemia + Gout	69 (8.29)
Hypertension + CHD	330 (10.05)	Hypertension + Hyperuricemia + Kidney stones	111 (7.82)	Hypertension + Gout	257 (12.54)	Hypertension + Hyperuricemia + CHD	66 (7.93)
Hypertension + Gout	321 (9.77)	Hypertension + Diabetes + CHD	106 (7.46)	Hypertension + Stroke	210 (10.25)	Hypertension + Diabetes + Stroke	61 (7.33)
Hypertension + Stroke	240 (7.31)	Diabetes + Hyperuricemia + Gout	98 (6.90)	Hypertension + Kidney stones	161 (7.86)	Hyperuricemia + Gout + CHD	52 (6.25)
Hypertension + Kidney stones	231 (7.03)	Hypertension + Hyperuricemia + Stroke	79 (5.56)	Diabetes + Hyperuricemia	149 (7.27)	Hypertension + Diabetes + Gout	50 (6.01)
Hyperuricemia + CHD	205 (6.24)	Hypertension + Diabetes + Stroke	76 (5.35)	Diabetes + CHD	123 (6.00)	Hyperuricemia + Gout + Kidney stones	44 (5.29)
Hyperuricemia + Kidney stones	183 (5.57)	Hypertension + Diabetes + Gout	74 (5.21)	Hypertension + Spinal disease	117 (5.71)	Hypertension + Stroke + CHD	43 (5.17)
**Southern provinces (n_1=1,678, n_2=700)**				**Southern provinces (n_1=1,020, n_2=396)**			
Hypertension + Hyperuricemia	759 (45.23)	Hypertension + Hyperuricemia + Gout	167 (23.86)	Hyperuricemia + Gout	256 (25.10)	Hypertension + Hyperuricemia + Gout	130 (32.83)
Hypertension + Diabetes	402 (23.96)	Hypertension +Diabetes + Hyperuricemia	140 (20)	Hypertension + Diabetes	235 (23.04)	Hypertension + Diabetes + Hyperuricemia	45 (11.36)
Hyperuricemia + Gout	274 (16.33)	Hypertension + Hyperuricemia + CKD	61 (8.71)	Hypertension + Hyperuricemia	230 (22.55)	Diabetes + Hyperuricemia + Gout	35 (8.84)

Diabetes + Hyperuricemia	206 (12.28)	Hypertension + Hyperuricemia + Kidney stones	58 (8.29)	Hypertension + Gout	141 (13.82)	Hypertension + Diabetes + CHD	35 (8.84)
Hypertension + Gout	173 (10.31)	Diabetes + Hyperuricemia + Gout	54 (7.71)	Hypertension + Kidney stones	95 (9.31)	Hypertension + Hyperuricemia + CHD	29 (7.32)
Hypertension + Kidney stones	134 (7.99)	Hypertension + Hyperuricemia + CHD	49 (7.00)	Hypertension + CHD	91 (8.92)	Hyperuricemia + Gout + Kidney stones	26 (6.57)
Hypertension + CHD	108 (6.44)	Hypertension + Diabetes + CHD	42 (6.00)	Diabetes + Hyperuricemia	75 (7.35)	Hypertension + Diabetes + Stroke	25 (6.31)
Hyperuricemia + Kidney stones	106 (6.32)	Hypertension + Diabetes + Gout	40 (5.71)	Hypertension + Gallbladder diseases	69 (6.76)	Hypertension + Diabetes + Gout	24 (6.06)
Hypertension + Gallbladder diseases	87 (5.18)	Hypertension + Hyperuricemia + Gallbladder diseases	37 (5.29)	Hypertension + Stroke	69 (6.76)	Hypertension + Hyperuricemia + Kidney stones	23 (5.81)
Hypertension + CKD	80 (4.77)	Hyperuricemia + Gout + CKD	32 (4.57)	Hypertension + CLD	49 (4.80)	Hypertension + Diabetes + Kidney stones	21 (5.30)
**Northern provinces (n_1=1,607, n_2=720)**				**Northern provinces (n_1=1029, n_2=436)**			
Hypertension + Hyperuricemia	701 (43.62)	Hypertension + Hyperuricemia + Gout	145 (20.14)	Hypertension + Diabetes	211 (20.51)	Hypertension + Hyperuricemia + Gout	109 (25.00)
Hypertension + Diabetes	338 (21.03)	Hypertension + Diabetes + Hyperuricemia	121 (16.81)	Hyperuricemia + Gout	211 (20.51)	Hypertension + Diabetes + Hyperuricemia	49 (11.24)
Hypertension + CHD	222 (13.81)	Hypertension + Hyperuricemia + CHD	100 (13.89)	Hypertension + Hyperuricemia	187 (18.17)	Hypertension + Diabetes + CHD	46 (10.55)
Hyperuricemia + Gout	220 (13.69)	Hypertension + Diabetes + CHD	64 (8.89)	Hypertension + CHD	183 (17.78)	Hypertension + Hyperuricemia + CHD	37 (8.49)
Diabetes + Hyperuricemia	177 (11.01)	Hypertension + Hyperuricemia + CKD	55 (7.64)	Hypertension + Stroke	141 (13.70)	Hypertension + Diabetes + Stroke	36 (8.26)
Hypertension + Stroke	160 (9.96)	Hypertension + Hyperuricemia + Stroke	54 (7.50)	Hypertension + Gout	116 (11.27)	Diabetes + Hyperuricemia + Gout	34 (7.80)
Hypertension + Gout	148 (9.21)	Hypertension + Hyperuricemia + Kidney stones	53 (7.36)	Diabetes + CHD	76 (7.39)	Hyperuricemia + Gout + CHD	33 (7.57)

Hyperuricemia + CHD	142 (8.84)	Hypertension + Diabetes + Stroke	46 (6.39)	Hypertension + Spinal disease	76 (7.39)	Hypertension + Stroke + CHD	30 (6.88)
Hypertension + Spinal disease	101 (6.29)	Diabetes + Hyperuricemia + Gout	44 (6.11)	Diabetes + Hyperuricemia	74 (7.19)	Hypertension + Diabetes + Gout	26 (5.96)
Hypertension + Kidney stones	97 (6.04)	Hypertension + Hyperuricemia + CLD	42 (5.83)	Hypertension + Kidney stones	66 (6.41)	Hypertension + Gout + CHD	25 (5.73)

CHD, coronary heart disease; CKD, chronic kidney disease; CLD, chronic liver disease; COPD, chronic obstructive pulmonary disease; CTRD, connective tissue and rheumatic diseases.

eTable 13. Top 10 binary and ternary combinations of chronic conditions among women

Overall (self-reported + survey-detected)				Self-reported			
**Binary combinations**	**n_1 (%)**	**Ternary combinations**	**n_2 (%)**	**Binary combinations**	**n_1 (%)**	**Ternary combinations**	**n_2 (%)**
**Total (n_1=3,932, n_2=1,572)**				**Total (n_1=2,443, n_2=904)**			
Hypertension + Hyperuricemia	1,343 (34.16)	Hypertension + Diabetes + Hyperuricemia	328 (20.87)	Hypertension + Diabetes	631 (25.83)	Hypertension + Diabetes + CHD	119 (13.16)
Hypertension + Diabetes	907 (23.07)	Hypertension + Hyperuricemia + CHD	176 (11.20)	Hypertension + CHD	450 (18.42)	Hypertension + Diabetes + Hyperuricemia	99 (10.95)
Hypertension + CHD	512 (13.02)	Hypertension + Diabetes + CHD	144 (9.16)	Hypertension + Hyperuricemia	299 (12.24)	Hypertension + Diabetes + Stroke	81 (8.96)
Diabetes + Hyperuricemia	453 (11.52)	Hypertension + Diabetes + Stroke	95 (6.04)	Hypertension + Stroke	261 (10.68)	Hypertension + Hyperuricemia + Gout	66 (7.30)
Hypertension + Stroke	283 (7.20)	Hypertension + Hyperuricemia + Stroke	89 (5.66)	Hypertension + Spinal disease	192 (7.86)	Hypertension + Stroke + CHD	66 (7.30)
Hyperuricemia + CHD	253 (6.43)	Hypertension + Hyperuricemia + CKD	80 (5.09)	Hypertension + Gallbladder diseases	173 (7.08)	Hypertension + Hyperuricemia + CHD	59 (6.53)
Hypertension + Spinal disease	247 (6.28)	Diabetes + Hyperuricemia + CHD	78 (4.96)	Diabetes + CHD	164 (6.71)	Hypertension + Diabetes + Gallbladder diseases	53 (5.86)
Hypertension + Anemia	232 (5.90)	Hypertension + Hyperuricemia + Gout	78 (4.96)	Diabetes + Hyperuricemia	137 (5.61)	Hypertension + CHD + Spinal disease	47 (5.20)
Hypertension + Gallbladder diseases	229 (5.82)	Hypertension + Hyperuricemia + Anemia	73 (4.64)	Hypertension + CLD	136 (5.57)	Hypertension + Diabetes + Spinal disease	43 (4.76)
Diabetes + CHD	183 (4.65)	Hypertension + Hyperuricemia + Spinal disease	72 (4.58)	Hypertension + Arrhythmia	122 (4.99)	Hypertension + CHD + Gallbladder diseases	36 (3.98)
**Southern provinces (n_1=1,699, n_2=579)**				**Southern provinces (n_1=964, n_2=316)**			
Hypertension + Hyperuricemia	580 (34.14)	Hypertension + Diabetes + Hyperuricemia	128 (22.11)	Hypertension + Diabetes	259 (26.87)	Hypertension + Diabetes + Hyperuricemia	46 (14.56)

Hypertension + Diabetes	386 (22.72)	Hypertension + Hyperuricemia + Anemia	45 (7.77)	Hypertension + Hyperuricemia	165 (17.12)	Hypertension + Hyperuricemia + Gout	33 (10.44)
Diabetes + Hyperuricemia	179 (10.54)	Hypertension + Hyperuricemia + CHD	40 (6.91)	Hypertension + CHD	103 (10.68)	Hypertension + Diabetes + CHD	26 (8.23)
Hypertension + Anemia	141 (8.30)	Hypertension + Hyperuricemia + Gout	36 (6.22)	Hypertension + Stroke	82 (8.51)	Hypertension + Diabetes + Gallbladder diseases	24 (7.59)
Hypertension + CHD	115 (6.77)	Hypertension + Diabetes + CHD	34 (5.87)	Hypertension + Gallbladder diseases	74 (7.68)	Hypertension + Diabetes + Stroke	24 (7.59)
Hypertension + Gallbladder diseases	110 (6.47)	Hypertension + Diabetes + Anemia	31 (5.35)	Diabetes + Hyperuricemia	61 (6.33)	Hypertension + Hyperuricemia + CHD	19 (6.01)
Hyperuricemia + Anemia	96 (5.65)	Hypertension + Diabetes + Gallbladder diseases	30 (5.18)	Hypertension + Spinal disease	58 (6.02)	Hypertension + Stroke + CHD	14 (4.43)
Hypertension + Stroke	91 (5.36)	Hypertension + Hyperuricemia + CKD	29 (5.01)	Hyperuricemia + Gout	54 (5.60)	Diabetes + Hyperuricemia + Gout	12 (3.80)
Hypertension + Spinal disease	84 (4.94)	Hypertension + Hyperuricemia + Gallbladder diseases	28 (4.84)	Hypertension + Chronic gastritis	47 (4.88)	Hypertension + Hyperuricemia + Gallbladder diseases	12 (3.80)
Hypertension + Kidney stones	66 (3.88)	Hypertension + Diabetes + Stroke	27 (4.66)	Hypertension + Kidney stones	44 (4.56)	Hypertension + Diabetes + Arrhythmia	11 (3.48)
**Northern provinces (n_1=2,233, n_2=993)**				**Northern provinces (n_1=1,479, n_2=588)**			
Hypertension + Hyperuricemia	763 (34.17)	Hypertension + Diabetes + Hyperuricemia	200 (20.14)	Hypertension + Diabetes	372 (25.15)	Hypertension + Diabetes + CHD	93 (15.82)
Hypertension + Diabetes	521 (23.33)	Hypertension + Hyperuricemia + CHD	136 (13.7)	Hypertension + CHD	347 (23.46)	Hypertension + Diabetes + Stroke	57 (9.69)
Hypertension + CHD	397 (17.78)	Hypertension + Diabetes + CHD	110 (11.08)	Hypertension + Stroke	179 (12.10)	Hypertension + Diabetes + Hyperuricemia	53 (9.01)
Diabetes + Hyperuricemia	274 (12.27)	Hypertension +Diabetes +Stroke	68 (6.85)	Hypertension + Hyperuricemia	134 (9.06)	Hypertension + Stroke + CHD	52 (8.84)
Hyperuricemia + CHD	201 (9.00)	Hypertension + Hyperuricemia + Stroke	64 (6.45)	Hypertension + Spinal disease	134 (9.06)	Hypertension + CHD + Spinal disease	45 (7.65)

Hypertension + Stroke	192 (8.6)	Diabetes + Hyperuricemia + CHD	60 (6.04)	Diabetes + CHD	123 (8.32)	Hypertension + Hyperuricemia + CHD	40 (6.80)
Hypertension + Spinal disease	163 (7.30)	Hypertension + Stroke + CHD	54 (5.44)	Hypertension + Gallbladder diseases	99 (6.69)	Hypertension + Diabetes + Spinal disease	34 (5.78)
Diabetes + CHD	135 (6.05)	Hypertension + Hyperuricemia + CKD	51 (5.14)	Hypertension + CLD	97 (6.56)	Hypertension + Hyperuricemia + Gout	33 (5.61)
Hypertension + Gallbladder diseases	119 (5.33)	Hypertension + Hyperuricemia + Spinal disease	50 (5.04)	Hypertension + Arrhythmia	83 (5.61)	Hypertension + CHD + CLD	30 (5.10)
Hypertension + CLD	113 (5.06)	Hypertension + CHD + Spinal disease	49 (4.93)	Diabetes + Hyperuricemia	76 (5.14)	Hypertension + CHD + Gallbladder diseases	30 (5.10)

CHD, coronary heart disease; CKD, chronic kidney disease; CLD, chronic liver disease; COPD, chronic obstructive pulmonary disease; CTRD, connective tissue and rheumatic diseases.

eTable 14. Top 10 binary association rules of multimorbidity among young adults (20–44 years)

Overall (self-reported + survey-detected)					Self-reported				
**Antecedent**	**Consequent**	**Support (%)**	**Confidence (%)**	**Lift**	**Antecedent**	**Consequent**	**Support (%)**	**Confidence (%)**	**Lift**
**Total**					**Total**				
Thyroid disorders	Anemia	1.24	33.33	2.05	CHD	CLD	1.26	23.53	2.02
Gallbladder diseases	Kidney stones	0.87	20.59	1.78	CHD	Diabetes	1.26	23.53	1.92
CKD	Hypertension	1.12	81.82	1.52	Gout	Hyperuricemia	34.59	96.49	1.73
Arrhythmia	Anemia	0.99	24.24	1.49	Gallbladder diseases	Kidney stones	2.20	28.00	1.46
Gout	Hyperuricemia	14.76	99.17	1.32	Diabetes	Hypertension	6.92	56.41	1.41
Diabetes	Hypertension	9.06	58.87	1.10	CHD	Kidney stones	1.26	23.53	1.23
CKD	Hyperuricemia	1.12	81.82	1.09	Arrhythmia	Hypertension	2.83	45.00	1.13
**Southern provinces**					**Southern provinces**				
Thyroid disorders	Anemia	2.02	45.00	2.36	Arrhythmia	Anemia	1.16	28.57	8.24
Gallbladder diseases	Kidney stones	1.35	28.57	1.96	Anemia	Arrhythmia	1.16	33.33	8.24
Arrhythmia	Anemia	0.90	30.77	1.61	CHD	Diabetes	1.16	28.57	3.09
CKD	Hypertension	0.67	75.00	1.51	CHD	CLD	1.16	28.57	2.91
Hyperuricemia	Gout	15.06	20.36	1.35	CLD	Gallbladder diseases	2.31	23.53	2.54
Asthma	Anemia	0.67	25.00	1.31	Gallbladder diseases	CLD	2.31	25.00	2.54
Gallbladder diseases	Anemia	1.12	23.81	1.25	Thyroid disorders	Spinal disease	1.16	25.00	1.97
Diabetes	Hypertension	7.42	54.10	1.09	Gallbladder diseases	Kidney stones	3.47	37.50	1.71
CKD	Hyperuricemia	0.67	75.00	1.01	Gout	Hyperuricemia	35.26	96.83	1.69
**Northern provinces**					**Northern provinces**				
Gallbladder diseases	Chronic gastritis	0.83	23.08	3.47	Gallbladder diseases	Chronic gastritis	2.07	33.33	3.02
CKD	Diabetes	0.83	42.86	2.46	Stroke	Hypertension	1.38	100.00	2.16
CTRD	Diabetes	0.55	40.00	2.29	Arrhythmia	Chronic gastritis	2.07	23.08	2.09
Kidney stones	Gout	2.49	32.14	2.19	CHD	Kidney stones	2.07	30.00	1.89
Chronic gastritis	Anemia	1.66	25.00	1.96	Gout	Hyperuricemia	33.79	96.08	1.79
CKD	Hypertension	1.66	85.71	1.46	Hyperuricemia	Gout	33.79	62.82	1.79
Gout	Hypertension	14.40	98.11	1.27	Asthma	Hypertension	2.07	75.00	1.62
Asthma	Hyperuricemia	1.66	85.71	1.11	Hypertension	Diabetes	10.34	22.39	1.41
CKD	Hyperuricemia	1.66	85.71	1.11	Diabetes	Hypertension	10.34	65.22	1.41

Diabetes	Hypertension	11.08	63.49	1.08	Arrhythmia	Hypertension	5.52	61.54	1.33

CHD, coronary heart disease; CKD, chronic kidney disease; CLD, chronic liver disease; COPD, chronic obstructive pulmonary disease; CTRD, connective tissue and rheumatic diseases.

eTable 15. Top 10 binary association rules of multimorbidity among middle-aged adults (45–64 years)

Overall (self-reported + survey-detected)					Self-reported				
**Antecedent**	**Consequent**	**Support (%)**	**Confidence (%)**	**Lift**	**Antecedent**	**Consequent**	**Support (%)**	**Confidence (%)**	**Lift**
**Total**					**Total**				
CKD	Gout	0.64	21.93	2.63	Gout	Hyperuricemia	12.60	93.77	3.54
Gout	Hyperuricemia	8.03	96.34	1.76	Osteoarthritis	Spinal disease	0.59	26.42	2.09
CKD	Hyperuricemia	2.21	76.32	1.39	CKD	Hyperuricemia	0.63	48.39	1.83
CKD	Diabetes	1.02	35.09	1.19	Stroke	Hypertension	8.42	82.38	1.20
Stroke	Hypertension	5.59	80.59	1.08	CKD	Hypertension	1.01	77.42	1.12
CKD	Hypertension	2.29	78.95	1.06	Stroke	CHD	2.22	21.72	1.10
Stroke	Diabetes	2.11	30.40	1.03	Diabetes	Hypertension	23.37	72.56	1.05
Diabetes	Hypertension	22.40	75.97	1.02	Hypertension	Diabetes	23.37	33.90	1.05
**Southern provinces**					**Southern provinces**				
CKD	Gout	0.82	31.37	3.38	Gout	Hyperuricemia	14.79	93.71	3.00
CKD	Anemia	0.72	27.45	2.99	Stroke	Hypertension	5.32	77.63	1.13
Gout	Hyperuricemia	8.87	95.58	1.74	Diabetes	Hypertension	24.62	72.61	1.05
CKD	Hyperuricemia	1.95	74.51	1.36	Hypertension	Diabetes	24.62	35.69	1.05
CKD	Diabetes	0.92	35.29	1.17					
Stroke	Hypertension	3.59	81.40	1.06					
CHD	Diabetes	1.95	31.67	1.05					
CKD	Hypertension	2.05	78.43	1.02					
**Northern provinces**					**Northern provinces**				
Osteoarthritis	Spinal disease	0.50	25.64	2.17	Gout	Hyperuricemia	10.71	93.84	4.20
Gout	Hyperuricemia	7.20	97.28	1.78	CKD	Gout	0.55	28.00	2.45
CKD	Hyperuricemia	2.47	77.78	1.42	Osteoarthritis	Spinal disease	0.78	30.30	1.98
Chronic gastritis	CHD	1.46	26.13	1.28	CKD	Hyperuricemia	0.78	40.00	1.79
CKD	Diabetes	1.11	34.92	1.22	Stroke	Hypertension	11.10	84.52	1.23
Stroke	Hypertension	7.55	80.21	1.10	CKD	Hypertension	1.49	76.00	1.10
Stroke	Diabetes	2.97	31.55	1.10	Cancer	Diabetes	2.11	32.53	1.06
CKD	Hypertension	2.52	79.37	1.09	Diabetes	Hypertension	22.28	72.52	1.05
Stroke	CHD	2.01	21.39	1.05	Hypertension	Diabetes	22.28	32.35	1.05

Diabetes	Hypertension	21.60	75.26	1.04	CLD	Hyperuricemia	2.74	23.18	1.04

CHD, coronary heart disease; CKD, chronic kidney disease; CLD, chronic liver disease; COPD, chronic obstructive pulmonary disease; CTRD, connective tissue and rheumatic diseases.

eTable 16. Top 10 binary association rules of multimorbidity among older adults (≥65 years)

Overall (self-reported + survey-detected)					Self-reported				
**Antecedent**	**Consequent**	**Support (%)**	**Confidence (%)**	**Lift**	**Antecedent**	**Consequent**	**Support (%)**	**Confidence (%)**	**Lift**
**Total**					**Total**				
Gout	CKD	1.74	24.02	2.99	Gout	Hyperuricemia	9.46	95.48	4.31
CKD	Anemia	1.74	21.61	2.69	Osteoarthritis	Spinal disease	0.67	25.53	2.27
Gout	Hyperuricemia	7.07	97.77	2.15	Cancer	Diabetes	2.97	43.09	1.18
CKD	Hyperuricemia	5.86	72.86	1.60	CTRD	CHD	0.95	34.00	1.14
CTRD	CHD	0.69	30.91	1.28	Osteoarthritis	Stroke	0.56	21.28	1.13
Osteoarthritis	CHD	0.61	29.41	1.22	Osteoarthritis	CHD	0.84	31.91	1.07
Cancer	Diabetes	2.30	37.75	1.10	Kidney stones	Hyperuricemia	1.46	23.01	1.04
Stroke	CHD	3.68	25.49	1.06	Stroke	Hypertension	15.01	79.76	1.04
CLD	CHD	1.58	25.16	1.04	CKD	Hypertension	0.84	78.95	1.03
Stroke	Hypertension	12.17	84.31	1.03	Arrhythmia	Hyperuricemia	2.13	22.49	1.01
**Southern provinces**					**Southern provinces**				
CKD	Gout	2.45	24.49	2.61	Anemia	Arrhythmia	0.71	41.67	4.96
CKD	Anemia	2.14	21.43	2.19	Gout	Hyperuricemia	12.11	94.44	3.30
Gout	Hyperuricemia	9.07	96.74	2.09	CKD	Hypertension	1.14	100.00	1.26
CKD	Hyperuricemia	7.14	71.43	1.54	Cancer	Diabetes	3.42	48.00	1.21
Gout	CHD	2.04	21.74	1.34	CLD	Diabetes	2.56	43.90	1.11
Stroke	Hypertension	10.30	91.82	1.10	Stroke	Hypertension	13.11	87.62	1.11
CTRD	Hyperuricemia	0.61	50.00	1.08	Anemia	Diabetes	0.71	41.67	1.05
Cancer	Diabetes	2.65	40.00	1.06	Gout	CHD	2.85	22.22	1.03
COPD	Diabetes	1.63	39.02	1.03	CHD	Hypertension	17.09	79.47	1.01
CLD	Diabetes	1.83	38.30	1.02	Hypertension	CHD	17.09	21.58	1.01
**Northern provinces**					**Northern provinces**				
Gout	CKD	1.27	21.84	3.23	Gout	Hyperuricemia	7.75	96.55	5.37
CKD	Anemia	1.47	21.78	3.16	Kidney stones	Gallbladder diseases	1.20	21.67	2.08
Gout	Hyperuricemia	5.76	98.85	2.20	Osteoarthritis	Spinal disease	0.92	25.64	1.88
Osteoarthritis	Spinal disease	0.67	23.81	2.14	Chronic gastritis	Spinal disease	1.01	21.57	1.58
CKD	Hyperuricemia	5.02	74.26	1.65	Kidney stones	Hyperuricemia	1.29	23.33	1.30

CTRD	CHD	1.00	34.88	1.19	Arrhythmia	Hyperuricemia	2.21	21.82	1.21
Osteoarthritis	CHD	0.94	33.33	1.14	Cancer	Diabetes	2.68	39.73	1.15
Cancer	Diabetes	2.08	36.05	1.12	Chronic gastritis	Stroke	1.11	23.53	1.10
CLD	CHD	2.21	30.56	1.04	CTRD	CHD	1.38	37.50	1.06
Gallbladder diseases	CHD	2.48	30.33	1.03	Hyperuricemia	Diabetes	6.46	35.90	1.04

CHD, coronary heart disease; CKD, chronic kidney disease; CLD, chronic liver disease; COPD, chronic obstructive pulmonary disease; CTRD, connective tissue and rheumatic diseases.

eTable 17. Top 10 ternary association rules of multimorbidity

Overall (self-reported + survey-detected)					Self-reported				
**Antecedent**	**Consequent**	**Support (%)**	**Confidence (%)**	**Lift**	**Antecedent**	**Consequent**	**Support (%)**	**Confidence (%)**	**Lift**
**Total**					**Total**				
Hyperuricemia + Anemia	CKD	1.54	30.26	3.19	Hyperuricemia + CKD	Gout	0.86	75.00	3.05
Diabetes + Anemia	CKD	0.84	25.25	2.66	Hyperuricemia + Kidney stones	Gout	2.94	58.62	2.38
Diabetes + CKD	Anemia	0.84	23.81	2.46	Gout + CKD	Hyperuricemia	0.86	100.00	2.37
Hypertension + Anemia	CKD	1.54	20.44	2.15	Gout + COPD	Hyperuricemia	0.52	100.00	2.37
Hyperuricemia + CKD	Gout	2.21	28.45	1.70	Gout + Arrhythmia	Hyperuricemia	1.15	100.00	2.37
Hypertension + CTRD	CHD	0.71	41.18	1.60	Gout + Kidney stones	Hyperuricemia	2.94	98.08	2.33
Hyperuricemia + Kidney stones	Gout	1.77	25.48	1.52	Gout + Spinal disease	Hyperuricemia	1.73	96.77	2.30
Gout+ Arrhythmia	Hyperuricemia	0.67	100.00	1.52	Hypertension + Gout	Hyperuricemia	17.57	96.52	2.29
Gout + Chronic gastritis	Hyperuricemia	0.64	100.00	1.52	Gout + CLD	Hyperuricemia	1.50	96.30	2.29
Gout + Anemia	Hyperuricemia	0.97	100.00	1.52	Gout + Gallbladder diseases	Hyperuricemia	1.44	96.15	2.28
**Southern provinces**					**Southern provinces**				
Gout + Anemia	CKD	0.55	38.89	3.71	Hyperuricemia + CKD	Gout	0.84	85.71	2.75
Hyperuricemia + Anemia	CKD	1.95	28.09	2.68	Gout + CKD	Hyperuricemia	0.84	100.00	1.90
Diabetes + Anemia	CKD	1.02	22.03	2.10	Gout + Arrhythmia	Hyperuricemia	1.12	100.00	1.90
Diabetes + CKD	Anemia	1.02	25.49	1.98	Gout + CLD	Hyperuricemia	0.98	100.00	1.90
Hypertension + Anemia	CKD	2.11	20.45	1.95	Gout + Gallbladder diseases	Hyperuricemia	2.39	100.00	1.90
Hypertension + CKD	Anemia	2.11	24.11	1.87	Hyperuricemia + Kidney stones	Gout	4.07	58.00	1.86
Hyperuricemia + CKD	Anemia	1.95	23.36	1.81	Gout + Kidney stones	Hyperuricemia	4.07	96.67	1.84
Hyperuricemia + CKD	Gout	3.05	36.45	1.80	Hypertension + Gout	Hyperuricemia	22.89	96.45	1.84

Hypertension + Thyroid disorders	Gallbladder diseases	0.55	21.88	1.63	Hyperuricemia + Stroke	CHD	1.26	37.50	1.79
Hypertension + CKD	Gout	2.81	32.14	1.59	Hypertension + Hyperuricemia	Gout	22.89	55.63	1.78
**Northern provinces**					**Northern provinces**				
Hyperuricemia + Anemia	CKD	1.23	33.33	3.81	Hyperuricemia + CKD	Gout	0.88	69.23	3.46
Diabetes + Anemia	CKD	0.70	30.00	3.43	Hyperuricemia + Kidney stones	Gout	2.15	59.46	2.97
Diabetes + CKD	Anemia	0.70	22.22	3.05	Gout + CKD	Hyperuricemia	0.88	100.00	2.87
Hypertension + Anemia	CKD	1.11	20.43	2.33	Gout + Kidney stones	Hyperuricemia	2.15	100.00	2.87
CHD + Chronic gastritis	CLD	0.58	25.64	2.13	Gout + Arrhythmia	Hyperuricemia	1.17	100.00	2.87
Hypertension + Chronic gastritis	CLD	1.05	21.95	1.83	Gout + Spinal disease	Hyperuricemia	2.05	100.00	2.87
Hyperuricemia + Chronic gastritis	CLD	0.64	21.15	1.76	Diabetes + Gout	Hyperuricemia	4.69	100.00	2.87
Gout + Anemia	Hyperuricemia	0.64	100	1.56	Hypertension + Gout	Hyperuricemia	13.87	96.60	2.77
Gout + Arrhythmia	Hyperuricemia	0.70	100	1.56	Gout + CLD	Hyperuricemia	1.86	95.00	2.72
Gout + Kidney stones	Hyperuricemia	1.28	100.00	1.56	Hypertension + Hyperuricemia	Gout	13.87	54.41	2.72

CHD, coronary heart disease; CKD, chronic kidney disease; CLD, chronic liver disease; COPD, chronic obstructive pulmonary disease; CTRD, connective tissue and rheumatic diseases.

eTable 18. Top 10 ternary association rules of multimorbidity among young adults (20–44 years)

Overall (self-reported + survey-detected)					Self-reported				
**Antecedent**	**Consequent**	**Support (%)**	**Confidence (%)**	**Lift**	**Antecedent**	**Consequent**	**Support (%)**	**Confidence (%)**	**Lift**
**Total**					**Total**				
Gout + CHD	Diabetes	1.44	100.00	3.65	Hyperuricemia + Gallbladder diseases	Kidney stones	5.26	80.00	2.64
Hyperuricemia + CHD	CLD	1.44	37.50	2.89	Hypertension + Kidney stones	Gallbladder diseases	3.95	27.27	2.59
Hypertension + Arrhythmia	Anemia	0.96	33.33	2.57	Hypertension + Gallbladder diseases	Kidney stones	3.95	75.00	2.48
Hyperuricemia + Gallbladder diseases	Kidney stones	2.88	54.55	2.52	Hyperuricemia + Kidney stones	Gallbladder diseases	5.26	22.22	2.11
Hyperuricemia + Anemia	Chronic gastritis	1.92	25.00	2.36	Gout + CHD	Hypertension	3.95	100.00	1.65
Hyperuricemia + Chronic gastritis	Anemia	1.92	28.57	2.20	Diabetes + Gout	Hypertension	7.89	85.71	1.42
Diabetes + CHD	Gout	1.44	75.00	2.17	Hypertension + Hyperuricemia	Diabetes	10.53	21.62	1.26
Hyperuricemia + Gallbladder diseases	CLD	1.44	27.27	2.10	Hyperuricemia + Kidney stones	Gout	18.42	77.78	1.23
Hyperuricemia + CHD	Spinal disease	0.96	25.00	1.93	Gout + Spinal disease	Hyperuricemia	6.58	100.00	1.21
Hyperuricemia + Cancer	Kidney stones	0.96	40.00	1.85	Gout + Kidney stones	Hyperuricemia	18.42	100.00	1.21
**Southern provinces**					**Southern provinces**				
Hyperuricemia + CHD	CLD	1.87	40.00	3.29	Hypertension + Kidney stones	Spinal disease	5.71	50.00	3.50
Hypertension + Gallbladder diseases	Spinal disease	1.87	33.33	2.74	Hypertension + Gallbladder diseases	Kidney stones	5.71	100.00	3.50
Hyperuricemia + CLD	Gallbladder diseases	2.80	30.00	2.47	Hypertension + Kidney stones	Gallbladder diseases	5.71	50.00	2.92

Hyperuricemia + Gallbladder diseases	CLD	2.80	30.00	2.47	Hyperuricemia + Gallbladder diseases	Kidney stones	11.43	80.00	2.80
Hypertension + Spinal disease	Gallbladder diseases	1.87	25.00	2.06	Hyperuricemia + Kidney stones	Gallbladder diseases	11.43	44.44	2.59
Hyperuricemia + Anemia	Gallbladder diseases	1.87	25.00	2.06	Hyperuricemia + CHD	CLD	5.71	50.00	2.50
Hypertension + Cancer	Kidney stones	1.87	50.00	1.98	Hyperuricemia + CLD	CHD	5.71	28.57	2.50
Hyperuricemia + Gallbladder diseases	Kidney stones	4.67	50.00	1.98	Hypertension + Spinal disease	Kidney stones	5.71	66.67	2.33
Hypertension + Spinal disease	Anemia	1.87	25.00	1.67	Hyperuricemia + Gallbladder diseases	CLD	5.71	40.00	2.00
Hypertension + Chronic gastritis	Anemia	1.87	22.22	1.49	Hyperuricemia + Chronic gastritis	Hypertension	5.71	100.00	1.75
**Northern provinces**					**Northern provinces**				
Hyperuricemia + Chronic gastritis	Anemia	2.97	60.00	2.97	Hyperuricemia + Arrhythmia	Hypertension	4.88	100.00	1.58
Diabetes + Gout	CHD	1.98	25.00	1.98	Gout + CHD	Hypertension	4.88	100.00	1.58
Gout + Anemia	Chronic gastritis	2.97	37.50	2.97	Hypertension + CHD	Gout	4.88	100.00	1.58
Gout + CHD	Diabetes	1.98	100.00	1.98	Hyperuricemia + CLD	Gout	9.76	100.00	1.58
Hypertension + Chronic gastritis	CLD	1.98	40.00	1.98	Hyperuricemia + Kidney stones	Gout	21.95	100.00	1.58
Hypertension + Spinal disease	CLD	1.98	40.00	1.98	Gout + Spinal disease	Hyperuricemia	7.32	100.00	1.37
Diabetes + Kidney stones	Gout	1.98	100.00	1.98	Diabetes + Gout	Hyperuricemia	9.76	100.00	1.37
Hypertension + CHD	Diabetes	1.98	66.67	1.98	Hypertension + Diabetes	Hyperuricemia	12.20	100.00	1.37
Hyperuricemia + CHD	Diabetes	1.98	66.67	1.98	Gout + Kidney stones	Hyperuricemia	21.95	100.00	1.37
Diabetes + CHD	Gout	1.98	66.67	1.98	Hypertension + Hyperuricemia	Diabetes	12.20	26.32	1.35

CHD, coronary heart disease; CKD, chronic kidney disease; CLD, chronic liver disease; COPD, chronic obstructive pulmonary disease; CTRD, connective tissue and rheumatic diseases.

eTable 19. Top 10 ternary association rules of multimorbidity among middle-aged adults (45–64 years)

Overall (self-reported + survey-detected)					Self-reported				
**Antecedent**	**Consequent**	**Support (%)**	**Confidence (%)**	**Lift**	**Antecedent**	**Consequent**	**Support (%)**	**Confidence (%)**	**Lift**
**Total**					**Total**				
Diabetes + Anemia	CKD	0.66	25.64	3.94	Hyperuricemia + CKD	Gout	1.28	73.33	2.76
Hyperuricemia + Anemia	CKD	1.05	23.53	3.61	CHD + CLD	Chronic gastritis	0.93	26.67	2.31
Diabetes + CKD	Anemia	0.66	27.03	3.29	Gout + CKD	Hyperuricemia	1.28	100.00	2.30
CHD + CLD	Chronic gastritis	0.53	25.00	2.99	Gout + Arrhythmia	Hyperuricemia	1.05	100.00	2.30
Diabetes + Chronic gastritis	CLD	0.53	24.24	1.94	Gout + Gallbladder diseases	Hyperuricemia	0.70	100.00	2.30
CHD + Chronic gastritis	CLD	0.53	23.53	1.88	Gout + CLD	Hyperuricemia	1.75	100.00	2.30
Hyperuricemia + Chronic gastritis	CLD	0.85	20.97	1.68	Hyperuricemia + Kidney stones	Gout	3.26	60.87	2.29
Hyperuricemia + CKD	Gout	1.58	28.57	1.65	Gout + Kidney stones	Hyperuricemia	3.26	96.55	2.22
Diabetes + Chronic gastritis	CHD	0.72	33.33	1.53	Hypertension + Gout	Hyperuricemia	18.98	96.45	2.22
Gout + Arrhythmia	Hyperuricemia	0.59	100.00	1.48	Diabetes + Gout	Hyperuricemia	6.17	94.64	2.17
**Southern provinces**					**Southern provinces**				
Gout + Anemia	CKD	0.74	45.45	7.16	Diabetes + Arrhythmia	Gallbladder diseases	1.10	44.44	2.83
Diabetes + Anemia	CKD	1.03	25.00	3.94	Hyperuricemia + CKD	Gout	1.10	80.00	2.38
Hyperuricemia + Anemia	CKD	1.62	23.40	3.68	Hypertension + CKD	Gout	1.10	80.00	2.38
Diabetes + CKD	Anemia	1.03	41.18	3.53	Gout + CKD	Hyperuricemia	1.10	100.00	1.84
CKD + Kidney stones	Gout	0.59	66.67	3.25	Gout + Arrhythmia	Hyperuricemia	1.38	100.00	1.84
Diabetes + Arrhythmia	Gallbladder diseases	0.59	40.00	2.85	Gout + Stroke	Hyperuricemia	1.10	100.00	1.84
Gout + CKD	Anemia	0.74	31.25	2.68	Gout + CLD	Hyperuricemia	1.38	100.00	1.84
Hyperuricemia + CKD	Anemia	1.62	30.56	2.62	Gout + Gallbladder diseases	Hyperuricemia	1.38	100.00	1.84
Hypertension + CKD	Anemia	1.48	27.78	2.38	Hyperuricemia + Kidney stones	Gout	5.23	61.29	1.82

Hyperuricemia + CKD	Gout	2.36	44.44	2.16	Hypertension + Gout	Hyperuricemia	24.24	96.70	1.78
**Northern provinces**					**Northern provinces**				
Hyperuricemia + Anemia	CKD	0.59	23.81	3.59	Hyperuricemia + CKD	Gout	1.41	70.00	3.28
CHD + CLD	Chronic gastritis	0.83	25.00	3.20	Hyperuricemia + Kidney stones	Gout	1.81	60.00	2.81
CHD + Chronic gastritis	CLD	0.83	30.43	2.34	Gout + CKD	Hyperuricemia	1.41	100.00	2.80
Hyperuricemia + Anemia	Kidney stones	0.59	23.81	2.16	Gout + Kidney stones	Hyperuricemia	1.81	100.00	2.80
Diabetes + Chronic gastritis	CLD	0.59	26.32	2.02	Gout + Chronic gastritis	Hyperuricemia	1.21	100.00	2.80
Hyperuricemia + Chronic gastritis	CLD	0.83	22.58	1.73	Gout + CLD	Hyperuricemia	2.02	100.00	2.80
Hyperuricemia + Anemia	Gout	0.59	23.81	1.61	Gout + Spinal disease	Hyperuricemia	1.81	100.00	2.80
Hyperuricemia + Chronic gastritis	CHD	1.78	48.39	1.59	Diabetes + Gout	Hyperuricemia	5.04	100.00	2.80
Gout + Anemia	Hyperuricemia	0.59	100.00	1.52	Gout + CHD	Hyperuricemia	5.04	96.15	2.69
Gout + Chronic gastritis	Hyperuricemia	0.71	100.00	1.52	Hypertension + Gout	Hyperuricemia	15.12	96.15	2.69

CHD, coronary heart disease; CKD, chronic kidney disease; CLD, chronic liver disease; COPD, chronic obstructive pulmonary disease; CTRD, connective tissue and rheumatic diseases.

eTable 20. Top 10 ternary association rules of multimorbidity among older adults (≥65 years)

Overall (self-reported + survey-detected)					Self-reported				
**Antecedent**	**Consequent**	**Support (%)**	**Confidence (%)**	**Lift**	**Antecedent**	**Consequent**	**Support (%)**	**Confidence (%)**	**Lift**
**Total**					**Total**				
Diabetes + Anemia	CKD	0.66	25.64	3.94	Hyperuricemia + Cancer	Gout	1.00	61.54	3.26
Hyperuricemia + Anemia	CKD	1.05	23.53	3.61	Diabetes + Chronic gastritis	CLD	0.75	35.29	3.01
Diabetes + CKD	Anemia	0.66	27.03	3.29	Hyperuricemia + COPD	Gout	0.62	55.56	2.95
CHD + CLD	Chronic gastritis	0.53	25.00	2.99	Gout + COPD	Hyperuricemia	0.62	100.00	2.72
Diabetes + Chronic gastritis	CLD	0.53	24.24	1.94	Gout + Kidney stones	Hyperuricemia	1.12	100.00	2.72
CHD + Chronic gastritis	CLD	0.53	23.53	1.88	Gout + Chronic gastritis	Hyperuricemia	0.75	100.00	2.72
Hyperuricemia + Chronic gastritis	CLD	0.85	20.97	1.68	Gout + Cancer	Hyperuricemia	1.00	100.00	2.72
Hyperuricemia + CKD	Gout	1.58	28.57	1.65	Gout + Arrhythmia	Hyperuricemia	1.25	100.00	2.72
Diabetes + Chronic gastritis	CHD	0.72	33.33	1.53	Gout + CLD	Hyperuricemia	0.75	100.00	2.72
Gout + Arrhythmia	Hyperuricemia	0.59	100.00	1.48	Gout + Spinal disease	Hyperuricemia	1.37	100.00	2.72
**Southern provinces**					**Southern provinces**				
Gout + Anemia	CKD	0.74	45.45	7.16	Hypertension + Chronic gastritis	Arrhythmia	1.59	23.81	2.34
Diabetes + Anemia	CKD	1.03	25.00	3.94	Hyperuricemia + COPD	Gout	1.27	57.14	2.30
Hyperuricemia + Anemia	CKD	1.62	23.40	3.68	Gout + COPD	Hyperuricemia	1.27	100.00	2.18
Diabetes + CKD	Anemia	1.03	41.18	3.53	Gout + Kidney stones	Hyperuricemia	1.59	100.00	2.18
CKD + Kidney stones	Gout	0.59	66.67	3.25	Gout + Chronic gastritis	Hyperuricemia	1.59	100.00	2.18
Diabetes + Arrhythmia	Gallbladder diseases	0.59	40.00	2.85	Gout + Gallbladder diseases	Hyperuricemia	3.50	100.00	2.18
Gout + CKD	Anemia	0.74	31.25	2.68	Hypertension + Gout	Hyperuricemia	20.06	96.92	2.11
Hyperuricemia + CKD	Anemia	1.62	30.56	2.62	Gout + CHD	Hyperuricemia	6.05	95.00	2.07

Hypertension + CKD	Anemia	1.48	27.78	2.38	Diabetes + Gout	Hyperuricemia	5.41	94.44	2.06
Hyperuricemia + CKD	Gout	2.36	44.44	2.16	Hypertension + Hyperuricemia	Gout	20.06	50.81	2.05
**Northern provinces**					**Northern provinces**				
Hyperuricemia + Anemia	CKD	0.59	23.81	3.59	Hyperuricemia + Cancer	Gout	1.23	66.67	4.45
CHD + CLD	Chronic gastritis	0.83	25.00	3.20	Hyperuricemia + Chronic gastritis	CLD	0.62	60.00	4.17
CHD + Chronic gastritis	CLD	0.83	30.43	2.34	Gout + Kidney stones	Hyperuricemia	0.82	100.00	3.25
Hyperuricemia + Anemia	Kidney stones	0.59	23.81	2.16	Gout + Cancer	Hyperuricemia	1.23	100.00	3.25
Diabetes + Chronic gastritis	CLD	0.59	26.32	2.02	Gout + Arrhythmia	Hyperuricemia	1.44	100.00	3.25
Hyperuricemia + Chronic gastritis	CLD	0.83	22.58	1.73	Gout + CLD	Hyperuricemia	1.03	100.00	3.25
Hyperuricemia + Anemia	Gout	0.59	23.81	1.61	Gout + Spinal disease	Hyperuricemia	1.85	100.00	3.25
Hyperuricemia + Chronic gastritis	CHD	1.78	48.39	1.59	Gout + Stroke	Hyperuricemia	2.87	100.00	3.25
Gout + Anemia	Hyperuricemia	0.59	100.00	1.52	Diabetes + Gout	Hyperuricemia	3.90	100.00	3.25
Gout + Chronic gastritis	Hyperuricemia	0.71	100.00	1.52	Hypertension + Gout	Hyperuricemia	10.47	98.08	3.18

CHD, coronary heart disease; CKD, chronic kidney disease; CLD, chronic liver disease; COPD, chronic obstructive pulmonary disease; CTRD, connective tissue and rheumatic diseases.

eTable 21. Top 10 binary association rules of multimorbidity among men

Overall (self-reported + survey-detected)					Self-reported				
**Antecedent**	**Consequent**	**Support (%)**	**Confidence (%)**	**Lift**	**Antecedent**	**Consequent**	**Support (%)**	**Confidence (%)**	**Lift**
**Total**					**Total**				
CKD	Gout	1.61	30.46	1.98	Gout	Hyperuricemia	22.79	94.73	2.57
Hyperuricemia	Gout	15.04	24.56	1.59	CKD	Hyperuricemia	0.54	57.89	1.57
Stroke	CHD	1.77	20.28	1.49	Thyroid disorders	Hyperuricemia	0.54	50.00	1.36
Cancer	Diabetes	1.31	38.74	1.31	Cancer	Diabetes	1.81	38.54	1.26
CKD	Hyperuricemia	4.20	79.31	1.30	CKD	Hypertension	0.78	84.21	1.24
CKD	Diabetes	1.92	36.21	1.23	Stroke	Hypertension	10.25	81.40	1.20
Stroke	Hypertension	7.31	83.92	1.10	Stroke	CHD	2.83	22.48	1.16
CKD	Hypertension	4.35	82.18	1.08	Diabetes	Hypertension	21.77	71.02	1.04
CHD	Diabetes	4.32	31.70	1.07	Hypertension	Diabetes	21.77	31.97	1.04
Asthma	Hypertension	1.04	77.27	1.01	CHD	Hypertension	13.37	69.02	1.01
**Southern provinces**					**Southern provinces**				
CKD	Gout	1.97	35.48	2.12	Gout	Hyperuricemia	25.10	94.46	2.31
Hyperuricemia	Gout	16.33	26.25	1.57	Osteoarthritis	Hypertension	0.59	100.00	1.47
Cancer	Diabetes	1.37	40.35	1.31	CKD	Hypertension	0.59	100.00	1.47
CKD	Hyperuricemia	4.23	76.34	1.23	Cancer	Diabetes	1.86	40.43	1.27
CHD	Diabetes	3.04	37.23	1.21	Thyroid disorders	Hyperuricemia	0.59	50.00	1.22
Stroke	Diabetes	1.97	35.11	1.14	Stroke	Hypertension	6.76	80.23	1.18
CKD	Diabetes	1.91	34.41	1.12	CHD	Diabetes	4.61	37.30	1.17
CKD	Hypertension	4.77	86.02	1.12	Stroke	Diabetes	3.04	36.05	1.13
COPD	Diabetes	0.95	34.04	1.10	Thyroid disorders	Hypertension	0.88	75.00	1.11
Stroke	Hypertension	4.77	85.11	1.10	Diabetes	Hypertension	23.04	72.53	1.07
**Northern provinces**					**Northern provinces**				
Chronic gastritis	Spinal disease	0.75	20.34	2.02	Gout	Hyperuricemia	20.51	95.05	2.88
CKD	Gout	1.24	24.69	1.76	Chronic gastritis	CLD	1.07	22.45	2.22
Gout	Hyperuricemia	13.69	97.78	1.62	Chronic gastritis	Spinal disease	1.17	24.49	1.91
CKD	Hyperuricemia	4.17	82.72	1.37	Osteoarthritis	CHD	0.58	46.15	1.75
CKD	Diabetes	1.93	38.27	1.36	CKD	Hyperuricemia	0.68	53.85	1.63

Cancer	Diabetes	1.24	37.04	1.31	Cancer	Diabetes	1.75	36.73	1.24
Stroke	Hypertension	9.96	83.33	1.10	Stroke	Hypertension	13.70	81.98	1.20
Stroke	CHD	2.55	21.35	1.10	Hypertension	Stroke	13.70	20.06	1.20
Asthma	Hypertension	1.24	80.00	1.06	CKD	Hypertension	0.97	76.92	1.13
Chronic gastritis	CHD	0.75	20.34	1.05	Arrhythmia	Hypertension	4.86	70.42	1.03

CHD, coronary heart disease; CKD, chronic kidney disease; CLD, chronic liver disease; COPD, chronic obstructive pulmonary disease; CTRD, connective tissue and rheumatic diseases.

eTable 22. Top 10 binary association rules of multimorbidity among women

Overall (self-reported + survey-detected)					Self-reported				
**Antecedent**	**Consequent**	**Support (%)**	**Confidence (%)**	**Lift**	**Antecedent**	**Consequent**	**Support (%)**	**Confidence (%)**	**Lift**
**Total**					**Total**				
Osteoarthritis	Spinal disease	0.53	22.34	2.15	Gout	Hyperuricemia	4.63	94.96	5.16
CKD	Anemia	0.92	24.00	2.08	Osteoarthritis	Spinal disease	0.86	25.30	1.83
Gout	Hyperuricemia	2.95	95.87	2.01	CTRD	CHD	1.06	29.89	1.17
CTRD	CHD	0.66	26.26	1.48	Stroke	Hypertension	10.68	80.31	1.12
CKD	Hyperuricemia	2.62	68.67	1.44	Diabetes	Hypertension	25.83	75.84	1.06
Stroke	CHD	2.19	24.64	1.39	Hypertension	Diabetes	25.83	36.02	1.06
Gout	CHD	0.64	20.66	1.16	CKD	Hypertension	1.02	75.76	1.06
Stroke	Hypertension	7.20	81.09	1.10	Stroke	CHD	3.52	26.46	1.03
Diabetes	Hypertension	23.07	77.85	1.06	Hypertension	CHD	18.42	25.68	1.00
CKD	Diabetes	1.17	30.67	1.04	CHD	Hypertension	18.42	71.88	1.00
**Southern provinces**					**Southern provinces**				
CKD	Anemia	1.12	31.67	1.97	Gout	Hyperuricemia	5.60	94.74	3.97
Gout	Hyperuricemia	3.24	93.22	1.96	Osteoarthritis	Spinal disease	0.52	20.83	1.88
Thyroid disorders	Anemia	0.88	22.73	1.41	CKD	Hypertension	0.83	88.89	1.24
CKD	Hyperuricemia	2.35	66.67	1.40	Asthma	Diabetes	0.83	44.44	1.24
Gout	Anemia	0.71	20.34	1.27	Stroke	Hypertension	8.51	85.42	1.20
Stroke	Hypertension	5.36	88.35	1.20	Diabetes	Hypertension	26.87	74.86	1.05
Asthma	Diabetes	0.53	34.62	1.16	Hypertension	Diabetes	26.87	37.59	1.05
CKD	Diabetes	1.18	33.33	1.12	CHD	Hypertension	10.68	73.57	1.03
CHD	Diabetes	2.83	32.00	1.08	Hyperuricemia	Hypertension	17.12	71.74	1.00
CHD	Hypertension	6.77	76.67	1.04	Hypertension	Hyperuricemia	17.12	23.95	1.00
**Northern provinces**					**Northern provinces**				
Gout	Hyperuricemia	2.73	98.39	2.06	Gout	Hyperuricemia	3.99	95.16	6.40
Osteoarthritis	Spinal disease	0.72	24.24	2.04	Osteoarthritis	Spinal disease	1.08	27.12	1.74
CKD	Hyperuricemia	2.82	70.00	1.47	Stroke	Hypertension	12.10	78.17	1.09
CTRD	CHD	0.99	30.56	1.24	Diabetes	Hypertension	25.15	76.54	1.06
Gout	CHD	0.81	29.03	1.18	Hypertension	Diabetes	25.15	35.00	1.06

Stroke	CHD	3.05	27.64	1.12	Cancer	Diabetes	2.57	34.86	1.06
CLD	CHD	2.19	27.37	1.11	Hyperuricemia	Diabetes	5.14	34.55	1.05
CKD	Hypertension	3.22	80.00	1.08	CTRD	CHD	1.49	33.85	1.03
Spinal disease	CHD	3.13	26.42	1.07	Hypertension	CHD	23.46	32.64	1.03
Diabetes	Hypertension	23.33	78.94	1.07	CKD	Hypertension	1.15	70.83	1.02

CHD, coronary heart disease; CKD, chronic kidney disease; CLD, chronic liver disease; COPD, chronic obstructive pulmonary disease; CTRD, connective tissue and rheumatic diseases.

eTable 23. Top 10 ternary association rules of multimorbidity among men

Overall (self-reported + survey-detected)					Self-reported				
**Antecedent**	**Consequent**	**Support (%)**	**Confidence (%)**	**Lift**	**Antecedent**	**Consequent**	**Support (%)**	**Confidence (%)**	**Lift**
**Total**					**Total**				
Gout + Anemia	CKD	0.56	53.33	4.79	Hypertension + CKD	CHD	0.96	61.54	2.21
Hyperuricemia + Anemia	CKD	1.41	37.74	3.39	Hypertension + Asthma	Stroke	0.60	35.71	2.11
Diabetes + Anemia	CKD	0.70	27.78	2.50	Hyperuricemia + Asthma	Gout	0.60	83.33	2.09
Hypertension + Anemia	CKD	1.41	26.32	2.37	Hyperuricemia + CKD	Gout	1.08	81.82	2.05
CKD + CHD	Gout	0.63	39.13	1.40	Diabetes + Chronic gastritis	CLD	0.60	27.78	2.03
Gout + Arrhythmia	Hyperuricemia	1.13	100.00	1.39	Gout + CKD	Hyperuricemia	1.08	100.00	1.83
Gout + Chronic gastritis	Hyperuricemia	0.92	100.00	1.39	Gout + Asthma	Hyperuricemia	0.60	100.00	1.83
Gout + Anemia	Hyperuricemia	1.06	100.00	1.39	Gout + COPD	Hyperuricemia	0.72	100.00	1.83
CKD + Gallbladder diseases	Hyperuricemia	0.70	100.00	1.39	Gout + Arrhythmia	Hyperuricemia	1.92	100.00	1.83
Gout + CLD	Hyperuricemia	1.69	100.00	1.39	Gout + Gallbladder diseases	Hyperuricemia	2.16	100.00	1.83
**Southern provinces**					**Southern provinces**				
Hyperuricemia + Anemia	CKD	1.57	36.67	2.98	Hypertension + Asthma	Stroke	1.01	57.14	4.71
Diabetes + CHD	Stroke	1.43	20.41	2.34	Hyperuricemia + COPD	CHD	1.01	57.14	2.94
Diabetes + Stroke	CHD	1.43	32.26	2.28	Hyperuricemia + Arrhythmia	CLD	1.01	28.57	2.63
Hypertension + Anemia	CKD	1.71	27.91	2.27	Diabetes + COPD	CHD	1.26	50.00	2.57
Hypertension + Stroke	CHD	2.00	24.56	1.74	Hypertension + Arrhythmia	Chronic gastritis	1.01	21.05	2.32
Stroke + CHD	Diabetes	1.43	66.67	1.55	Hyperuricemia + CKD	Gout	1.01	100.00	2.22
Hyperuricemia + CKD	Gout	4.57	45.71	1.52	Hypertension + Cancer	Stroke	1.26	26.32	2.17
Gout + Chronic gastritis	Hyperuricemia	1.29	100.00	1.37	CHD + Kidney stones	Diabetes	1.77	87.50	2.07

Gout + Stroke	Hyperuricemia	1.57	100.00	1.37	Hypertension + COPD	Stroke	1.01	25.00	2.06
Gout + Gallbladder diseases	Hyperuricemia	1.86	100.00	1.37	Hyperuricemia + Stroke	CHD	1.52	40.00	2.06
**Northern provinces**					**Northern provinces**				
Hyperuricemia + Anemia	CKD	1.25	39.13	3.91	CHD + Spinal disease	Chronic gastritis	0.92	28.57	3.77
Hypertension + Anemia	CKD	1.11	24.24	2.42	Hyperuricemia + Cancer	Gout	0.69	100.00	2.83
Hypertension + Chronic gastritis	CLD	1.11	27.59	2.34	CLD + Kidney stones	Chronic gastritis	0.69	21.43	2.83
Hypertension + Gallbladder diseases	CLD	1.25	21.95	1.86	CHD + Chronic gastritis	Spinal disease	0.92	44.44	2.77
Hyperuricemia + CLD	Kidney stones	1.67	22.64	1.81	CHD + Kidney stones	Gallbladder diseases	0.69	27.27	2.70
Gout + Anemia	Hyperuricemia	1.11	100.00	1.41	Chronic gastritis + Kidney stones	CLD	0.69	42.86	2.63
Gout + Arrhythmia	Hyperuricemia	1.39	100.00	1.41	Hyperuricemia + Spinal disease	Gout	3.90	80.95	2.29
Gout + CLD	Hyperuricemia	2.36	100.00	1.41	CLD + Chronic gastritis	Kidney stones	0.69	30.00	2.26
Gout + Kidney stones	Hyperuricemia	2.50	100.00	1.41	CKD + Stroke	Hyperuricemia	0.69	100.00	2.05
Gout + Spinal disease	Hyperuricemia	2.36	100.00	1.41	Gout + CKD	Hyperuricemia	1.15	100.00	2.05

CHD, coronary heart disease; CKD, chronic kidney disease; CLD, chronic liver disease; COPD, chronic obstructive pulmonary disease; CTRD, connective tissue and rheumatic diseases.

eTable 24. Top 10 ternary association rules of multimorbidity among women

Overall (self-reported + survey-detected)					Self-reported				
**Antecedent**	**Consequent**	**Support (%)**	**Confidence (%)**	**Lift**	**Antecedent**	**Consequent**	**Support (%)**	**Confidence (%)**	**Lift**
**Total**					**Total**				
Hyperuricemia + Anemia	CKD	1.65	26.26	3.28	Hyperuricemia + CKD	Gout	0.66	66.67	6.34
Diabetes + Anemia	CKD	0.95	23.81	2.97	Gout + CKD	Hyperuricemia	0.66	100.00	3.28
Diabetes + CKD	Anemia	0.95	34.09	2.75	Gout + Kidney stones	Hyperuricemia	0.77	100.00	3.28
Diabetes + Chronic gastritis	CLD	0.51	27.59	2.46	Gout + Chronic gastritis	Hyperuricemia	0.66	100.00	3.28
Hyperuricemia + CKD	Anemia	1.65	26.26	2.12	Gout + Spinal disease	Hyperuricemia	0.77	100.00	3.28
Hypertension + CKD	Anemia	1.65	25.24	2.03	Hyperuricemia + Chronic gastritis	Gout	0.66	33.33	3.17
Hyperuricemia + Chronic gastritis	CLD	0.76	22.64	2.02	Diabetes + Gout	Hyperuricemia	2.88	96.30	3.15
Gout + CKD	Hyperuricemia	0.95	100.00	1.66	Hypertension + Gout	Hyperuricemia	7.30	95.65	3.13
Gout + Anemia	Hyperuricemia	0.89	100.00	1.66	Gout + Stroke	Hyperuricemia	1.11	90.91	2.98
Gout + Stroke	Hyperuricemia	0.70	100.00	1.66	Hypertension + Hyperuricemia	Gout	7.30	30.56	2.91
**Southern provinces**					**Southern provinces**				
Hyperuricemia + Anemia	CKD	2.42	23.73	2.86	Hyperuricemia + Chronic gastritis	Gout	0.95	42.86	3.08
Diabetes + Gout	Anemia	1.04	42.86	2.28	Hypertension + Anemia	Arrhythmia	0.95	27.27	2.69
Hypertension + CKD	Anemia	2.59	40.54	2.15	Stroke + Chronic gastritis	CHD	1.27	57.14	2.51
Hyperuricemia + CKD	Anemia	2.42	37.84	2.01	Gout + Kidney stones	Hyperuricemia	0.95	100.00	2.39
Diabetes + CKD	Anemia	1.21	36.84	1.96	Gout + Spinal disease	Hyperuricemia	0.95	100.00	2.39
Gout + CKD	Hyperuricemia	1.21	100.00	1.61	Gout + Chronic gastritis	Hyperuricemia	0.95	100.00	2.39
Gout + CHD	Hyperuricemia	1.21	100.00	1.61	Gout + Gallbladder diseases	Hyperuricemia	1.27	100.00	2.39
Gout + Anemia	Hyperuricemia	1.90	100.00	1.61	Gout + CHD	Hyperuricemia	2.22	100.00	2.39

Hypertension + Gout	Hyperuricemia	6.22	97.30	1.56	CHD + Chronic gastritis	Stroke	1.27	40.00	2.38
Diabetes + Gout	Hyperuricemia	2.25	92.86	1.49	Hypertension + Gout	Hyperuricemia	10.44	97.06	2.32
**Northern provinces**					**Northern provinces**				
Hyperuricemia + Anemia	CKD	1.21	30.00	3.82	Diabetes + Gout	Hyperuricemia	2.38	100.00	4.08
Hypertension + Chronic gastritis	Gallbladder diseases	1.21	22.64	1.83	Hypertension + Gout	Hyperuricemia	5.61	94.29	3.85
Gout + Stroke	Hyperuricemia	1.01	100.00	1.69	Hyperuricemia + Stroke	Gout	1.53	33.33	3.84
Diabetes + Gout	Hyperuricemia	1.71	100.00	1.69	Gout + Stroke	Hyperuricemia	1.53	90.00	3.68
Hypertension + Gout	Hyperuricemia	4.23	100.00	1.69	Hypertension + Hyperuricemia	Gout	5.61	31.43	3.62
Gout + CHD	Hyperuricemia	1.71	94.44	1.60	Gout + CHD	Hyperuricemia	2.55	83.33	3.40
Diabetes + CKD	Hyperuricemia	2.01	80.00	1.35	Hyperuricemia + CHD	Gout	2.55	28.85	3.33
Hypertension + Gout	Stroke	1.01	23.81	1.34	Diabetes + Hyperuricemia	Gout	2.38	21.54	2.48
Hypertension + CKD	Hyperuricemia	5.14	77.27	1.31	Diabetes + Kidney stones	Hyperuricemia	1.19	58.33	2.38
Diabetes + Gallbladder diseases	Stroke	1.01	22.73	1.28	Diabetes + Chronic gastritis	CLD	1.02	31.58	1.88

CHD, coronary heart disease; CKD, chronic kidney disease; CLD, chronic liver disease; COPD, chronic obstructive pulmonary disease; CTRD, connective tissue and rheumatic diseases.

eTable 25. Top 10 binary and ternary sequential patterns of multimorbidity among young adults (20–44 years)

Overall (self-reported + survey-detected)				Self-reported			
**Binary sequences**	**Support (%)**	**Ternary sequences**	**Support (%)**	**Binary sequences**	**Support (%)**	**Ternary sequences**	**Support (%)**
**Total**				**Total**			
Hypertension → Hyperuricemia	14.29	Hyperuricemia → Kidney stones → Hypertension	2.06	Hypertension → Hyperuricemia	6.35	Hyperuricemia → Hypertension → Diabetes	2.90
Hyperuricemia → Hypertension	6.38	Kidney stones → Hypertension → Hyperuricemia	2.06	Hyperuricemia → Hypertension	5.69	Gout → Hypertension → Diabetes	2.90
Kidney stones → Hyperuricemia	6.12	Chronic gastritis → Hypertension → Hyperuricemia	1.55	Gout → Hypertension	3.68	Hypertension → Hyperuricemia → Thyroid disorders	1.45
CLD → Hyperuricemia	4.34	Spinal disease → Hypertension → Hyperuricemia	1.55	Hypertension → Gout	3.68	Hypertension → Gout → Thyroid disorders	1.45
Gout → Hypertension	4.21	Hypertension → Kidney stones → Hyperuricemia	1.55	Kidney stones → Hyperuricemia	3.34	Chronic gastritis → Spinal disease → Kidney stones	1.45
Chronic gastritis → Hyperuricemia	4.08	CLD → Hypertension → Hyperuricemia	1.55	Kidney stones → Hypertension	3.34	Spinal disease → Hypertension → Kidney stones	1.45
Spinal disease → Hyperuricemia	3.32	CLD → Spinal disease → Hypertension	1.03	CLD → Hyperuricemia	2.68	Hypertension → Hyperuricemia → Kidney stones	1.45
Kidney stones → Hyperuricemia	3.19	Kidney stones → Hyperuricemia → Hypertension	1.03	Chronic gastritis → Hypertension	2.68	Hypertension → Gout → Kidney stones	1.45
Diabetes → Hyperuricemia	3.06	Kidney stones → Gout → Hypertension	1.03	Hypertension → Kidney stones	2.34	Gallbladder diseases → Diabetes → Kidney stones	1.45
Hypertension → Diabetes	3.06	Gout → Kidney stones → Hypertension	1.03	Hyperuricemia → Kidney stones	2.34	CLD → Diabetes → Kidney stones	1.45
**Southern provinces**				**Southern provinces**			
Hypertension → Hyperuricemia	13.19	Hyperuricemia → Kidney stones → Hypertension	3.09	Hypertension → Hyperuricemia	8.07	Hypertension → Hyperuricemia → Thyroid disorders	3.33
Kidney stones → Hyperuricemia	7.41	Kidney stones → Hypertension → Hyperuricemia	3.09	Hypertension → Gout	4.97	Hypertension → Gout → Thyroid disorders	3.33
Chronic gastritis → Hyperuricemia	5.56	Chronic gastritis → Hypertension → Hyperuricemia	2.06	CLD → Hyperuricemia	4.35	Spinal disease → Hypertension → Kidney stones	3.33
Hyperuricemia → Hypertension	4.86	Spinal disease → Hypertension → Hyperuricemia	2.06	Gallbladder diseases → Hyperuricemia	3.73	Gallbladder diseases → Diabetes → Kidney stones	3.33
Kidney stones → Hypertension	3.47	Hypertension → Kidney stones → Hyperuricemia	2.06	Hypertension → Kidney stones	3.11	CLD → Diabetes → Kidney stones	3.33
CLD → Hyperuricemia	3.47	CLD → Hypertension → Hyperuricemia	2.06	Chronic gastritis → Hyperuricemia	3.11	Spinal disease → Hypertension → Kidney stones	3.33
Gout → Hypertension	3.24	CLD → Gallbladder diseases → Hyperuricemia	2.06	Kidney stones → Hypertension	3.11	Gallbladder diseases → Diabetes → Kidney	3.33

						stones	
Diabetes → Hyperuricemia	3.24	Hypertension → Hyperuricemia → Diabetes	2.06	Kidney stones → Hyperuricemia	2.48	CLD → Diabetes → Kidney stones	3.33
Spinal disease → Hyperuricemia	3.24	Hypertension → Spinal disease → Anemia	2.06	Hyperuricemia → Hypertension	2.48	Chronic gastritis → Hypertension → Hyperuricemia	3.33
Chronic gastritis → Hypertension	2.31	Gallbladder diseases → Hyperuricemia → Anemia	2.06	Chronic gastritis → Hypertension	2.48	Spinal disease → Hypertension → Hyperuricemia	3.33
**Northern provinces**				**Northern provinces**			
Hypertension →Hyperuricemia	15.63	Hyperuricemia →Hypertension → Diabetes	2.06	Hyperuricemia → Hypertension	9.42	Hyperuricemia → Hypertension → Diabetes	5.13
Hyperuricemia → Hypertension	8.24	CLD→ Spinal disease → Hypertension	2.06	Gout → Hypertension	6.52	Chronic gastritis → Spinal disease → Kidney stones	2.56
Gout → Gout	5.40	Kidney stones → Hyperuricemia → Hypertension	2.06	Hypertension → Hyperuricemia	4.35	Hypertension → Hyperuricemia → Kidney stones	2.56
CLD → Hyperuricemia	5.40	Kidney stones → Gout → Hypertension	2.06	Kidney stones → Hyperuricemia	4.35	Hypertension → Gout → Kidney stones	2.56
Kidney stones → Hyperuricemia	4.55	CLD → Kidney stones → Hypertension	2.06	Hypertension → Diabetes	4.35	CHD → CLD → Kidney stones	2.56
Hypertension → Diabetes	3.98	Spinal disease → Kidney stones → Hyperuricemia	2.06	Kidney stones → Hypertension	3.62	Kidney stones → Spinal disease → Hyperuricemia	2.56
Spinal disease → Hyperuricemia	3.41	CLD → Spinal disease → Hyperuricemia	2.06	Kidney stones → Gout	3.62	Kidney stones → Hypertension → Hyperuricemia	2.56
Kidney stones → Hypertension	2.84	Hypertension → CLD → Hyperuricemia	2.06	Hyperuricemia → Spinal disease	2.90	Hypertension → asthma → Hyperuricemia	2.56
Diabetes → Hyperuricemia	2.84	Chronic gastritis → Spinal disease → Kidney stones	1.03	Hyperuricemia → Kidney stones	2.90	Hypertension → Arrhythmia → Hyperuricemia	2.56
CLD → Hypertension	2.27	Hypertension → Hyperuricemia → Kidney stones	1.03	Chronic gastritis → Hypertension	2.90	Kidney stones → Spinal disease → Gout	2.56

CHD, coronary heart disease; CKD, chronic kidney disease; CLD, chronic liver disease; COPD, chronic obstructive pulmonary disease; CTRD, connective tissue and rheumatic diseases.

eTable 26. Top 10 binary and ternary sequential patterns of multimorbidity among middle-aged adults (45–64 years)

Overall (self-reported + survey-detected)				Self-reported			
**Binary sequences**	**Support (%)**	**Ternary sequences**	**Support (%)**	**Binary sequences**	**Support (%)**	**Ternary sequences**	**Support (%)**
**Total**				**Total**			
Hypertension →Hyperuricemia	25.31	Hypertension → Diabetes → Hyperuricemia	5.39	Hypertension → Diabetes	11.36	Hypertension → Diabetes → CHD	2.70
Hypertension →Diabetes	10.07	Hypertension → CHD → Hyperuricemia	3.34	Hypertension → Hyperuricemia	10.70	Hypertension → Diabetes → Hyperuricemia	1.93
Diabetes → Hypertension	6.70	Diabetes → Hypertension → Hyperuricemia	2.70	Hypertension → CHD	7.22	Hypertension → Diabetes → Stroke	1.67
Diabetes → Hyperuricemia	6.65	Hypertension → Stroke → Hyperuricemia	2.27	Diabetes → Hypertension	6.82	Diabetes → Hypertension → CHD	1.54
CHD → Hyperuricemia	4.42	CLD → Hypertension → Hyperuricemia	2.20	Hypertension → Stroke	5.08	Diabetes → Hypertension → Hyperuricemia	1.41
Hypertension → CHD	4.26	CHD → Hypertension → Hyperuricemia	1.99	Hypertension → Gout	4.37	Hypertension → Kidney stones → Hyperuricemia	1.29
Hyperuricemia → Hypertension	3.99	Kidney stones → Hypertension → Hyperuricemia	1.77	CHD → Hypertension	4.14	Hypertension → CHD → Hyperuricemia	1.29
Spinal disease → Hypertension	3.81	Hypertension → Kidney stones → Hyperuricemia	1.70	Spinal disease → Hypertension	4.10	Hypertension → Hyperuricemia → Diabetes	1.16
CHD → Hypertension	3.68	Spinal disease → Hypertension → Hyperuricemia	1.56	CLD → Hypertension	3.39	Hypertension → CHD → Diabetes	1.16
Kidney stones → Hypertension	3.44	Hypertension → Diabetes → CHD	1.49	Diabetes → Hyperuricemia	3.07	CLD → Hypertension → Hyperuricemia	1.03
**Southern provinces**				**Southern provinces**			
Hypertension → Hyperuricemia	24.46	Hypertension → Diabetes → Hyperuricemia	4.15	Hypertension → Hyperuricemia	12.60	Hypertension → Kidney stones → Hyperuricemia	2.11
Hypertension → Diabetes	10.13	Diabetes → Hypertension → Hyperuricemia	2.71	Hypertension → Diabetes	11.63	Hypertension → Diabetes → Hyperuricemia	1.81
Diabetes → Hypertension	6.53	Hypertension → Kidney stones → Hyperuricemia	2.07	Diabetes → Hypertension	6.54	Hypertension → Diabetes → Kidney stones	1.51
Diabetes → Hyperuricemia	5.68	CLD → Hypertension → Hyperuricemia	1.59	Hypertension → Gout	5.38	Diabetes → Hypertension → Hyperuricemia	1.51
Hyperuricemia → Hypertension	4.62	Kidney stones → Hypertension → Hyperuricemia	1.44	Hypertension → Kidney stones	3.94	Hypertension → Kidney stones → Gout	1.51
Kidney stones → Hypertension	4.03	Hypertension → Gallbladder diseases → Hyperuricemia	1.28	Hypertension → Stroke	3.75	Hypertension → Diabetes → Stroke	1.20
Kidney stones → Hyperuricemia	3.77	Hypertension → CHD → Hyperuricemia	1.28	Hyperuricemia → Hypertension	3.75	Hypertension → Hyperuricemia → Diabetes	1.20
Gallbladder diseases → Hypertension	3.18	Hypertension → Stroke → Hyperuricemia	1.12	Hypertension → CHD	3.75	Hypertension → Diabetes → CHD	1.20

CLD → Hypertension	2.97	Chronic gastritis → Hypertension → Hyperuricemia	1.12	CLD → Hypertension	3.46	Hypertension → Hyperuricemia → Spinal disease	0.90
Spinal disease → Hypertension	2.97	Spinal disease → Hypertension → Hyperuricemia	1.12	Kidney stones → Hypertension	3.37	Kidney stones → Hypertension → Hyperuricemia	0.90
**Northern provinces**				**Northern provinces**			
Hypertension → Hyperuricemia	26.15	Hypertension → Diabetes → Hyperuricemia	6.39	Hypertension → Diabetes	11.13	Hypertension → Diabetes → CHD	3.81
Hypertension → Diabetes	10.00	Hypertension → CHD → Hyperuricemia	4.99	Hypertension → CHD	10.22	Diabetes → Hypertension → CHD	2.24
Diabetes → Hyperuricemia	7.60	Hypertension → Stroke → Hyperuricemia	3.20	Hypertension → Hyperuricemia	9.05	Hypertension → Diabetes → Stroke	2.02
CHD→ Hyperuricemia	7.29	CHD → Hypertension → Hyperuricemia	3.07	Diabetes → Hypertension	7.06	Hypertension → Diabetes → Hyperuricemia	2.02
Diabetes → Hypertension	6.88	Diabetes → Hypertension → Hyperuricemia	2.69	Hypertension → Stroke	6.23	Hypertension → CHD → Stroke	1.57
Hypertension → CHD	6.41	CLD → Hypertension → Hyperuricemia	2.69	CHD → Hypertension	6.15	Hypertension → CHD → Hyperuricemia	1.57
CHD → Hypertension	5.52	Hypertension → Diabetes → CHD	2.17	Spinal disease → Hypertension	5.23	CLD → Hypertension → Hyperuricemia	1.57
Spinal disease → Hypertension	4.64	Kidney stones → Hypertension → Hyperuricemia	2.05	Diabetes → CHD	3.82	Hypertension → CHD → Diabetes	1.57
Spinal disease → Hyperuricemia	3.96	Spinal disease→ Hypertension → Hyperuricemia	1.92	Hypertension → Gout	3.49	Diabetes → Hypertension → Hyperuricemia	1.35
Hypertension → Stroke	3.91	Hypertension → Spinal disease → Hyperuricemia	1.66	CLD → Hypertension	3.32	Spinal disease → CHD → Hypertension	1.35

CHD, coronary heart disease; CKD, chronic kidney disease; CLD, chronic liver disease; COPD, chronic obstructive pulmonary disease; CTRD, connective tissue and rheumatic diseases.

eTable 27. Top 10 binary and ternary sequential patterns of multimorbidity among older adults (≥65 years)

Overall (self-reported + survey-detected)				Self-reported			
**Binary sequences**	**Support (%)**	**Ternary sequences**	**Support (%)**	**Binary sequences**	**Support (%)**	**Ternary sequences**	**Support (%)**
**Total**				**Total**			
Hypertension → Hyperuricemia	25.74	Hypertension → Diabetes → Hyperuricemia	5.94	Hypertension → Diabetes	15.51	Hypertension → CHD → Diabetes	3.43
Hypertension → Diabetes	14.08	Hypertension → CHD → Hyperuricemia	5.85	Hypertension → CHD	13.08	Hypertension → CHD → Hyperuricemia	3.30
Hypertension → CHD	9.24	Hypertension → Stroke → Hyperuricemia	2.97	Hypertension → Hyperuricemia	11.55	Hypertension → Diabetes → CHD	2.61
Diabetes → Hyperuricemia	8.15	Hypertension → CHD → Diabetes	2.88	Hypertension → Stroke	9.24	Hypertension → Diabetes → Hyperuricemia	2.47
CHD → Hyperuricemia	7.77	CHD → Hypertension → Hyperuricemia	2.80	Diabetes → Hypertension	6.34	Hypertension → Stroke → Diabetes	2.34
Diabetes → Hypertension	7.48	Hypertension → Hyperuricemia → CKD	2.71	CHD → Hypertension	5.92	Hypertension → CHD → Stroke	1.92
CHD → Hypertension	6.98	Diabetes → Hypertension → Hyperuricemia	1.95	Hypertension → Gout	4.68	Hypertension → Diabetes → Stroke	1.65
Hypertension → Stroke	6.52	Hypertension → Stroke → Diabetes	1.87	Diabetes → CHD	4.26	Hypertension → Hyperuricemia → Gout	1.65
Hypertension → CKD	5.39	Hypertension → Diabetes → CKD	1.70	Diabetes → Hyperuricemia	4.14	Hypertension → Hyperuricemia → CHD	1.65
Hypertension → Anemia	4.39	Hypertension → Gallbladder diseases → Hyperuricemia	1.61	Hypertension → Arrhythmia	3.73	Hypertension → Hyperuricemia → Diabetes	1.51
**Southern provinces**				**Southern provinces**			
Hypertension → Hyperuricemia	28.04	Hypertension → Diabetes → Hyperuricemia	6.51	Hypertension → Diabetes	18.88	Hypertension → CHD → Diabetes	3.51
Hypertension → Diabetes	17.25	Hypertension → Hyperuricemia → CKD	4.34	Hypertension → Hyperuricemia	17.22	Hypertension → Stroke → Diabetes	3.16
Diabetes → Hyperuricemia	7.83	Hypertension → CHD → Hyperuricemia	3.90	Hypertension → CHD	10.73	Hypertension → Diabetes → Hyperuricemia	2.81
Hypertension → CHD	7.51	Hypertension → Gout → CKD	3.04	Hypertension → Stroke	8.16	Hypertension → CHD → Hyperuricemia	2.81
Hypertension → CKD	7.30	Hypertension → CHD → Diabetes	2.82	Hypertension → Gout	7.10	Hypertension → Diabetes → CHD	2.81
Diabetes → Hypertension	6.24	Hypertension → Stroke → Hyperuricemia	2.60	Diabetes → Hypertension	4.98	Hypertension → Gallbladder diseases → Hyperuricemia	2.11
Hypertension → Stroke	5.71	Hypertension → Gallbladder diseases → Hyperuricemia	2.60	Diabetes → Hyperuricemia	4.53	Hypertension → Hyperuricemia → Gout	2.11
Hypertension → Anemia	5.19	CHD → Hypertension → Hyperuricemia	2.39	Hypertension → Gallbladder diseases	4.38	Hypertension → Hyperuricemia → CHD	2.11
CHD → Hyperuricemia	5.08	Hypertension → Stroke → Diabetes	2.17	Gallbladder diseases → Hypertension	3.93	Hypertension → Diabetes → Stroke	1.75
Hypertension → Gout	4.97	Hypertension → Diabetes → CKD	2.17	Diabetes → CHD	3.63	Gallbladder diseases → Hypertension →	1.75

						Hyperuricemia	
**Northern provinces**				**Northern provinces**			
Hypertension → Hyperuricemia	24.24	Hypertension → CHD → Hyperuricemia	7.10	Hypertension → CHD	14.61	Hypertension → CHD → Hyperuricemia	3.61
Hypertension → Diabetes	12.02	Hypertension → Diabetes → Hyperuricemia	5.57	Hypertension → Diabetes	13.34	Hypertension → CHD → Diabetes	3.39
Hypertension → CHD	10.36	Hypertension → Stroke → Hyperuricemia	3.20	Hypertension → Stroke	9.93	Hypertension → CHD → Stroke	2.48
CHD → Hyperuricemia	9.53	CHD → Hypertension → Hyperuricemia	3.06	Hypertension → Hyperuricemia	7.89	Hypertension → Diabetes → CHD	2.48
CHD → Hypertension	9.19	Hypertension → CHD → Diabetes	2.92	CHD → Hypertension	7.50	Hypertension → Diabetes → Hyperuricemia	2.26
Diabetes → Hyperuricemia	8.36	Diabetes → Hypertension → Hyperuricemia	2.09	Diabetes → Hypertension	7.21	Hypertension → Stroke → Diabetes	1.81
Diabetes → Hypertension	8.29	Hypertension → Arrhythmia → Hyperuricemia	2.09	Diabetes → CHD	4.67	Diabetes → Hypertension → CHD	1.81
Hypertension → Stroke	7.04	Diabetes → CHD → Hypertension	1.81	Spinal disease → Hypertension	4.19	Hypertension → Diabetes → Stroke	1.58
Stroke → Hyperuricemia	4.35	Diabetes → CHD → Hyperuricemia	1.81	Hypertension → Arrhythmia	3.99	Hypertension → Stroke → Hyperuricemia	1.58
Stroke → Hypertension	4.28	CHD → Diabetes → Hyperuricemia	1.81	Diabetes → Hyperuricemia	3.89	Hypertension → Hyperuricemia → Diabetes	1.58

CHD, coronary heart disease; CKD, chronic kidney disease; CLD, chronic liver disease; COPD, chronic obstructive pulmonary disease; CTRD, connective tissue and rheumatic diseases.

eTable 28. Top 10 binary and ternary sequential patterns of multimorbidity among men

Overall (self-reported + survey-detected)				Self-reported			
**Binary sequences**	**Support (%)**	**Ternary sequences**	**Support (%)**	**Binary sequences**	**Support (%)**	**Ternary sequences**	**Support (%)**
**Total**				**Total**			
Hypertension → Hyperuricemia	25.02	Hypertension → CHD → Hyperuricemia	3.67	Hypertension → Hyperuricemia	12.52	Hypertension → Diabetes → CHD	2.34
Hypertension → Diabetes	10.35	Hypertension → Diabetes → Hyperuricemia	3.14	Hypertension → Diabetes	10.77	Hypertension → Hyperuricemia → Gout	1.95
Hyperuricemia → Hypertension	6.73	Hypertension → Hyperuricemia → CKD	2.17	Hypertension → CHD	8.26	Hypertension → CHD → Diabetes	1.82
Diabetes → Hypertension	6.57	Hypertension → Stroke → Hyperuricemia	2.02	Hypertension → Gout	6.98	Hypertension → CHD → Hyperuricemia	1.69
Diabetes → Hyperuricemia	5.72	CHD → Hypertension → Hyperuricemia	1.95	Hypertension → Stroke	6.16	Hypertension → Hyperuricemia → CHD	1.56
Hypertension → CHD	5.04	CLD → Hypertension → Hyperuricemia	1.95	Diabetes → Hypertension	6.00	Hypertension → Diabetes → Stroke	1.43
CHD → Hyperuricemia	4.50	Diabetes → Hypertension → Hyperuricemia	1.87	Hyperuricemia → Hypertension	4.98	Diabetes → Hypertension → Hyperuricemia	1.43
Kidney stones → Hypertension	4.41	Kidney stones → Hypertension → Hyperuricemia	1.72	Gout → Hypertension	4.00	CLD → Hypertension → Hyperuricemia	1.30
Kidney stones → Hyperuricemia	4.38	Hypertension → Kidney stones → Hyperuricemia	1.72	Kidney stones → Hypertension	3.64	Hypertension → Hyperuricemia → Diabetes	1.30
Gout → Hypertension	4.32	Spinal disease → Hypertension → Hyperuricemia	1.65	Hypertension → Kidney stones	3.34	Hypertension → Gout → CHD	1.30
**Southern provinces**				**Southern provinces**			
Hypertension → Hyperuricemia	24.97	Hypertension → Diabetes → Hyperuricemia	3.31	Hypertension → Hyperuricemia	14.30	Hypertension → Hyperuricemia → Gout	1.89
Hypertension → Diabetes	11.51	Hypertension → Hyperuricemia → CKD	2.86	Hypertension → Diabetes	11.93	Hypertension → Diabetes → CHD	1.89
Hyperuricemia → Hypertension	6.73	Hypertension → Kidney stones → Hyperuricemia	2.11	Hypertension → Gout	8.85	Hypertension → Diabetes → Stroke	1.62
Diabetes → Hypertension	5.94	Hypertension → Gallbladder diseases → Hyperuricemia	2.11	Hypertension → CHD	5.35	Hypertension → Kidney stones → Hyperuricemia	1.62
Diabetes → Hyperuricemia	5.32	Hypertension → Gout → CKD	2.11	Diabetes → Hypertension	5.14	Hypertension → CHD → Hyperuricemia	1.62
Hypertension → Gout	5.26	Diabetes → Hypertension → Hyperuricemia	1.80	Hyperuricemia → Hypertension	4.73	Hypertension → Diabetes → Kidney stones	1.35
Kidney stones → Hyperuricemia	4.83	Hypertension → CHD → Diabetes	1.80	Hypertension → Stroke	4.53	CHD → Hypertension → Hyperuricemia	1.35
Kidney stones → Hypertension	4.59	Hypertension → Hyperuricemia → Diabetes	1.65	Hypertension → Kidney stones	4.42	Hypertension → Kidney stones → Gout	1.35
Hypertension → CKD	4.04	CLD → Hypertension → Hyperuricemia	1.50	Kidney stones → Hypertension	3.70	Hypertension → CHD → Gout	1.35
Gout → Hypertension	3.86	Hypertension → Stroke → Hyperuricemia	1.35	Hypertension → Gallbladder diseases	3.60	Hypertension → Stroke → Diabetes	1.35
**Northern provinces**				**Northern provinces**			
Hypertension → Hyperuricemia	25.08	Hypertension → CHD → Hyperuricemia	5.51	Hypertension → CHD	11.16	Hypertension → Diabetes → CHD	2.76
Hypertension → Diabetes	9.15	Hypertension → Diabetes → Hyperuricemia	2.98	Hypertension → Hyperuricemia	10.75	Hypertension → CHD → Diabetes	2.26

Diabetes → Hypertension	7.23	Hypertension → Stroke → Hyperuricemia	2.68	Hypertension → Diabetes	9.62	Hypertension → Hyperuricemia → CHD	2.26
Hypertension → CHD	6.97	CHD → Hypertension → Hyperuricemia	2.53	Hypertension → Stroke	7.78	Hypertension → Hyperuricemia → Gout	2.01
Hyperuricemia → Hypertension	6.72	CLD → Hypertension → Hyperuricemia	2.38	Diabetes → Hypertension	6.86	Diabetes → Hypertension → Hyperuricemia	1.76
CHD → Hyperuricemia	6.59	Spinal disease → Hypertension → Hyperuricemia	2.24	Hyperuricemia → Hypertension	5.22	Hypertension → CHD → Hyperuricemia	1.76
Diabetes → Hyperuricemia	6.14	Hypertension → CHD → Diabetes	2.24	Hypertension → Gout	5.12	CLD → Hypertension → Hyperuricemia	1.76
CHD → Hypertension	4.99	Kidney stones → Hypertension → Hyperuricemia	2.09	Gout → Hypertension	4.61	Hypertension → Gout → CHD	1.76
Hypertension → Stroke	4.86	Diabetes → Hypertension → Hyperuricemia	1.94	Spinal disease → Hypertension	4.30	Hypertension → Stroke → CHD	1.51
Gout → Hypertension	4.80	Hypertension → CLD → Hyperuricemia	1.64	Diabetes → CHD	4.09	Diabetes → Hypertension → CHD	1.51

CHD, coronary heart disease; CKD, chronic kidney disease; CLD, chronic liver disease; COPD, chronic obstructive pulmonary disease; CTRD, connective tissue and rheumatic diseases.

eTable 29. Top 10 binary and ternary sequential patterns of multimorbidity among women

Overall (self-reported + survey-detected)				Self-reported			
**Binary sequences**	**Support (%)**	**Ternary sequences**	**Support (%)**	**Binary sequences**	**Support (%)**	**Ternary sequences**	**Support (%)**
**Total**				**Total**			
Hypertension → Hyperuricemia	23.54	Hypertension → Diabetes → Hyperuricemia	7.33	Hypertension → Diabetes	13.75	Hypertension → Diabetes → Hyperuricemia	2.97
Hypertension → Diabetes	10.91	Hypertension → CHD → Hyperuricemia	4.70	Hypertension → CHD	9.81	Hypertension → Diabetes → CHD	2.73
Diabetes → Hyperuricemia	7.64	Hypertension → Stroke → Hyperuricemia	2.77	Hypertension → Hyperuricemia	9.20	Hypertension → CHD → Hyperuricemia	2.60
Diabetes → Hypertension	6.24	Diabetes → Hypertension → Hyperuricemia	2.56	Hypertension → Stroke	6.61	Hypertension → CHD → Diabetes	2.48
Hypertension → CHD	5.92	CHD → Hypertension → Hyperuricemia	2.42	Diabetes → Hypertension	6.40	Hypertension → Stroke → Diabetes	2.11
CHD → Hyperuricemia	5.73	Hypertension → CHD → Diabetes	1.73	CHD → Hypertension	6.04	Hypertension → Diabetes → Stroke	1.73
CHD → Hypertension	5.23	CHD → Diabetes → Hyperuricemia	1.59	Spinal disease → Hypertension	3.85	Hypertension → CHD → Stroke	1.61
Hypertension → Stroke	3.99	Hypertension → Diabetes → CHD	1.52	Diabetes → Hyperuricemia	3.46	Diabetes → Hypertension → CHD	1.61
Spinal disease → Hypertension	3.59	Hypertension → Spinal disease → Hyperuricemia	1.45	Diabetes → CHD	3.29	CHD → Hypertension → Diabetes	1.36
Hypertension → Anemia	3.38	Hypertension → Stroke → Diabetes	1.45	Hypertension → Spinal disease	3.02	Hypertension → Hyperuricemia → Diabetes	1.24
**Southern provinces**				**Southern provinces**			
Hypertension → Hyperuricemia	23.03	Hypertension → Diabetes → Hyperuricemia	3.31	Hypertension → Diabetes	14.70	Hypertension → Diabetes → Hyperuricemia	3.61
Hypertension → Diabetes	10.81	Hypertension → Hyperuricemia → CKD	2.86	Hypertension → Hyperuricemia	13.36	Hypertension → Stroke → Diabetes	2.53
Diabetes → Hyperuricemia	6.63	Hypertension → Kidney stones → Hyperuricemia	2.11	Hypertension → CHD	6.51	Hypertension → CHD → Diabetes	2.53
Diabetes → Hypertension	5.53	Hypertension → Gallbladder diseases → Hyperuricemia	2.11	Diabetes → Hypertension	5.72	Hypertension → CHD → Hyperuricemia	1.81
Hypertension → Anemia	4.55	Hypertension → Gout → CKD	2.11	Hypertension → Stroke	5.50	Hypertension → Hyperuricemia → CHD	1.81
Gallbladder diseases → Hypertension	3.99	Diabetes → Hypertension → Hyperuricemia	1.80	Diabetes → Hyperuricemia	4.26	Hypertension → Diabetes → CHD	1.81
Hypertension → CHD	3.56	Hypertension → CHD → Diabetes	1.80	Gallbladder diseases → Hypertension	3.48	Gallbladder diseases → Hypertension → Hyperuricemia	1.44
Hypertension → Stroke	3.01	Hypertension → Hyperuricemia → Diabetes	1.65	Hypertension → Gallbladder diseases	3.03	Diabetes → Hypertension → Hyperuricemia	1.44
Spinal disease → Hypertension	2.89	CLD → Hypertension → Hyperuricemia	1.50	Hypertension → Spinal disease	2.92	Hypertension → Stroke → CHD	1.44

Kidney stones → Hyperuricemia	2.52	Hypertension → Stroke → Hyperuricemia	1.35	CHD → Hypertension	2.81	Hypertension → Diabetes → Stroke	1.08
**Northern provinces**				**Northern provinces**			
Hypertension → Hyperuricemia	23.92	Hypertension → Diabetes → Hyperuricemia	6.73	Hypertension → Diabetes	13.15	Hypertension → Diabetes → CHD	3.21
Hypertension → Diabetes	10.99	Hypertension → CHD → Hyperuricemia	2.69	Hypertension → CHD	11.93	Hypertension → CHD → Hyperuricemia	3.02
CHD → Hyperuricemia	8.39	Diabetes → Hypertension → Hyperuricemia	2.50	CHD → Hypertension	8.12	Hypertension → Diabetes → Hyperuricemia	2.64
Diabetes → Hyperuricemia	8.39	Hypertension → Stroke → Hyperuricemia	1.92	Hypertension → Stroke	7.33	Hypertension → CHD → Stroke	2.45
Hypertension → CHD	7.70	Hypertension → Diabetes → Anemia	1.73	Diabetes → Hypertension	6.82	Hypertension → CHD → Diabetes	2.45
CHD → Hypertension	7.51	Kidney stones → Hypertension → Hyperuricemia	1.54	Hypertension → Hyperuricemia	6.54	Diabetes → Hypertension → CHD	2.26
Diabetes → Hypertension	6.77	Gallbladder diseases → Hypertension → Hyperuricemia	1.54	Spinal disease → Hypertension	4.74	Hypertension → Diabetes → Stroke	2.08
Hypertension → Stroke	4.73	Hypertension → CHD → Diabetes	1.54	Diabetes → CHD	4.02	Hypertension → Stroke → Diabetes	1.89
Spinal disease → Hypertension	4.13	Hypertension → Hyperuricemia → CKD	1.54	CHD → Diabetes	3.52	CHD → Hypertension → Diabetes	1.89
Spinal disease → Hyperuricemia	3.38	Hypertension → Stroke → Diabetes	1.35	Hypertension → Arrhythmia	3.16	Spinal disease → CHD → Hypertension	1.51

CHD, coronary heart disease; CKD, chronic kidney disease; CLD, chronic liver disease; COPD, chronic obstructive pulmonary disease; CTRD, connective tissue and rheumatic diseases.

eTable 30. Prevalence of five chronic conditions: Overall (self-reported + survey-detected) and survey-detected only

**Chronic conditions**	**Total (n=19,067)**		**Southern provinces (n=9,908)**		**Northern provinces (n=9,159)**	
	**Overall**	**Survey-detected**	**Overall**	**Survey-detected**	**Overall**	**Survey-detected**
Hypertension	7,637 (40.05)	2,418 (12.68)	3,745 (37.80)	1,279 (12.91)	3,892 (42.49)	1,139 (12.44)
Diabetes	2,559 (13.42)	607 (3.18)	1,225 (12.36)	316 (3.19)	1,334 (14.56)	291 (3.18)
Hyperuricemia	5,540 (29.06)	4,033 (21.15)	2,817 (28.43)	1,994 (20.13)	2,723 (29.73)	2,039 (22.26)
CKD	343 (1.80)	275 (1.44)	163 (1.65)	143 (1.44)	180 (1.97)	132 (1.44)
Anemia	1,166 (6.12)	972 (5.10)	749 (7.56)	599 (6.05)	417 (4.55)	373 (4.07)

CKD, chronic kidney disease.

eTable 31. Top 10 binary association rules of multimorbidity

Overall (self-reported + survey-detected)					Self-reported				
**Antecedent**	**Consequent**	**Support (%)**	**Confidence (%)**	**Lift**	**Antecedent**	**Consequent**	**Support (%)**	**Confidence (%)**	**Lift**
**Total**					**Total**				
CKD	Gout	0.94	20.99	2.42	Gout	Hyperuricemia	12.91	94.77	3.53
CKD	Anemia	0.90	20.06	2.41	Osteoarthritis	Spinal disease	0.58	25.49	2.10
Gout	Hyperuricemia	8.45	97.29	1.81	Stroke	Hypertension	10.49	80.79	1.15
Stroke	CHD	2.00	22.68	1.43	CTRD	CHD	0.62	25.93	1.14
CKD	Hyperuricemia	3.34	74.38	1.38	CKD	Hypertension	0.91	78.85	1.13
CKD	Diabetes	1.51	33.64	1.14	Cancer	Diabetes	2.09	35.74	1.10
Stroke	Hypertension	7.25	82.36	1.10	Stroke	CHD	3.21	24.70	1.08
CKD	Hypertension	3.56	79.32	1.06	Diabetes	Hypertension	23.98	73.77	1.05
Diabetes	Hypertension	22.82	77.14	1.03	Hypertension	Diabetes	23.98	34.22	1.05
Hypertension	Diabetes	22.82	30.47	1.03	CHD	Hypertension	16.12	70.77	1.01
**Southern provinces**					**Southern provinces**				
CKD	Gout	1.18	26.14	2.60	Anemia	Arrhythmia	0.50	20.41	3.58
CKD	Anemia	1.07	23.53	2.21	Gout	Hyperuricemia	15.63	94.51	2.90
Gout	Hyperuricemia	9.74	96.76	1.76	CKD	Hypertension	0.71	93.33	1.34
CKD	Hyperuricemia	3.29	72.55	1.32	Stroke	Hypertension	7.61	82.97	1.19
Stroke	Hypertension	5.06	86.8	1.15	Cancer	Diabetes	1.92	36.19	1.07
CHD	Diabetes	2.93	34.49	1.14	Diabetes	Hypertension	24.90	73.73	1.06
CKD	Diabetes	1.54	33.99	1.12	Hypertension	Diabetes	24.90	35.77	1.06
Cancer	Diabetes	1.33	32.85	1.09	CHD	Hypertension	9.78	72.93	1.05
CKD	Hypertension	3.61	79.74	1.06					
Stroke	Diabetes	1.87	31.98	1.06					
**Northern provinces**					**Northern provinces**				
Osteoarthritis	Spinal disease	0.52	24.69	2.22	Gout	Hyperuricemia	10.77	95.07	4.27
Gout	Hyperuricemia	7.32	97.91	1.85	Osteoarthritis	Spinal disease	0.80	27.78	1.92

CKD	Hyperuricemia	3.39	76.02	1.44	CKD	Hyperuricemia	0.56	37.84	1.70
CTRD	CHD	0.63	28.24	1.26	Stroke	Hypertension	12.76	79.80	1.13
Osteoarthritis	CHD	0.55	25.93	1.16	Cancer	Diabetes	2.23	35.44	1.13
CKD	Diabetes	1.48	33.33	1.15	Diabetes	Hypertension	23.25	73.80	1.05
Stroke	CHD	2.84	24.89	1.11	Hypertension	Diabetes	23.25	33.01	1.05
Stroke	Hypertension	9.17	80.37	1.08	CKD	Hypertension	1.08	72.97	1.04
Chronic gastritis	CHD	1.22	24.10	1.08	CTRD	CHD	0.96	31.17	1.03
CKD	Hypertension	3.52	78.95	1.06	Kidney stones	Hyperuricemia	1.67	22.70	1.02

CHD, coronary heart disease; CKD, chronic kidney disease; CLD, chronic liver disease; COPD, chronic obstructive pulmonary disease; CTRD, connective tissue and rheumatic diseases.

eTable 32. Top 10 binary and ternary sequential patterns of multimorbidity

Overall (self-reported + survey-detected)				Self-reported			
**Binary sequences**	**Support (%)**	**Ternary sequences**	**Support (%)**	**Binary sequences**	**Support (%)**	**Ternary sequences**	**Support (%)**
**Total**				**Total**			
Hypertension → Hyperuricemia	24.22	Hypertension → Diabetes → Hyperuricemia	5.32	Hypertension → Diabetes	12.38	Hypertension → Diabetes → CHD	2.54
Hypertension → Diabetes	10.66	Hypertension → CHD → Hyperuricemia	4.21	Hypertension → Hyperuricemia	10.73	Hypertension → CHD → Hyperuricemia	2.16
Diabetes → Hyperuricemia	6.76	Hypertension → Stroke → Hyperuricemia	2.41	Hypertension → CHD	9.10	Hypertension → CHD → Diabetes	2.16
Diabetes → Hypertension	6.39	Diabetes → Hypertension → Hyperuricemia	2.23	Hypertension → Stroke	6.40	Hypertension → Diabetes → Hyperuricemia	2.10
Hypertension → CHD	5.51	CHD → Hypertension → Hyperuricemia	2.19	Diabetes → Hypertension	6.21	Hypertension → Diabetes → Stroke	1.59
CHD → Hyperuricemia	5.17	Hypertension → CHD → Diabetes	1.69	CHD → Hypertension	4.58	Hypertension → Stroke → Diabetes	1.59
CHD → Hypertension	4.43	Hypertension → Hyperuricemia → CKD	1.65	Hypertension → Gout	4.44	Diabetes → Hypertension → CHD	1.46
Hyperuricemia → Hypertension	4.15	CLD → Hypertension → Hyperuricemia	1.58	Spinal disease → Hypertension	3.52	Hypertension → CHD → Stroke	1.33
Hypertension → Stroke	3.88	Hypertension → Diabetes → CHD	1.44	Diabetes → Hyperuricemia	3.40	Diabetes → Hypertension → Hyperuricemia	1.27
Spinal disease → Hypertension	3.32	Spinal disease → Hypertension → Hyperuricemia	1.37	Diabetes → CHD	3.21	Hypertension → Hyperuricemia → Diabetes	1.27
**Southern provinces**				**Southern provinces**			
Hypertension → Hyperuricemia	24.00	Hypertension → Diabetes → Hyperuricemia	4.81	Hypertension → Hyperuricemia	13.85	Hypertension → Diabetes → Hyperuricemia	2.16
Hypertension → Diabetes	11.16	Hypertension → Hyperuricemia → CKD	2.28	Hypertension → Diabetes	13.26	Hypertension → Stroke → Diabetes	1.85
Diabetes → Hyperuricemia	5.98	Hypertension → CHD → Hyperuricemia	2.19	Hypertension → Gout	5.96	Hypertension → CHD → Diabetes	1.85
Diabetes → Hypertension	5.73	Diabetes → Hypertension → Hyperuricemia	2.11	Hypertension → CHD	5.90	Hypertension → Diabetes → CHD	1.85
Hyperuricemia → Hypertension	4.41	Hypertension → Gallbladder diseases → Hyperuricemia	1.69	Diabetes → Hypertension	5.42	Hypertension → CHD → Hyperuricemia	1.70
Kidney stones → Hyperuricemia	3.68	Hypertension → Stroke → Hyperuricemia	1.60	Hypertension → Stroke	4.99	Hypertension → Diabetes → Stroke	1.39
Kidney stones → Hypertension	3.43	Hypertension → Kidney stones → Hyperuricemia	1.52	Hypertension → Kidney stones	3.54	Hypertension → Hyperuricemia → Gout	1.39
Hypertension → Anemia	3.43	Hypertension → Hyperuricemia → Diabetes	1.52	Diabetes → Hyperuricemia	3.54	Hypertension → Diabetes → Kidney stones	1.24
Hypertension → Gout	3.4	Hypertension → Gout → CKD	1.52	Hypertension → Gallbladder diseases	3.33	Hypertension → Kidney stones → Hyperuricemia	1.24
Hypertension → CHD	3.37	Kidney stones → Hypertension → Hyperuricemia	1.43	Hyperuricemia → Hypertension	3.22	Diabetes → Hypertension → Hyperuricemia	1.24
**Northern provinces**				**Northern provinces**			
Hypertension → Hyperuricemia	24.41	Hypertension → Diabetes → Hyperuricemia	5.70	Hypertension → Diabetes	11.69	Hypertension → Diabetes → CHD	3.02
Hypertension → Diabetes	10.22	Hypertension → CHD → Hyperuricemia	5.70	Hypertension → CHD	11.61	Hypertension → CHD → Hyperuricemia	2.48

CHD → Hyperuricemia	7.63	Hypertension → Stroke → Hyperuricemia	3.01	Hypertension → Hyperuricemia	8.27	Hypertension → CHD → Diabetes	2.37
Diabetes → Hyperuricemia	7.45	CHD → Hypertension → Hyperuricemia	2.88	Hypertension → Stroke	7.51	Hypertension → Diabetes → Hyperuricemia	2.05
Hypertension → Hyperuricemia	7.39	Diabetes → Hypertension → Hyperuricemia	2.32	Diabetes → Hypertension	6.84	Hypertension → CHD → Stroke	1.94
Diabetes → Hypertension	6.96	Hypertension → CHD → Diabetes	2.00	CHD → Hypertension	6.42	Diabetes → Hypertension → CHD	1.94
CHD → Hypertension	6.45	CLD → Hypertension → Hyperuricemia	1.82	Spinal disease → Hypertension	4.56	Hypertension → Diabetes → Stroke	1.72
Hypertension → Stroke	4.78	Hypertension → Diabetes → CHD	1.75	Diabetes → CHD	4.05	Hypertension → Stroke → Diabetes	1.40
Spinal disease → Hypertension	4.14	Spinal disease → Hypertension → Hyperuricemia	1.63	Diabetes → Hyperuricemia	3.29	Diabetes → Hypertension → Hyperuricemia	1.29
Hyperuricemia → Hypertension	3.92	CHD → Diabetes → Hyperuricemia	1.57	Hypertension → Gout	3.25	Hypertension → Hyperuricemia → Diabetes	1.29

CHD, coronary heart disease; CKD, chronic kidney disease; CLD, chronic liver disease; COPD, chronic obstructive pulmonary disease; CTRD, connective tissue and rheumatic diseases.

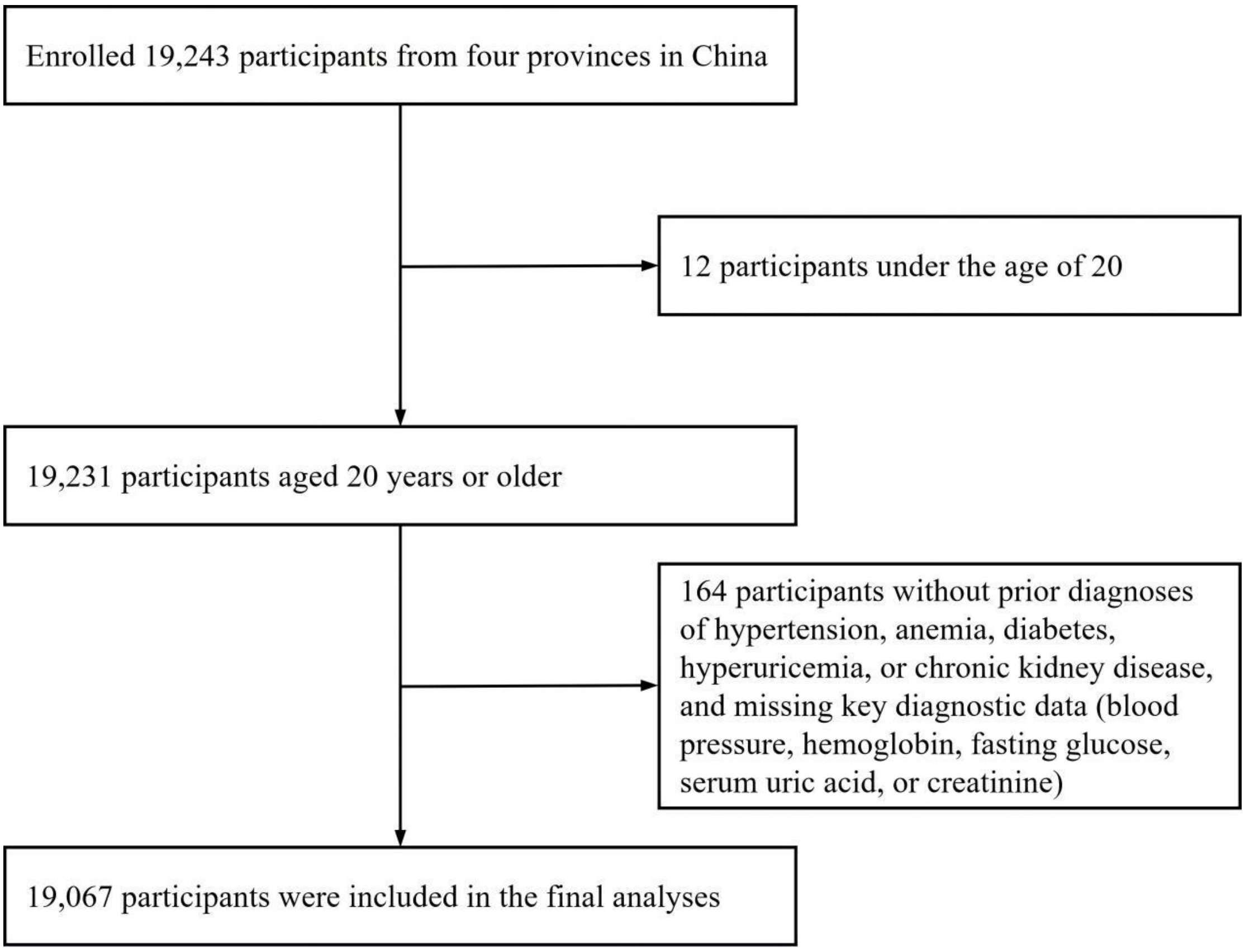

eFigure 1. Participant flow diagram
